# Merger of Visible Light‐Driven Chiral Organocatalysis and Continuous Flow Chemistry: An Accelerated and Scalable Access into Enantioselective α‐Alkylation of Aldehydes

**DOI:** 10.1002/adsc.202300289

**Published:** 2023-05-16

**Authors:** Márk Molnár, C. Oliver Kappe, Sándor B. Ötvös

**Affiliations:** ^1^ Institute of Chemistry University of Graz NAWI Graz, Heinrichstrasse 28 A-8010 Graz Austria; ^2^ Servier Research Institute of Medicinal Chemistry Záhony u. 7 1031 Budapest Hungary; ^3^ Center for Continuous Flow Synthesis and Processing (CC FLOW) Research Center Pharmaceutical Engineering GmbH (RCPE) Inffeldgasse 13 A-8010 Graz Austria

**Keywords:** α-Alkylation, Asymmetric synthesis, Continuous flow, Organocatalysis, Photochemistry

## Abstract

The electron donor‐acceptor complex‐enabled asymmetric photochemical alkylation strategy holds potential to attain elusive chiral α‐alkylated aldehydes without an external photoredox catalyst. The photosensitizer‐free conditions are beneficial concerning process costs and sustainability. However, lengthy organocatalyst preparation steps as well as limited productivity and difficult scalability render the current approaches unsuitable for synthesis on enlarged scales. Inspired by these limitations, a protocol was developed for the enantioselective α‐alkylation of aldehydes based on the synergistic combination of visible light‐driven asymmetric organocatalysis and a controlled continuous flow reaction environment. With the aim to reduce process costs, a commercially available chiral catalyst has been exploited to achieve photosensitizer‐free enantioselective α‐alkylations using phenacyl bromide derivates as alkylating agents. As a result of elaborate optimization and process development, the present flow strategy furnishes an accelerated and inherently scalable entry into enantioenriched α‐alkylated aldehydes including a chiral key intermediate of the antirheumatic esonarimod.

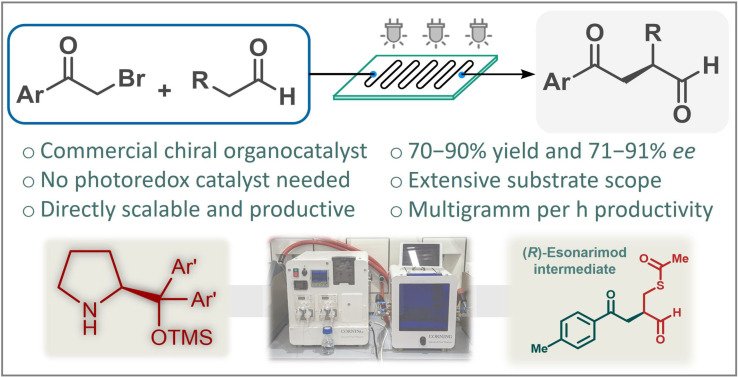

## Introduction

The asymmetric α‐alkylation of carbonyl compounds is one of the most important synthetic tools to construct molecular complexity by novel stereogenic centers.^[1]^ Traditional strategies rely on S_N_2‐type reactions of preformed metal‐enolate nucleophiles and different alkyl halides in the presence of stoichiometric amounts of chiral auxiliaries to introduce asymmetry.^[2]^ Considering that the utilization of metal‐enolates and chiral auxiliaries requires additional synthetic steps and implies high costs and considerable waste generation, extensive efforts have been made towards the development of catalytic asymmetric protocols for the direct α‐alkylation of unmodified carbonyl substrates.^[1]^ In this manner, enamine‐mediated activation of aldehydes by chiral amines as organocatalysts was found particularly promising. For example, in 2004 Vignola and List presented a powerful approach for the catalytic enantioselective intramolecular α‐alkylation of haloaldehydes in the presence of α‐methyl proline as organocatalyst,^[3]^ and this strategy was later exploited as a key step of various asymmetric cascade reactions.^[4]^ Besides these intramolecular S_N_2 transformation‐based processes, enamine‐mediated α‐alkylation of aldehydes also took advantage of an S_N_2’‐type addition‐elimination pathway involving ammonium salts as intermediates,^[5]^ and S_N_1‐type reactions between stabilized carbocations and enamines also demonstrated significant potential for the enantioselective construction of α‐alkylated aldehydes.^[6]^ Despite the considerable progress made, asymmetric α‐alkylation reactions were still strongly limited in scope and applicability. This was mainly due to the modest reactivity of alkyl halides, and also because of the fact that such reactions of preformed aldehyde enolates or enamines are prone to numerous side reactions, such as self‐aldol condensation, Cannizzaro or Tischenko reactions, and O‐ or N‐alkylations of the catalyst by the electrophilic alkyl halide component.^[1]^ Moreover, catalytic enantioselective approaches for the α‐alkylation of aldehydes typically required relatively high catalyst loadings in combination with low temperatures to avoid the racemization of the newly formed stereogenic center,[^4^, ^5^, ^6^] and in S_N_1‐type reactions, special substrates and/or reaction conditions were needed to stabilize the carbocations.^[6]^

**Scheme 1:**
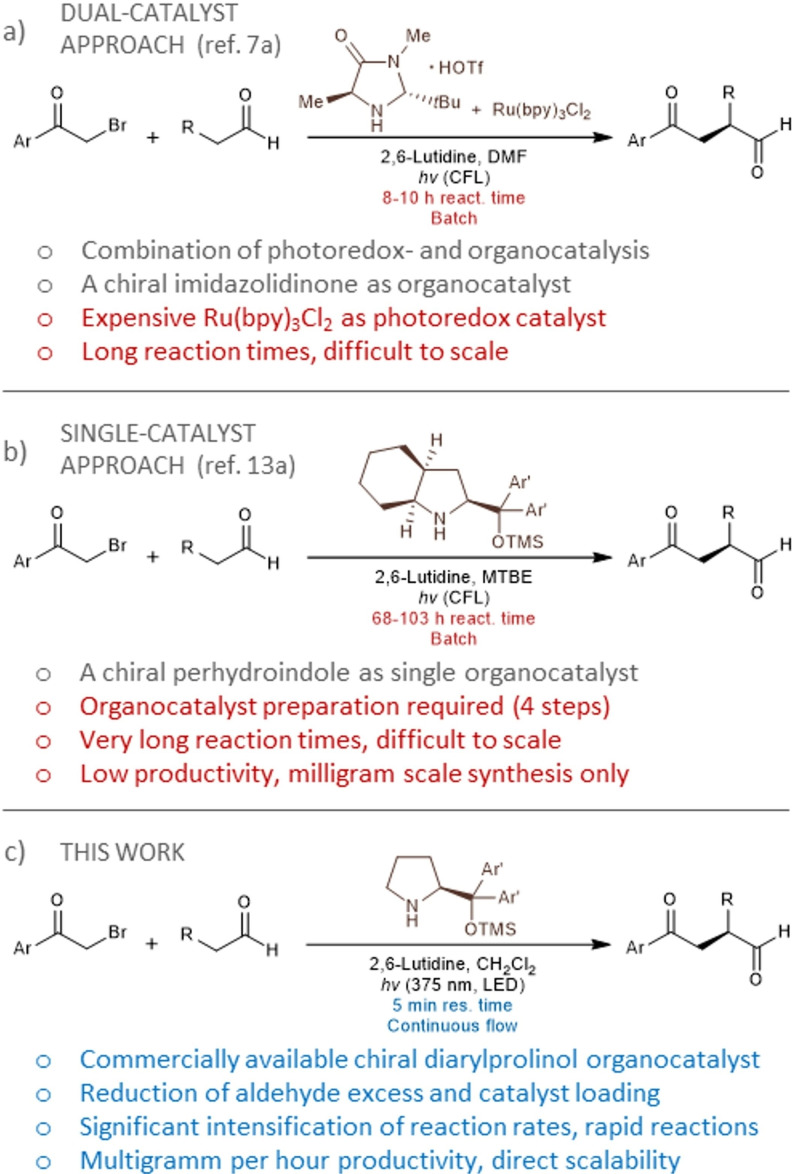
Literature approaches for visible light‐driven enantioselective α‐alkylation of aldehydes vs. this work.

The hurdles associated with the limited reactivity of alkyl halides prone to participate in side reactions were overcame by Nicewitz and MacMillan via synergistic combination of photoredox‐ and organocatalysis (Scheme 1a).^[7]^ In addition to conventional enamine activation of the aldehyde component with a chiral imidazolidinone catalyst, their strategy relied on the utilization of alkyl bromides not as electrophiles as in earlier approaches, but as precursors for generating radicals in the presence of a ruthenium‐based polypyridyl photocatalyst and visible light irradiation. In this manner, a ground state aminocatalytic cycle, which is responsible for asymmetric induction, worked synergistically with a photoredox catalytic system, and electron‐deficient radicals could rapidly and selectively react with electron rich enamines enabling the formation of elusive α‐alkylated aldehydes.^[8]^

Since the pioneering discovery of Nicewitz and MacMillan, ruthenium‐based complexes have gained fundamental importance in homogeneous photoredox chemistry.^[9]^ However, ruthenium is amongst the rarest elements on Earth, and ruthenium compounds are highly toxic and suspected carcinogens.^[10]^ Even though various organic dyes and inorganic semiconductors also showed potential as photocatalysts in asymmetric α‐alkylation of aldehydes,^[11]^ such photosensitizers are typically too expensive for manufacturing‐scale processes and significantly complicate product purification in various applications. Ideally, photochemical processes should be sustainable and economically reliable; thus the development of transition‐metal‐ and photosensitizer‐free protocols provides promising alternatives.^[12]^ To this end, Melchiorre and co‐workers introduced electron donor‐acceptor (EDA) complex‐directed enantioselective photochemistry and were able to access α‐alkylated aldehydes without an external photoredox catalyst (Scheme 1b).^[13]^ A chiral enamine generated *in situ* from the aldehyde component with an amine catalyst acted as electron donor in combination with an electron‐deficient alkyl bromide with high electronic affinity (such as phenacyl bromides) to provide a transient EDA complex that can absorb visible light.^[14]^ Upon irradiation, an intermolecular single electron transfer occurs from the enamine to the alkyl bromide leading to a radical ion pair. This reacts with a ground state chiral enamine under effective stereochemical control thereby furnishing enantioselective α‐alkylation without the need for an exogenous photosensitizer.^[15]^

In the report by Melchiorre and co‐workers, a chiral perhydroindole derivative served as organocatalyst,^[13a]^ which was prepared in‐house from commercially available perhydroindole‐2‐carboxylic acid via four synthetic steps.^[16]^ The reactions were performed exclusively on milligram scale but required very long reaction times (up to 103 h) and a considerably large excess of the aldehyde component in combination with a relatively high catalyst loading of 20 mol%. Although the achievement of transition‐metal‐ and photosensitizer‐free conditions can be regarded as a very important benefit for asymmetric α‐alkylation chemistries, the limited productivity and the need for a specialized organocatalyst renders the current process impractical on preparatively significant scales and for potential production purposes. As defined by the Beer‐Lambert law, light penetration is dependent on the irradiated reactor size, therefore scalability is in fact one of the most critical aspects of photochemical transformations.^[17]^ In this manner, industrial applications have been hampered by limited light penetration, and have required special reactors and powerful light sources to manufacture large amounts of chemicals per unit of time.^[18]^ In contrast to conventional batch operations where scale‐up is primarily achieved by an increase in vessel size, continuous flow reactors ensure facile scalability by maintaining a short irradiated path length and a uniform light distribution in narrow reaction channels, whilst considerably increasing the efficacy of transformations leading to shorter reaction times, higher productivity and less side product formation.^[19]^

Despite its inherent benefits, flow chemistry has only scarcely been investigated for the catalytic enantioselective α‐alkylation of carbonyl compounds,^[20]^ and the asymmetric photo‐organocatalytic synthesis strategy has so far been achieved only in small‐scale batch reactors lacking efficient preparative applications. We speculated that flow chemistry might act as an enabling platform for the EDA complex‐directed photosensitizer‐free enantioselective α‐alkylation of aldehydes. By synergistic combination of visible light‐driven chiral organocatalysis and a precisely controlled continuous flow reaction environment, an intensified and inherently scalable entry was envisioned into valuable chiral α‐alkylated aldehydes (Scheme 1c). In order to reduce process costs and to increase the practical value for potential manufacturing purposes, we aimed for the utilization of commercially available chiral catalysts in combination with strict control over the most important reaction conditions. Our results are presented herein.

## Results and Discussion

On the basis of the synthetic usefulness of an additional carbonyl moiety, we selected phenacyl bromides as electron‐deficient alkylating agents for various aldehydes. The α‐alkylation of butyraldehyde with 2‐bromoacetophenone yielding (*R*)‐2‐ethyl‐4‐oxo‐4‐phenylbutanal (**3**) as product was selected as model reaction for process development and parameter screening. Our study was initiated with a preliminary batch screening in order to evaluate the feasibility of reaction conditions for continuous flow method development (Table 1). Commercially available diarylprolinol trimethylsilyl ethers **1** and **2** were explored as chiral organocatalysts. The reactions were performed in different solvents by irradiation using a 372 nm LED at ambient temperature (the exclusion of light is known to completely inhibit the reaction).^[13a]^ 2,6‐Lutidine served as base to quench any HBr generated during the process.^[14]^ In terms of conversion and chemical selectivity, catalyst **1** containing the 3,5‐bis(trifluoromethyl)phenyl moiety performed superior to diphenylprolinol **2** (entries 1–10 vs. entries 11 and 12). As concerns solvent effects, competing debromination of the alkylating agent was promoted significantly in MeOH and EtOH as verified by the presence of acetophenone (**3i**) together with some unidentified substances as side products (entries 1 and 2).^[21]^ Only very small amounts of acetophenone were detected in cases of all other solvents investigated; however, in MTBE, *i*PrOH, EtOAc and acetone, significant amounts of unidentified side product formation occurred (entries 3, 4 and 7). Not only the chemical selectivity, but also the *ee* of the reactions was found to strongly dependent on the solvents applied. *i*PrOH, MeCN and CH_2_Cl_2_ performed the best with *ee*s of 87%, 75% and 86%, respectively (entries 3, 6 and 10). Importantly, in many of the solvents, some extent of precipitation was observed making continuous flow process development impossible due to potential clogging issues. Gratifyingly, reaction mixtures containing MeCN and CH_2_Cl_2_ remained completely clear during the transformations rendering them applicable as solvents under flow conditions.

**Table 1:** Preliminary batch screening of chiral catalysts and solvents.

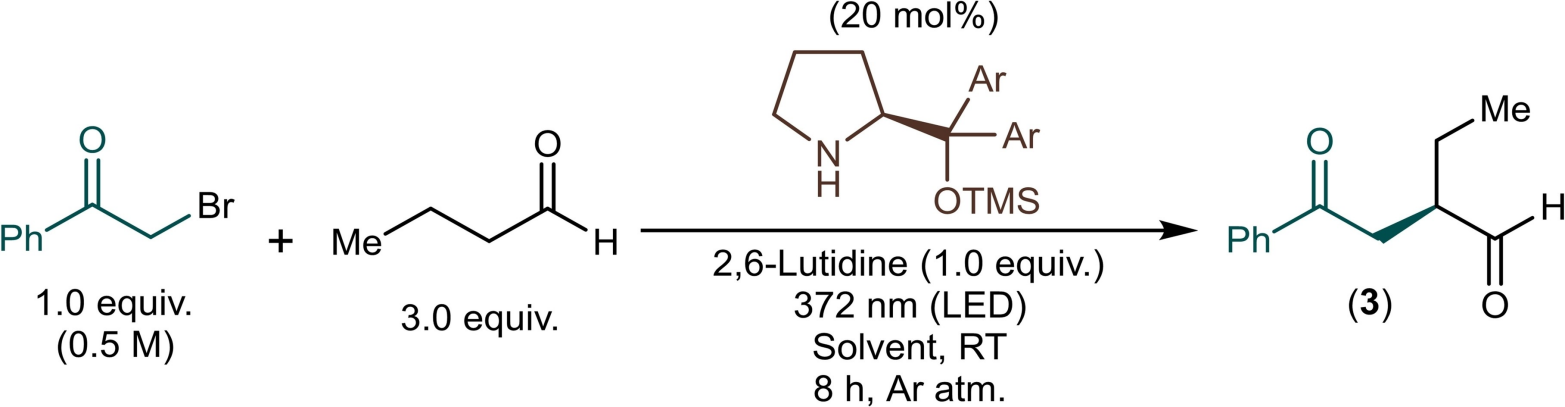							
#	Ar	Solvent	Conv. (%)^[a]^	Select. (%)^[a]^			*ee* (%)^[d]^
				(**3**)	(**3i**)^[b]^	(**3ii**)^[c]^	
1^[e]^	3,5‐(CF_3_)_2_‐C_6_H_3_ (**1**)	MeOH	100	54	19	27	n.d.
2^[e]^	3,5‐(CF_3_)_2_‐C_6_H_3_ (**1**)	EtOH	100	35	30	35	n.d.
3^[e]^	3,5‐(CF_3_)_2_‐C_6_H_3_ (**1**)	*i*PrOH	100	71	3	26	87
4^[f]^	3,5‐(CF_3_)_2_‐C_6_H_3_ (**1**)	MTBE	100	86	0	14	56
5^[f]^	3,5‐(CF_3_)_2_‐C_6_H_3_ (**1**)	2MeTHF	100	94	1	5	41
6^[g]^	3,5‐(CF_3_)_2_‐C_6_H_3_ (**1**)	MeCN	100	94	4	2	75
7^[f]^	3,5‐(CF_3_)_2_‐C_6_H_3_ (**1**)	EtOAc	100	89	1	10	71
8^[f]^	3,5‐(CF_3_)_2_‐C_6_H_3_ (**1**)	Acetone	100	89	1	10	69
9^[f]^	3,5‐(CF_3_)_2_‐C_6_H_3_ (**1**)	Toluene	100	98	1	1	66
10^[g]^	3,5‐(CF_3_)_2_‐C_6_H_3_ (**1**)	CH_2_Cl_2_	100	93	0	7	86
11^[g]^	C_6_H_5_ (**2**)	CH_2_Cl_2_	97	62	0	38	n.d.
12^[g]^	C_6_H_5_ (**2**)	MeCN	79	35	2	63	n.d.

^[a]^ Determined by HPLC area%. ^[b]^ **3 i**: acetophenone. ^[c]^ **3ii**: Unidentified side product(s). ^[d]^ Determined by chiral HPLC. ^[e]^ Cloudy mixture. ^[f]^ Precipitation occurred. ^[g]^ Clear solution.

The asymmetric photochemical aldehyde alkylation yields a configurationally labile α‐stereocenter that may racemize via enolization. The enantiomeric stability of compound **3** was therefore examined as a function of time under different conditions (Table S1 in the Supporting Information). It was found that even though racemization of pure **3** is slow at room temperature, in the presence of the secondary amine organocatalyst and/or the base, enolization is faster leading to a considerable decrease of *ee* via racemization. For example, in a 0.5 M CH_2_Cl_2_ solution of **3** in the presence of 20 mol% of catalyst **1** (same amount as in the reactions shown in Table 1), the *ee* decreased from 86% to 84% after 16 h, then to 71% after 4 days and finally to 41% after 8 days. As the chiral catalyst apparently promotes the enolization and hence the racemization of compound **3**, very long reaction times, typically employed in earlier batch approaches, should be considered disadvantageous in terms of the enantiomeric purity of the α‐alkylated product. In contrast, the well‐defined short process times under flow conditions may help mitigating the risk of product racemization.

With sufficient amount of data on the effects of various solvents and organocatalysts in hand, we next turned our attention to continuous flow method development. Flow experiments were performed in a Corning Advanced‐Flow Lab Photo Reactor comprising a glass plate‐based fluidic module (2.77 mL internal volume), a high‐capacity heat exchanger and two LED panels mounted on both sides of the fluidic module (full details and photographs of the flow setup are supplied in the Supporting Information). The choice of this particular lab‐scale system was justified by the smart dimensioning approach ensuring homogeneity of performance across the whole range from lab‐ to manufacturing‐scale systems.^[22]^ This mean that upon scaling‐up, the most important reaction parameters, such as residence time, heat and mass transfer are kept consistent, whilst throughput is increased proportionally.^[23]^ In particular, for photochemical applications, the increase in irradiated path length for larger reactors is also minimized thereby enabling direct scalability without the need for thorough reoptimization.

**Scheme 2:**
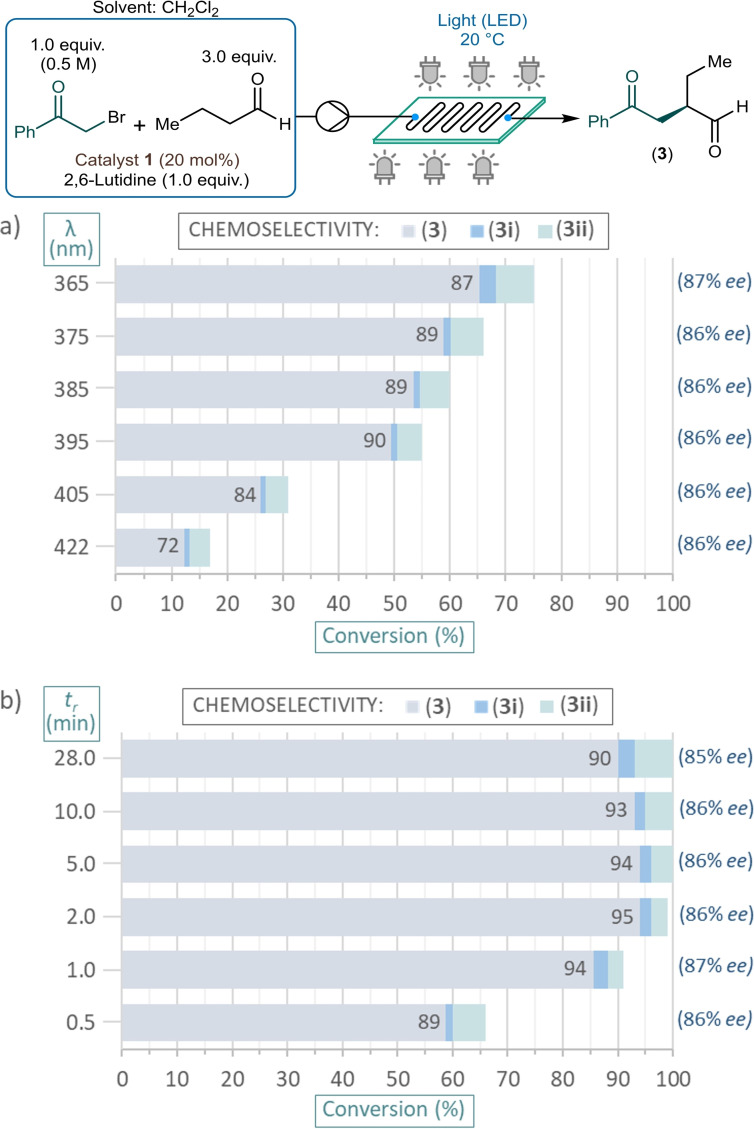
Investigating the effects of various wavelengths and residence times on the asymmetric photochemical alkylation of butyraldehyde with 2‐bromoacetophenone under flow conditions. a) Wavelength screening at 0.5 min residence time. b) Residence time screening at 375 nm wavelength (Conversion and chemoselectivity were determined by HPLC area%, and *ee* values by chiral HPLC. **3i**: acetophenone, **3ii**: unidentified side product(s)).

Initially, the effects of the energy of the irradiating light and different residence times were investigated in the presence of 20 mol% of chiral catalyst **1** and 1 equiv. of 2,6‐lutidine as base. The reactions were performed in CH_2_Cl_2_ as solvent applying a 2‐bromoacetophenone concentration of 0.5 M along with 3 equiv. of butyraldehyde, similarly as in the batch reactions. The reaction mixture was fed continuously into the flow reactor using a single HPLC pump. Various wavelengths were screened at a residence time fixed at 0.5 min (corresponded to 5.540 mL min^−1^ flow rate; Scheme 2a). We were delighted to find that at 365 nm, which corresponded to the highest photon energy investigated, a conversion of 75% was obtained along with 87% chemical selectivity towards the formation of α‐alkylated product **3**. With gradual increase of the wavelength from 365 nm to 395 nm, the chemical selectivity could be increased from 87% to 90%; however, the conversion decreased considerably. Further reduction of the photon energy resulted not only in a more significant decrease in the reaction rate, but also the formation of more side products. On the basis of the data collected, 375 nm delivered an optimum photon energy in terms of conversion and chemical selectivity. On investigating the effects of the residence time while irradiating at 375 nm (Scheme 2b), 2 min was found sufficient to achieve almost quantitative conversion along with an excellent chemical selectivity of 95%, which can be regarded as a significant acceleration compared with the batch data shown in Table 1. At residence times higher than 5 min, some decrease of chemical selectivity occurred, which is possibly due to overirradiation‐caused side product formation. The *ee* of the reactions did not show any significant dependence on the wavelength or on the residence time, being in the region 85–87% in all reactions explored.

Having established the practically useful regions of residence time and the wavelength of the irradiating light, precise fine‐tuning of further reaction conditions was performed (Table 2). Based on the batch data collected earlier (Table 1), MeCN was also explored as solvent for the flow reactions. Unfortunately, despite attaining high conversion and chemical selectivity, the *ee* in MeCN was only 77%, rendering CH_2_Cl_2_ as the solvent of choice for further method development (entries 1 and 2). Diphenylprolinol **2** was also tested as chiral catalyst under flow conditions, but in line with the batch data, yielded lower activity and poorer chemical selectivity than catalyst **1** (entries 3–5). In order to improve process efficiency, attempts were made to reduce catalyst and reagent consumption. To this end, it was possible to reduce the amount of catalyst **1** to 15 mol% without a significant decrease in conversion or in chemical selectivity (entry 6). However, further reduction of the catalyst loading resulted much lower conversions and the promotion of the formation of acetophenone (**3i**) via debromination of the alkylating agent (entries 7 and 8). Gratifyingly, with a minor modification of the reaction conditions, the aldehyde excess could be reduced from 3.0 to 2.25 equiv. while maintaining a quantitative transformation (entries 9 and 10). It was not possible, however, to reduce the amount of the base additive from the previously set 1 equiv. (entry 11). Importantly, we managed to improve the throughput of the process by increasing substrate concentration to 0.625 M (entry 12). Unfortunately, further increase in concentration resulted some visible precipitation in the reaction channels that may lead to clogging during longer runs (entry 13). Finally, in attempts to fine tune the *ee* of the reaction, the effects of the reaction temperature were explored. We were delighted to find that upon decreasing the reactor temperature from 20 °C to 10 °C, *ee* could be improved from 86% to 90% while maintaining a homogeneous reaction mixture (entry 14). Further reduction of the temperature resulted in only marginal changes in *ee* along with some solid formation in the reactor (entry 15).

**Table 2:** Fine‐tuning of the conditions of the continuous flow model reaction.

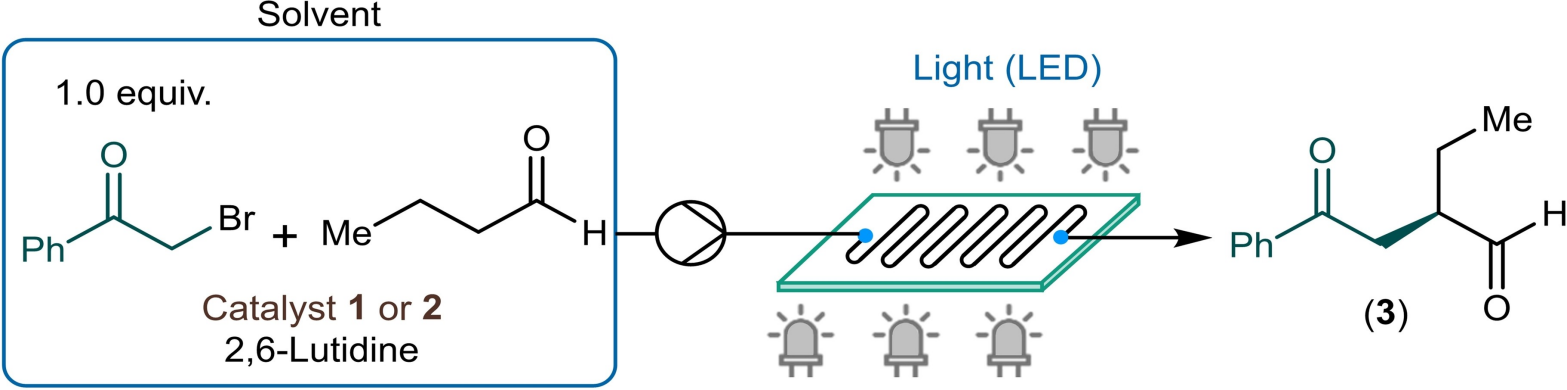														
#	Solvent	Catalyst		t_r_ (min)	λ (nm)	T (°C)	Aldehyd (equiv.)	Base (equiv.)	*c*_*substr*._ (M)	Conv. (%)^[a]^	Select. (%)^[a]^			*ee* (%)^[d]^
		**1** or **2**	(mol%)								(**3**)	(**3i**)^[b]^	(**3ii**)^[c]^	
1	CH_2_Cl_2_	**1**	20	2.0	375	20	3.0	1.0	0.5	99	95	2	3	86
2	MeCN	**1**	20	2.0	375	20	3.0	1.0	0.5	94	94	1	5	77
3	CH_2_Cl_2_	**2**	20	5.0	375	20	3.0	1.0	0.5	72	19	14	67	n.d.
4	CH_2_Cl_2_	**2**	20	28.0	375	20	3.0	1.0	0.5	100	11	27	62	n.d.
5	CH_2_Cl_2_	**2**	20	28.0	395	20	3.0	1.0	0.5	65	45	16	39	n.d.
6	CH_2_Cl_2_	**1**	15	2.0	375	20	3.0	1.0	0.5	96	94	3	3	86
7	CH_2_Cl_2_	**1**	10	2.0	375	20	3.0	1.0	0.5	80	88	7	5	85
8	CH_2_Cl_2_	**1**	5	2.0	375	20	3.0	1.0	0.5	22	46	54	0	n.d.
9	CH_2_Cl_2_	**1**	15	2.0	375	20	2.25	1.0	0.5	95	92	4	4	86
10	CH_2_Cl_2_	**1**	15	5.0	375	20	2.25	1.0	0.5	100	90	4	6	86
11	CH_2_Cl_2_	**1**	20	2.0	375	20	3.0	0.5	0.5	72	88	5	7	86
12	CH_2_Cl_2_	**1**	15	5.0	375	20	2.25	1.0	0.625	100	93	3	4	86
13^[e]^	CH_2_Cl_2_	**1**	15	5.0	375	20	2.25	1.0	0.75	100	95	2	3	87
14	CH_2_Cl_2_	**1**	15	5.0	375	10	2.25	1.0	0.625	100	93	3	4	90
15^[e]^	CH_2_Cl_2_	**1**	15	5.0	375	0	2.25	1.0	0.625	100	93	3	4	91

^[a]^ Determined by HPLC area%. ^[b]^ **3i**: acetophenone. ^[c]^ **3ii**: Unidentified side product(s). ^[d]^ Determined by chiral HPLC. ^[e]^ Cloudy mixture. ^[f]^ Precipitation occurred. ^[g]^ Clear solution.

**Scheme 3:**
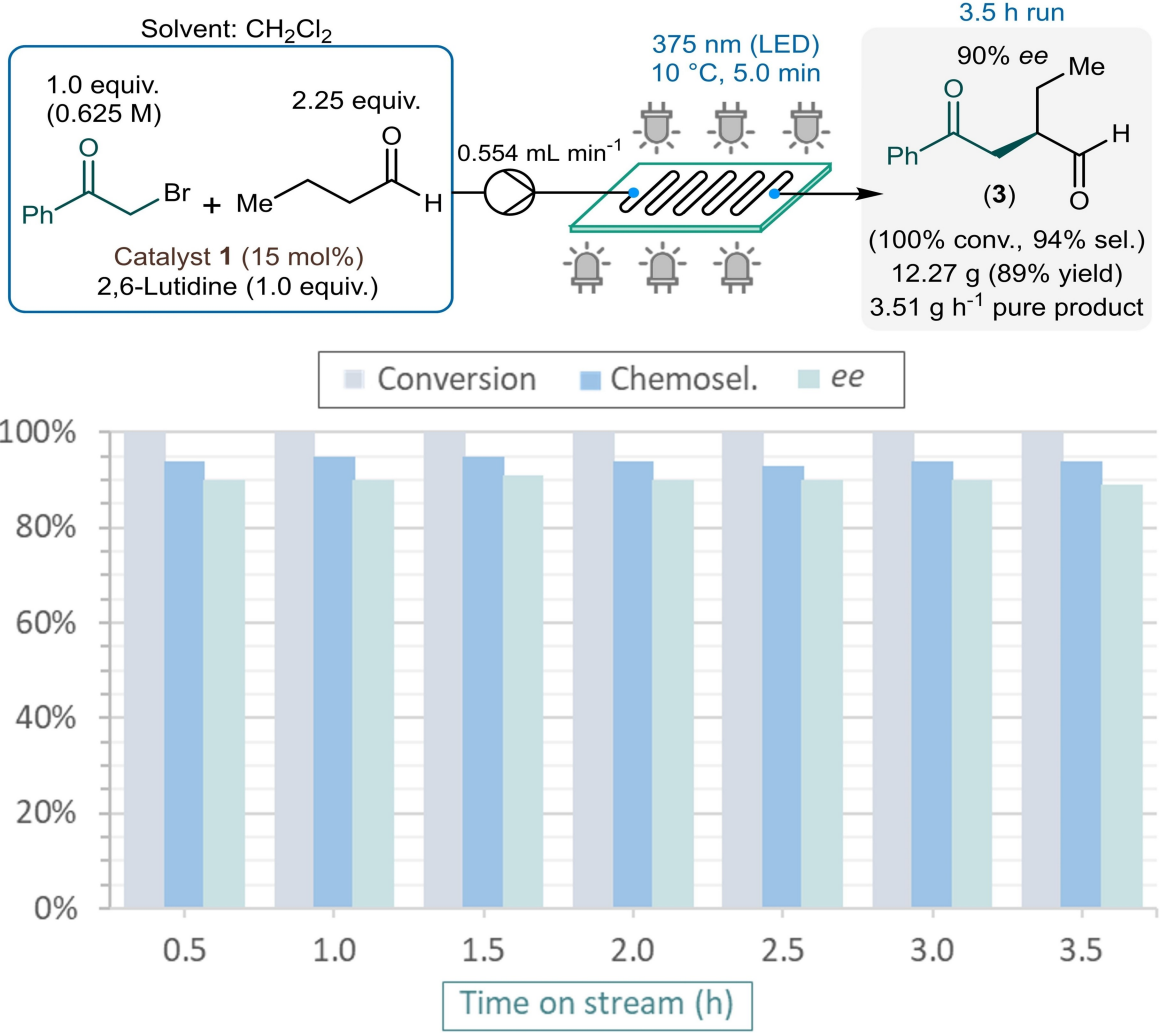
Long run experiment under optimized flow conditions. (Conversion and chemoselectivity were determined by HPLC area%, and *ee* values by chiral HPLC.)

In order to verify the stability and the lab‐scale preparative capabilities of the process, the asymmetric α‐alkylation of butyraldehyde with 2‐bromoacetophenone as alkylating agent was performed as a long‐run under optimized flow conditions (Scheme 3). To this end, a reaction mixture containing 0.625 M of 2‐bromoacetophenone along with 2.25 equiv. of aldehyde, 1.0 equiv. of 2,6‐lutidine and 15 mol% of catalyst **1** was pumped continuously at a flow rate of 0.554 mL min^−1^ (corresponded to 5.0 min residence time), whilst the reaction plate was irradiated at 375 nm and temperature was set to 10 °C. A 3.5 h reaction window was explored under steady state conditions with samples taken every 30 min to determine conversion, chemoselectivity and *ee*. Gratifyingly, the system proved stable, no solid formation or clogging occurred throughout the experiment. In all samples investigated, quantitative conversion was detected along with a chemical selectivity of 93–95% and an *ee* of 90% or 91%. As a result of the experiment, 12.27 g of pure **3** was isolated (89% yield). This correlates to a space‐time‐yield (STY) of 1267 g L^−1^ h^−1^ and a productivity of 3.51 g h^−1^, which is a significant intensification compared to the throughput of earlier batch processes. Importantly, the setup used in this study is directly transferable to production scale utilizing commercial mesofluidic photoreactors.^[22]^

**Scheme 4:**
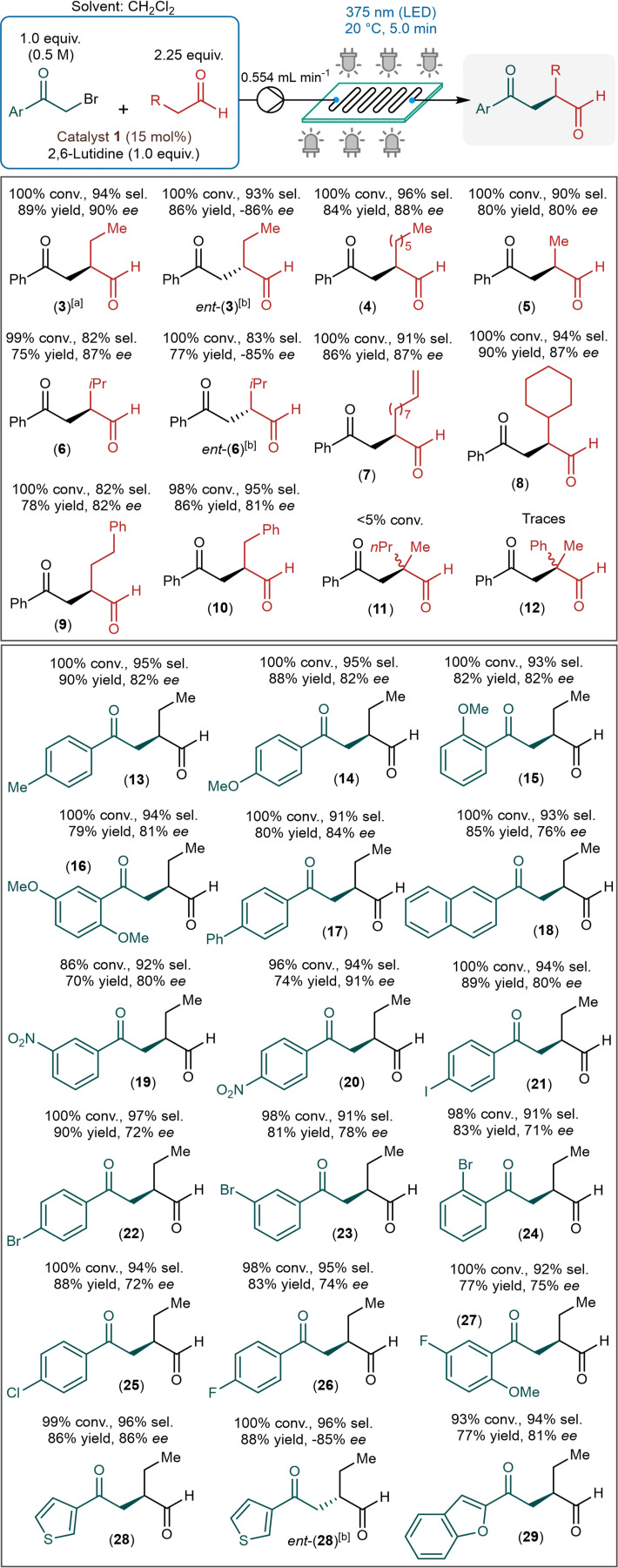
Substrate scope of the continuous flow asymmetric photochemical aldehyde alkylation (Conversion and chemoselectivity were determined by HPLC area%, and *ee* values by chiral HPLC. Yields shown are isolated yields. [a] Reaction conditions: *c*_*substr*._=0.625 M, *T*=10 °C. [b] The opposite catalyst enantiomer, catalyst *ent*‐1 was used).

With the aim to demonstrate the scope and generality of the flow process, reactions of a range of aldehydes and phenacyl bromide derivatives were explored next in the presence of diarylprolinol **1** as chiral organocatalyst (Scheme 4). Most of the reaction conditions were set to the previously optimized values, but in order to minimize the risk of precipitation and clogging in the reaction channels, the concentration of the alkylating agent was reduced to 0.5 M and the reactor temperature was increased to 20 °C.

Various aldehydes were reacted using 2‐bromoacetophenone as alkylating agent. As concerns linear aliphatic aldehydes, octanal and propanal were tested besides the model compound. Both furnished quantitative and highly selective transformations, and the corresponding chiral 1,4‐dicarbonyls (**4** and **5**) were obtained in yields of 84% and 80% along with 88% and 80% *ee*, respectively. Reactions of the β‐branched isovaleraldehyde and cyclohexylacetaldehyde exhibited ≥99% conversion and high chemical selectivity and provided the corresponding alkylated products in 75% and 86% yield, and 87% *ee* in both cases. Undecylenic aldehyde, containing a C−C double bond, was also alkylated quantitatively and selectively. Side reactions of the double bond were not detected, and the desired chiral product (**8**) was isolated with 90% yield and 87% *ee*. The homologous 4‐phenylbutanal and 3‐phenylpropanal were also tolerated well by the flow process, yields were 78% and 86% and *ee*s were 82% and 81%, respectively for compounds **9** and **10**. Reactions of the α‐branched 2‐methylpentanal and 2‐phenylpropanal were also explored, but both furnished very low conversions rendering such aldehydes a current limitation of the process.

The scope of phenacyl bromide derivatives was investigated using butyraldehyde as reaction partner. Excellent results were achieved with a diverse set of methyl‐, methoxy‐ and phenyl‐substituted phenacyl bromides as well as with 2‐naphthacyl bromide. All these reactions were quantitative and highly selective (chemoselectivity ≥91%), and the corresponding alkylated chiral aldehydes (**13**–**18**) were isolated in yields of 79–90% and *ee*s up to 84%. Nitro‐substituted phenacyl bromides furnished the corresponding 1,4‐dicarbonyl substances (**19** and **20**) with good yields and *ee*s up to 91%. In asymmetric α‐alkylations with phenacyl bromides bearing various halogen substituents, the anticipated alkylated products (**21**–**27**) were produced selectively without the dehalogenation of the aromatic ring. In these reactions, conversions were ≥98% regardless of the position of the substituent(s) on the aromatic rings, and yields were between 77% and 90% along with *ee*s in the region of 71–80%. The flow process also proved effective for reactions of sulphur‐ and oxygen‐ containing heterocyclic phenacyl bromide derivatives. 3‐(Bromoacetyl)thiophene and 2‐(bromoacetyl)benzofuran served as effective alkylating agents and furnished the corresponding chiral adducts **28** and **29** in 86% and 77% yield, and 86% and 81% *ee*, respectively. Unfortunately, 3‐(bromoacetyl)pyridine could not be processed due precipitation issues. Besides 2‐bromoacetophenone, 2‐chloroacetophenone was also explored as a potential alkylating agent. To our delight, despite its considerably lower reactivity, 2‐chloroacetophenone still worked reasonably well in reaction with butyraldehyde under flow conditions. By extending the residence time from 5 min to 15 min, but otherwise under unchanged reaction conditions, 55% conversion and 72% chemical selectivity were given along with an *ee* of 86% (Table S2 in the Supporting Information).

By switching from diarylprolinol **1** to its opposite enantiomer (catalyst *ent*‐**1**), which is also available commercially, a facile route is provided for the synthesis of both enantiomers of the valuable α‐alkylated products. In order to verify this, some of the reactions were repeated in the presence of catalyst *ent*‐**1**, but otherwise under identical reaction conditions. As anticipated, the corresponding chiral adducts *ent*‐**3**, *ent*‐**6** and *ent*‐**28** were readily obtained with yields and *ee*s comparable to those achieved with diarylprolinol **1** as chiral catalyst, but with the opposite absolute configuration.

The applicability of the present flow process was directly compared to batch reaction data taken from the relevant literature (see Table S3 in the Supporting Information for details).^[13a]^ As concerns the isolated yields and the enantiomeric purity of the prepared chiral products, the two protocols provided similar results for all six substances (**3**, **17**, **18**, **19**, **20** and **23**) that could directly be compared. However, the flow process employing the commercially available organocatalyst required only 5 min residence time for completion, whereas the batch reactions demanded reaction times up to 103 h.

**Scheme 5:**
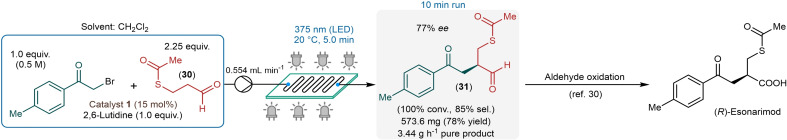
Continuous flow enantioselective synthesis of a key (*R*)‐esonarimod intermediate.

Esonarimod is a propionic acid derivative that was developed originally as an antirheumatic drug.^[24]^ It has a variety of effects on cellular and mediator events in inflammatory processes including the inhibition of inflammatory cytokine production,^[25]^ and it suppresses the development of arthritis in various animal models.^[26]^ Esonarimod has been initially employed clinically as a racemate that was prepared via a multistep procedure relying on a Friedel‐Crafts acylation in the presence of AlCl_3_ and nitrobenzene as key step.[^24^, ^27^] The involvement of hazardous reagents in this synthetic route as well as the low yield obtained were acting as significant obstacles to large scale production. Even though it was demonstrated that there are considerable differences in biological activities of esonarimod enantiomers,^[28]^ no approach has been reported yet for the enantioselective asymmetric synthesis of esonarimod or its advanced intermediates. We speculated that with the suitable aldehyde component in hand, the present flow process may be exploited for the scalable asymmetric synthesis of esonarimod. To test our hypothesis, 3‐(acetylthio)propionaldehyde (**30**) was prepared following a literature procedure (Scheme S1 in the Supporting Information).^[29]^ Then aldehyde **30** was submitted to asymmetric α‐alkylation using commercially available 2‐bromo‐*p*‐methylacetophenone as alkylating agent in the presence of diarylprolinol **1** as chiral catalyst and 2,6‐lutidine as base under the previously optimized reaction conditions (Scheme 5). To our delight, the asymmetric flow process readily furnished an advanced chiral intermediate of esonarimod (**31**) with an *ee* of 77%. In a 10 min run, 573.6 mg (78% yield) of the enantioenriched target compound was isolated which correlates to a productivity of 3.44 g h^−1^. Importantly, (*R*)‐esonarimod can easily be attained from compound **31** by means of simple aldehyde oxidation,^[30]^ and by using diarylprolinol *ent*‐1 as chiral catalyst of the asymmetric key step, (*S*)‐esonarimod can also be obtained.

## Conclusion

The merger of visible light‐driven enantioselective organocatalysis and flow chemistry has been achieved and harnessed for the asymmetric α‐alkylation of aldehydes. A commercially available diarylprolinol trimethylsilyl ether‐type organocatalyst has been utilized to enable EDA complex‐directed exogenous photosensitizer‐free enantioselective alkylations using phenacyl bromide derivates as alkylating agents. The effects of reaction conditions were explored carefully in order to achieve high yielding and selective transformations, whilst minimizing reagent and catalyst consumption and maximizing throughput. The preparative capabilities and robustness of the process were verified through a long‐run experiment yielding multigrams per hour of an enantioenriched model substrate utilizing a commercial lab‐scale photoreactor. By virtue of the precisely controlled continuous flow reaction environment, an accelerated protocol has been developed that enabled the enantioselective α‐alkylation of a diverse set of aldehydes with a broad selection of phenacyl bromide derivatives as alkylating agents. In particular, the present flow process granted entry into the asymmetric formal synthesis of the antirheumatic esonarimod. Unlike in earlier batch approaches where long reaction times (up to 103 h) were demanded, an irradiation time as low as 5 min was sufficient to achieve quantitative transformations in most of the flow reactions explored, whilst the yield and enantiomeric purity of the prepared substances compared well with the available literature data. The improvement in productivity in combination with a smart dimensioning‐directed scale‐up strategy ensure facile transferability to manufacturing scales by using commercial mesofluidic photoreactors.

## Experimental Section

### Preliminary Batch Experiments

2 mL of a solution containing catalyst **1** or **2** (0.2 mmol, 20 mol%, 119.50 mg or 65.11 mg, respectively), 2‐bromoacetophenone (1.0 mmol, 199.05 mg), butyraldehyde (3.0 mmol, 270.4 μL) and 2,6‐lutidine (1.0 mmol, 115.8 μL) in MTBE, 2MeTHF, MeOH, EtOH, *i*PrOH, MeCN, EtOAc, acetone, toluene or CH_2_Cl_2_ as solvent was prepared at room temperature in a 4 mL microwave vial equipped with a magnetic stirring bar. The vial was closed with a crimp cap, and the mixture was degassed by sparging with a balloon of argon. The reactions were performed at room temperature under argon atmosphere by irradiation using a 372 nm LED (50 W input power) at a distance of 5 cm. 20 μL aliquots of the crude material were taken, the samples were diluted with 1 mL of CH_3_CN and was analyzed by analytical HPLC to determine conversion and chemical selectivity. After completion of the reaction, the *ee* was determined using chiral HPLC. For this, 20 μL aliquots of the crude samples were diluted directly with 1 mL of heptane/*i*PrOH 1:1, and the samples were analyzed right after the reactions to avoid incidental racemization.

### Continuous Flow Reactor Setup

Flow reactions were performed in a Corning Advanced‐Flow Lab Photo Reactor (Figure S1 in the Supporting Information). As central element, the instrument relied on a compact glass fluidic module (G1 “low flow” fluidic module, 155×125×8 mm size, 0.3 mm channel depth, 2.77 mL internal volume) embedded within a high‐capacity heat exchanger (20 mL volume). The system comprised two LED panels each equipped with 20 LEDs of 6 different wavelengths (120 LEDs in total) and a heat exchanger set to 15 °C. The LED panels were mounted 40 mm from the center of the reaction plate on both sides of the fluidic module. The intensity and wavelength of the LEDs were controlled externally using a web‐based interface running on a tablet and connected wirelessly to the photoreactor. (Light intensity was kept at 100% in all experiments.) Two separate panels were used for various wavelengths: ‘Panel 1’ (365, 385, 405, 485, 610 nm and “4000 K” white light) and ‘Panel 2’ (340, 375, 395, 422, 450, 540 nm). Thermal regulation of the panels was performed using a Huber Minichiller 280 filled with 30% ethylene glycol in water. Thermal regulation of the glass fluidic module was carried out using a Huber Ministat 230 filled with silicon oil (−20 °C to 195 °C). The system contained two built‐in HPLC pumps (FLOM UI 22‐110DC HPLC pump; flow range: 0.01–10 mL min^−1^) to introduce liquid feeds and was pressurized at 3 bar by using an adjustable backpressure regulator (BPR) from Zaiput. Connection between the pump(s), fluidic module input and output was achieved using 1/8” OD perfluoroalkoxy alkane (PFA) tubing (Swagelok) and metal‐free connectors (Swagelok MS‐GC‐2 swaging system). For all other connections 1/16” OD PFA tubing was used together with polyether ether ketone (PEEK) fittings. Reagent feeds were either streamed directly or by using a 6‐way injection valve (Upchurch) and sample loops (PFA tubing; 1/16” OD, 0.80 mm ID).

### Investigation of the Reaction Scope

The flow reactor was turned on (LEDs, pump and thermostats) and the following parameters were set: 0.554 mL min^−1^ flow rate (corresponds to 5.0 min residence time), 375 nm wavelength and 20 °C reactor temperature. The system was equilibrated for approx. 15 min while pumping CH_2_Cl_2_ using one of the built‐in HPLC pumps. At the same time, 10 mL solution containing 0.5 M of the corresponding phenacyl bromide derivative (1.0 equiv.), 2.25 equiv. of the corresponding aldehyde, 1.0 equiv. of 2,6‐lutidine and 15 mol% of catalyst **1** (or its opposite enantiomer, catalyst *ent*‐**1**) in CH_2_Cl_2_ as solvent was prepared in a volumetric flask. The flask was sealed with a septum and parafilm and the solution was sparged with argon for 5 min using a balloon, then loaded into a 9.0‐mL injection loop. After the system has stabilized, the reaction mixture was injected from the sample loop. After reaching steady state, the solution exiting the reactor was collected continuously for 4–8 min. The collected crude material was analyzed directly after the experiment. A 20 μL aliquot was diluted with 1 mL of CH_3_CN to determine conversion and chemical selectivity by means of analytical HPLC, and another 20 μL portion was diluted with 1 mL of heptane/*i*PrOH 1:1 to determine the *ee* by chiral HPLC analysis. After evaporation, the collected material was purified by flash chromatography (cyclohexane→cyclohexane/EtOAc 8:2 as eluent; detection at 254 nm) and was characterized by ^1^H and ^13^C NMR spectroscopy, HRMS and by optical rotation measurement.

Further details on instrumentation, reaction setups as well as general experimental procedures can be found in the Supporting Information.

## Supporting information

As a service to our authors and readers, this journal provides supporting information supplied by the authors. Such materials are peer reviewed and may be re‐organized for online delivery, but are not copy‐edited or typeset. Technical support issues arising from supporting information (other than missing files) should be addressed to the authors.

Supporting Information
