# Supplementary material for: Merger of Visible Light‐Driven Chiral Organocatalysis and Continuous Flow Chemistry: An Accelerated and Scalable Access into Enantioselective α‐Alkylation of Aldehydes
Source: Adv Synth Catal. 2023 May 16;365(10):1660–70. doi: 10.1002/adsc.202300289 (PMC10952295; doi:10.1002/adsc.202300289)
Supplement: Supplementary file 1 — Supporting Information [file ADSC-365-1660-s001.pdf]

Advanced Synthesis & Catalysis

Supporting Information

Merger of Visible Light-Driven Chiral Organocatalysis and Continuous Flow Chemistry: An Accelerated and Scalable Access into Enantioselective α-Alkylation of Aldehydes

Márk Molnár, C. Oliver Kappe,* and Sándor B. Ötvös*© 2023 The Authors. Advanced Synthesis & Catalysis published by Wiley-VCH GmbH. This is an open access article under the terms of the Creative Commons Attribution License, which permits use, distribution and reproduction in any medium, provided the original work is properly cited.

SUPPORTING INFORMATION

for

Merger of Visible Light-Driven Chiral Organocatalysis and Continuous Flow Chemistry: An Accelerated and Scalable Access into Enantioselective α-Alkylation of Aldehydes

Márk Molnár,[a,b] C. Oliver Kappe*,[a,c] and Sándor B. Ötvös*,[a,c]

[a]Institute of Chemistry, University of Graz, NAWI Graz, Heinrichstrasse 28, A-8010 Graz, Austria
[b]Servier Research Institute of Medicinal Chemistry, Záhony u. 7, 1031 Budapest, Hungary
[c]Center for Continuous Flow Synthesis and Processing (CC FLOW), Research Center Pharmaceutical Engineering GmbH (RCPE), Inffeldgasse 13, A-8010 Graz, Austria

*E-mail: oliver.kappe@uni-graz.at (COK); sandor.oetvoes@uni-graz.at (SBÖ)

Table of Contents

1. General information S2
2. Description of preliminary batch experiments S2
3. Investigation of the enantiomeric stability of compound **3** S2
4. Description of continuous flow experiments S3
  4.1. Continuous flow reactor setup S3
  4.2. Parameter optimization S4
  4.3. Long run S4
  4.4. Investigation of the reaction scope S5
  4.5. α-Alkylation with 2-chloroacetophenone S5
  4.6. Comparison with batch data S6
  4.7. Synthesis of a key (*R*)-esonarimod intermediate S6
5. Preparation of racemic reference samples S7
6. Characterization data S8
7. Collection of NMR spectra and HPLC chromatograms S18
8. References S70

1. General information

All solvents and chemicals were obtained from typical commercial vendors and were used as received, without any further purification.

Column chromatographic purification was performed by using a Biotage Isolera automated flash chromatography system with cartridges packed with KP-SIL, 60 Å (32–63 µm particle size). Analytical thin-layer chromatography (TLC) was carried out using Merck silica gel 60 GF254 plates. Compounds were visualized by means of UV or by using $KMnO_4$.

^{1}H- and ^{13}C-NMR spectra were recorded on a Bruker Avance III 300 MHz instrument at room temperature, in $CDCl_3$ as solvent, at 300 MHz and 75 MHz, respectively. Chemical shifts (δ) are reported in ppm using TMS as internal standard. Coupling constants are given in Hz units.

Analytical HPLC measurements were carried out on a C18 reversed-phase column (150 × 4.6 mm, particle size 5 mm) at 37 °C using mobile phases A [H_2O/CH_3CN 90:10 (v/v) + 0.1% TFA] and B (CH_3CN + 0.1% TFA) at a flow rate of 1.5 mL min^{-1}. The gradient applied was as follows: linear increase from 30% solution B to 100% B in 8 min, hold at 100% solution B for 2 min.

The *ee* of the compounds was determined by using a Shimadzu HPLC system (DGU-14A degasser, SCL-10A VP system controller, SPD-10 UV-VIS detector, LC-20AT pumps) with isocratic mixtures of hexane and *i*PrOH as eluent. Chromatographic conditions are represented in section 6.

Optical rotation was measured in $CHCl_3$ (HPLC-grade) at 25 °C against the sodium D-line (λ= 589 nm) on a Perkin Elmer Polarimeter 341 using a 10-cm pathlength cell.

High resolution mass spectra were recorded on an Agilent 6230 TOF LC/MS (G6230B) by flow injections on an Agilent 1260 Infinity Series HPLC (HiP Degasser G4225A, Binary Pump G1312B, ALS Autosampler G1329B, TCC Column thermostat G1316A, DAD Detector G4212B).

2. Description of preliminary batch experiments

2 mL of a solution containing catalyst **1** or **2** (0.2 mmol, 20 mol%, 119.50 mg or 65.11 mg, respectively), 2-bromoacetophenone (1.0 mmol, 199.05 mg), butyraldehyde (3.0 mmol, 270.4 µL) and 2,6-lutidine (1.0 mmol, 115.8 µL) in MTBE, 2MeTHF, MeOH, EtOH, *i*PrOH, MeCN, EtOAc, acetone, toluene or CH_2Cl_2 as solvent was prepared at room temperature in a 4 mL microwave vial equipped with a magnetic stirring bar. The vial was closed with a crimp cap, and the mixture was degassed by sparging with a balloon of argon. The reactions were performed at room temperature under argon atmosphere by irradiation using a 372 nm LED (50 W input power) at a distance of 5 cm. 20 µL aliquots of the crude material were taken, the samples were diluted with 1 mL of CH_3CN and was analyzed by analytical HPLC to determine conversion and chemical selectivity. After completion of the reaction, the *ee* was determined using chiral HPLC. For this, 20 µL aliquots of the crude samples were diluted directly with 1 mL of heptane/*i*PrOH 1:1, and the samples were analyzed right after the reactions to avoid incidental racemization.

3. Investigation of the enantiomeric stability of compound 3

The asymmetric photochemical alkylation yields a configurationally labile α-stereocenter that may racemize *via* enolization. The enantiomeric stability of compound **3** was therefore examined in a control experiment.

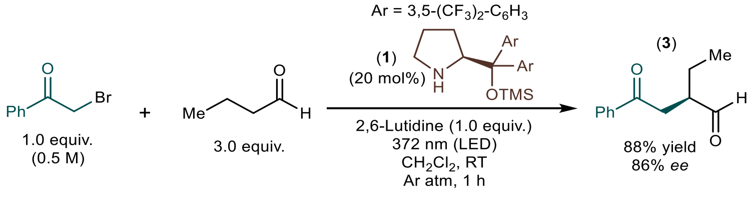

Scheme S1. Batch synthesis of compound **3**.

Compound **3** was prepared by reaction of butyraldehyde with 2-bromoacetophenone in the presence of organocatalyst **1**, 2,6-lutidine and CH_2Cl_2 as solvent following the batch procedure described in section 2.

After evaporation, crude **3** was purified by flash chromatography (cyclohexane → cyclohexane/EtOAc 8:2 as eluent; detection at 254 nm).

The *ee* of compound **3** was then investigated at room temperature as a function of time under different conditions (*A*, *B*, *C* and *D*).

A: pure **3** dissolved in CH_2Cl_2 (0.5 M)
B: pure **3** dissolved in CH_2Cl_2 (0.5 M) + 2,6-lutidine (1.0 equiv.)
C: pure **3** dissolved in CH_2Cl_2 (0.5 M) + organocatalyst **1** (0.2 equiv.)
D: pure **3** dissolved in CH_2Cl_2 (0.5 M) + 2,6-lutidine (1.0 equiv.) + organocatalyst **1** (0.2 equiv.)

Table S1. Investigation of the enantiomeric stability of **3**.

		***ee* (%)**[a]					
		**t_0**[b]	**1 h**	**3 h**	**16 h**	**4 days**	**8 days**
**Conditions**	*A*	86	86	86	85	83	n.d.[c]
	B	86	86	86	86	84	82
	C	86	86	86	84	71	41
	D	86	84	84	84	83	74

[a]Determined by chiral HPLC. [b]Represents the *ee* measured right after asymmetric alkylation. [c]Carboxylic acid formation.

This data indicates that the racemization of pure **3** is detectable at room temperature. In the presence of the chiral organocatalyst, samples were enantiomeric stable until at least 3 h, but later notable racemization occurred. Racemization was also accelerated by the presence of the base.

4. Description of continuous flow experiments

4.1. Continuous flow reactor setup

Flow reactions were performed in a Corning Advanced-Flow Lab Photo Reactor (Figure S1).

As central element, the instrument relied on a compact glass fluidic module (G1 “low flow” fluidic module, 155 × 125 × 8 mm size, 0.3 mm channel depth, 2.77 mL internal volume) embedded within a high-capacity heat exchanger (20 mL volume). The system comprised two LED panels each equipped with 20 LEDs of 6 different wavelengths (120 LEDs in total) and a heat exchanger set to 15 °C. The LED panels were mounted 40 mm from the center of the reaction plate on both sides of the fluidic module. The intensity and wavelength of the LEDs were controlled externally using a web-based interface running on a tablet and connected wirelessly to the photoreactor. (Light intensity was kept at 100% in all experiments.)

Two separate panels were used for various wavelengths: ‘Panel 1’ (365, 385, 405, 485, 610 nm and “4000K” white light) and ‘Panel 2’ (340, 375, 395, 422, 450, 540 nm). The emission spectra of the LEDs on ‘Panel 1’ and ‘Panel 2’ can be found in the literature.[S1] Thermal regulation of the panels was performed using a Huber Minichiller 280 filled with 30% ethylene glycol in water. Thermal regulation of the glass fluidic module was carried out using a Huber Ministat 230 filled with silicon oil (–20 °C to 195 °C).

The system contained two built-in HPLC pumps (FLOM UI 22-110DC HPLC pump; flow range: 0.01–10 mL min^{-1}; wetted-parts: PTFE, PCTFE, FFKM and ruby) to introduce liquid feeds and was pressurized at 3 bar by using an adjustable backpressure regulator (BPR) from Zaiput.

Connection between the pump(s), fluidic module input and output was achieved using 1/8” OD perfluoroalkoxy alkane (PFA) tubing (Swagelok) and metal-free connectors (Swagelok MS-GC-2 swaging system). For all other connections 1/16” OD PFA tubing was used together with polyether ether ketone (PEEK) fittings. Reagent feeds were either streamed directly or by using a 6-way injection valve (Upchurch) and sample loops (PFA tubing; 1/16” OD, 0.80 mm ID).

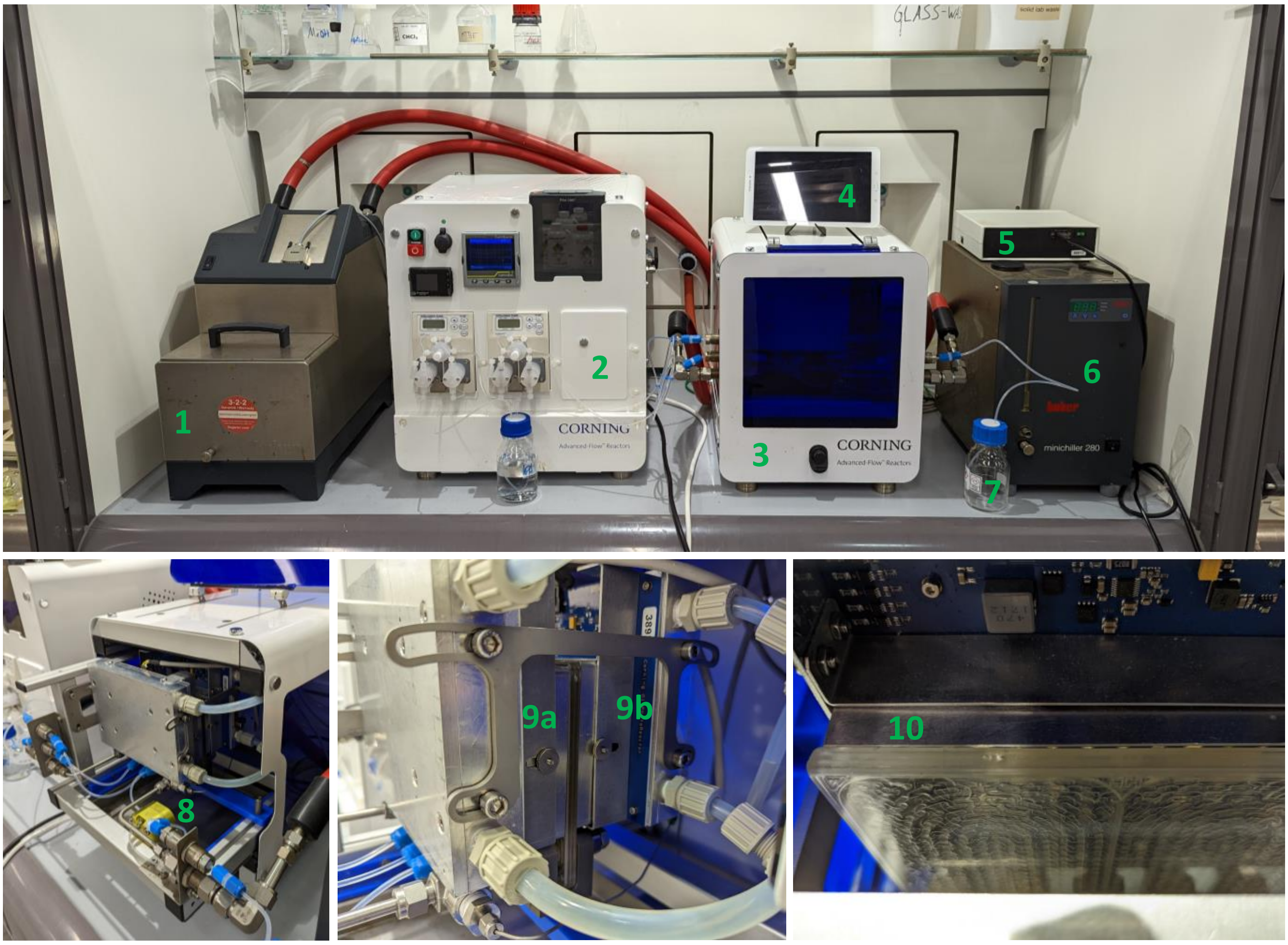

Figure S1. Photograph of the photoreactor setup. 1) Temperature control for the reaction plate, 2) control module containing pumps, mass flow controller, Huber controller and data logger, 3) fluidic module housing with tinted plastic panels for light containment, 4) tablet for controlling intensity and wavelength of the LEDs, 5) wireless receiver for LED control, 6) temperature control for LED panels, 7) reactor outlet, 8) adjustable BPR, 9a/b) LED panels, 10) glass fluidic module.

4.2. Parameter optimization

The reactor was turned on (LEDs, pump and thermostats), and the system was equilibrated for approx. 15 min while pumping CH_2Cl_2, MeCN or *i*PrOH using one of the built-in HPLC pumps. At the same time, 5 mL solution containing 0.5 M, 0.625 M or 0.75 M of 2-bromoacetophenone (1.0 equiv.), 2.25 equiv. or 3.0 equiv. of butyraldehyde, 0.5 equiv. or 1.0 equiv. of 2,6-lutidine and 5, 10, 15 or 20 mol% of the corresponding chiral catalyst (**1** or **2**) in CH_2Cl_2, MeCN or *i*PrOH as solvent was prepared in a volumetric flask. The flask was sealed with a septum and parafilm and the solution was sparged with argon for 5 min using a balloon, then loaded into a 4.8-mL injection loop. After the system has stabilized, the reaction mixture was injected from the sample loop. The central fraction (approx. 0.5 mL) of the injected sample was collected into a vial under steady state conditions and was analyzed directly after the experiment. A 20 µL aliquot of the collected material was diluted with 1 mL of CH_3CN to determine conversion and chemical selectivity by means of analytical HPLC, and another 20 µL portion was diluted with 1 mL of heptane/*i*PrOH 1:1 to determine the *ee* by chiral HPLC analysis.

During parameter optimization, flow rates of 5.540 mL min^{-1}, 2.770 mL min^{-1}, 1.390 mL min^{-1}, 0.554 mL min^{-1}, 0.277 mL min^{-1} and 0.100 mL min^{-1} (corresponding to 0.5 min, 1.0 min, 2.0 min, 5.0 min, 10.0 min and 28.0 residence time, respectively), wavelengths of 422 nm, 405 nm, 395 nm, 385 nm, 375 nm and 365 nm, and temperatures of 20 °C, 10 °C and 0 °C were explored.

4.3. Long run

The reactor was turned on (LEDs, pump and thermostats) and the following parameters were set: 0.554 mL min^{-1} flow rate (corresponds to 5.0 min residence time), 375 nm wavelength and 10 °C reactor temperature. The system was equilibrated for approx. 15 min while pumping CH_2Cl_2 using one of the built-in HPLC pumps. At the same

time, 120 mL solution containing 0.625 M of 2-bromoacetophenone (1.0 equiv.), 2.25 equiv. of butyraldehyde, 1.0 equiv. of 2,6-lutidine and 15 mol% of the corresponding chiral catalyst **1** in CH_2Cl_2 as solvent was prepared. The solution was sparged with argon for 15 min using a balloon. After the system has stabilized, the reaction mixture was pumped directly. After reaching steady state, the solution exiting the reactor was collected continuously for 3.5 h. The collected crude material was analyzed directly after the experiment. A 20 µL aliquot was diluted with 1 mL of CH_3CN to determine conversion and chemical selectivity by means of analytical HPLC, and another 20 µL portion was diluted with 1 mL of heptane/*i*PrOH 1:1 to determine the *ee* by chiral HPLC analysis. After evaporation, crude **3** was purified by flash chromatography (cyclohexane → cyclohexane/EtOAc 8:2 as eluent; detection at 254 nm) and was characterized by ^{1}H and ^{13}C NMR spectroscopy, HRMS and by optical rotation measurement.

4.4. Investigation of the reaction scope

The reactor was turned on (LEDs, pump and thermostats) and the following parameters were set: 0.554 mL min^{-1} flow rate (corresponds to 5.0 min residence time), 375 nm wavelength and 20 °C reactor temperature. The system was equilibrated for approx. 15 min while pumping CH_2Cl_2 using one of the built-in HPLC pumps. At the same time, 10 mL solution containing 0.5 M of the corresponding phenacyl bromide derivative (1.0 equiv.), 2.25 equiv. of the corresponding aldehyde, 1.0 equiv. of 2,6-lutidine and 15 mol% of catalyst **1** (or its opposite enantiomer, (*R*)-α,α-bis[3,5-bis(trifluoromethyl)phenyl]-2-pyrrolidinemethanol trimethylsilyl ether, catalyst *ent*-**1**) in CH_2Cl_2 as solvent was prepared in a volumetric flask. The flask was sealed with a septum and parafilm and the solution was sparged with argon for 5 min using a balloon, then loaded into a 9.0-mL injection loop. After the system has stabilized, the reaction mixture was injected from the sample loop. After reaching steady state, the solution exiting the reactor was collected continuously for 4–8 min. The collected crude material was analyzed directly after the experiment. A 20 µL aliquot was diluted with 1 mL of CH_3CN to determine conversion and chemical selectivity by means of analytical HPLC, and another 20 µL portion was diluted with 1 mL of heptane/*i*PrOH 1:1 to determine the *ee* by chiral HPLC analysis. After evaporation, the collected material was purified by flash chromatography (cyclohexane → cyclohexane/EtOAc 8:2 as eluent; detection at 254 nm) and was characterized by ^{1}H and ^{13}C NMR spectroscopy, HRMS and by optical rotation measurement.

4.5. α-Alkylation with 2-chloroacetophenone

The reactor was turned on (LEDs, pump and thermostats) and the following parameters were set: 0.554 mL min^{-1} or 0.185 mL min^{-1} flow rate (corresponds to 5.0 min or 15.0 min residence time), 375 nm wavelength and 10 °C reactor temperature. The system was equilibrated for approx. 15 min while pumping CH_2Cl_2 using one of the built-in HPLC pumps. At the same time, 5 mL solution containing 0.625 M of 2-chloroacetophenone (1.0 equiv.), 2.25 equiv. or 3.75 equiv. of butyraldehyde, 1.0 equiv. of 2,6-lutidine and 15 or 25 mol% of chiral catalyst **1** in CH_2Cl_2 as solvent was prepared in a volumetric flask. The flask was sealed with a septum and parafilm and the solution was sparged with argon for 5 min using a balloon, then loaded into a 4.8-mL injection loop. After the system has stabilized, the reaction mixture was injected from the sample loop. The central fraction (approx. 0.5 mL) of the injected sample was collected into a vial under steady state conditions and was analyzed directly after the experiment. A 20 µL aliquot of the collected material was diluted with 1 mL of CH_3CN to determine conversion and chemical selectivity by means of analytical HPLC, and another 20 µL portion was diluted with 1 mL of heptane/*i*PrOH 1:1 to determine the *ee* by chiral HPLC analysis.

Table S2. Investigation of α-alkylations with 2-chloroacetophenone.

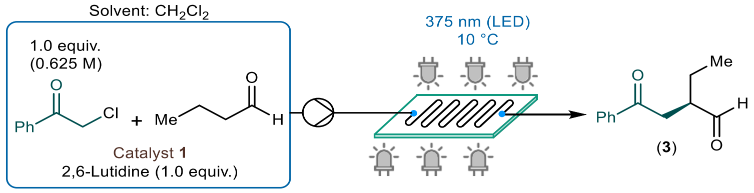

#	Flow rate (mL min^{-1})	t_r (min)	Catalyst (mol%)	Aldehyde (equiv.)	Conv. (%)[a]	Select. (%)[a]			*ee* (%)[d]
						(3)	(3i)[b]	(3ii)[c]	
1	0.554	5.0	15	2.25	34	88	6	6	85
2	0.185	15.0	15	2.25	55	72	9	19	86
3[e]	0.185	15.0	25	3.75	n.d.	n.d.	n.d.	n.d.	n.d.

[a]Determined by HPLC area%. [b]**3i**: acetophenone. [c]**3ii**: unidentified side product(s). [d]Determined by chiral HPLC. [e]Clogging.

4.6. Comparison with batch data

The results shown in Fig. 4 in the manuscript were directly compared with batch reaction data taken from the relevant literature (ref. 13a in the manuscript). In the batch reference, conversion and chemical selectivity data were not disclosed.

Table S3. Comparison of the applicability of the present flow process with literature batch data.

#	Product	Process	Chiral organocatalyst	Time	Conv. (%)	Select. (%)	Yield (%)	*ee* (%)
1	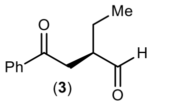 (**3**)	Literature batch[a]	In-house made perhydroindole (20 mol%)	72 h reaction time	n.d.	n.d.	89	94
		This work, flow[b]	Readily available catalyst **1** (15 mol%)	5 min residence time	100	94	89	90
2	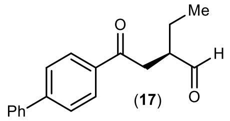 (**17**)	Literature batch[a]	In-house made perhydroindole (20 mol%)	103 h reaction time	n.d.	n.d.	94	86
		This work, flow[c]	Readily available catalyst **1** (15 mol%)	5 min residence time	100	91	80	84
3	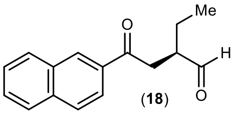 (**18**)	Literature batch[a]	In-house made perhydroindole (20 mol%)	72 h reaction time	n.d.	n.d.	70	86
		This work, flow[c]	Readily available catalyst **1** (15 mol%)	5 min residence time	100	93	85	76
4	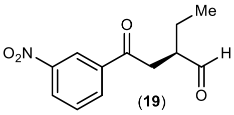 (**19**)	Literature batch[a]	In-house made perhydroindole (20 mol%)	71 h reaction time	n.d.	n.d.	86	86
		This work, flow[c]	Readily available catalyst **1** (15 mol%)	5 min residence time	86	92	70	80
5	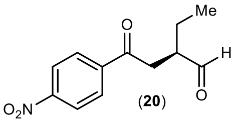 (**20**)	Literature batch[a]	In-house made perhydroindole (20 mol%)	68 h reaction time	n.d.	n.d.	70	83
		This work, flow[c]	Readily available catalyst **1** (15 mol%)	5 min residence time	96	94	74	91
6	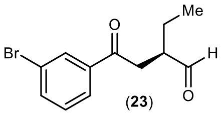 (**23**)	Literature batch[a]	In-house made perhydroindole (20 mol%)	74 h reaction time	n.d.	n.d.	96	87
		This work, flow[c]	Readily available catalyst **1** (15 mol%)	5 min residence time	98	91	81	78

[a]Reaction conditions: 1 equiv. (0.5 M) alkylating agent, 3.0 equiv. butyraldehyde, 1.0 equiv. 2,6-lutidine, MTBE as solvent, *hv* (23 W, CFL), 25 °C. [b]1 equiv. (0.625 M) alkylating agent, 2.25 equiv. butyraldehyde, 1.0 equiv. 2,6-lutidine, CH_2Cl_2 as solvent, *hv* (375 nm, LED), 10 °C. [c]1 equiv. (0.5 M) alkylating agent, 2.25 equiv. butyraldehyde, 1.0 equiv. 2,6-lutidine, CH_2Cl_2 as solvent, *hv* (375 nm, LED), 20 °C.

4.7. Synthesis of a key (R)-esonarimod intermediate

Preparation of aldehyde **30**:

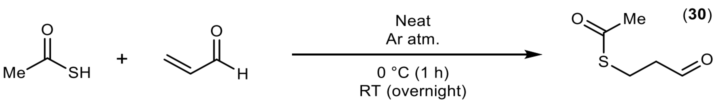

Scheme S1. Synthesis of **30**.

Compound **30** was prepared according to a procedure reported in the literature.[S2]

Acrolein (35.7 mmol; 2.0 g) was added dropwise to thioacetic acid (40.4 mmol; 2.85 mL) at 0°C under argon atmosphere (extremely exothermic). The mixture was stirred at 0°C for 1 h, then it was stirred at room temperature overnight. The excess thioacetic acid was removed by evaporation, and the crude product was purified by flash chromatography (petrolether → petrolether/EtOAc 9:1 as eluent; detection at 254 nm) to give 4.11 g (87% yield) of pure **30** as a colorless liquid. NMR data of the isolated compound matched the reported literature.[S2]

Continuous flow synthesis of **31**:

The reactor was turned on (LEDs, pump and thermostats) and the following parameters were set: 0.554 mL min^{-1} flow rate (corresponds to 5 min residence time), 375 nm wavelength and 20 °C reactor temperature. The system was equilibrated for approx. 15 min while pumping CH_2Cl_2 using one of the built-in HPLC pumps. At the same time, 10 mL solution containing 0.5 M of 2-bromo-*p*-methylacetophenone (1.0 equiv.), 2.25 equiv. of aldehyde **30**, 1.0 equiv. of 2,6-lutidine and 15 mol% of catalyst **1** in CH_2Cl_2 as solvent was prepared in a volumetric flask. The flask was sealed with a septum and parafilm and the solution was sparged with argon for 5 min using a balloon, then loaded into a 9.0-mL injection loop. After the system has stabilized, the reaction mixture was injected from the sample loop. After reaching steady state, the solution exiting the reactor was collected continuously for 10 min. The collected crude material was analyzed directly after the experiment. A 20 µL aliquot was diluted with 1 mL of CH_3CN to determine conversion and chemical selectivity by means of analytical HPLC, and another 20 µL portion was diluted with 1 mL of heptane/*i*PrOH 1:1 to determine the *ee* by chiral HPLC analysis. After evaporation, crude **31** was purified by flash chromatography (cyclohexane → cyclohexane/EtOAc 8:2 as eluent; detection at 254 nm) and was characterized by ^{1}H and ^{13}C NMR spectroscopy, HRMS and by optical rotation measurement.

5. Preparation of racemic reference samples

Racemic reference samples were prepared by using either an equimolar mixture of catalyst **1** together with its opposite enantiomer (catalyst *ent*-**1**), or by using a catalytic amount of morpholine according to the following procedure.

2 mL of a solution containing the racemic catalyst mixture (0.2 mmol, 20 mol% in total) or morpholine (0.3 mmol, 30 mol%, 26.0 µL) the corresponding phenacyl bromide derivative (1.0 mmol), the corresponding aldehyde (3.0 mmol) and 2,6-lutidine (1.0 mmol, 115.8 µL) in CH_2Cl_2 as solvent was prepared at room temperature in a 4 mL microwave vial equipped with a magnetic stirring bar. The vial was closed with a crimp cap, and the mixture was degassed by sparging with a balloon of argon. The reactions were performed at room temperature under argon atmosphere by irradiation using a 372 nm LED (50 W input power) at a distance of 5 cm. 20 µL aliquots of the crude material were taken, the samples were diluted with 1 mL of CH_3CN and was analyzed by analytical HPLC to determine conversion and chemical selectivity. After completion of the reaction, the samples were evaporated, and the collected material was purified by flash chromatography (cyclohexane →cyclohexane/EtOAc 8:2 as eluent; detection at 254 nm).

6. Characterization data

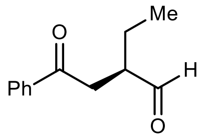

(*R*)-2-Ethyl-4-oxo-4-phenylbutanal (**3**)

Following the flow procedure (long run) described in section 4.3, 12.27 g (89% yield) of the title compound was obtained as yellow oil.

HRMS (ESI) calculated for $C_{12}H_{15}O_2$ (M+H): 191.1072, found: 191.1071.

The *ee* was determined by HPLC using a Chiralpak® IC column (heptane–*i*PrOH 90/10, 1.5 mL min^{-1}, 210 nm, 25 °C): t_{minor}= 10.071 min, t_{major}= 13.151 min, *ee*= 90%.

$[\alpha]_D^{25}$= +40.5 (c= 1.00, $CHCl_3$) {lit.[S3] $[\alpha]_D^{27}$= +33.3 (c= 1.00, $CHCl_3$)}.

^{1}H ($CDCl_3$, 300 MHz): δ 9.85 (s, 1H), 8.03-7.96 (m, 2H), 7.63-7.56 (m, 1H), 7.53-7.45 (m, 2H), 3.56-3.44 (m, 1H), 3.14-2.99 (m, 2H), 1.95-1.79 (m, 1H), 1.73-1.58 (m, 1H), 1.03 (t, *J* = 7.5 Hz, 3H). ^{13}C ($CDCl_3$, 75 MHz): δ 203.7, 198.1, 136.6, 133.3, 128.7, 128.1, 48.0, 37.1, 21.9, 11.5.

NMR data of the compound matches the reported literature.[S3]

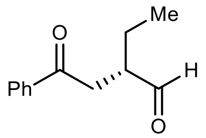

(*S*)-2-Ethyl-4-oxo-4-phenylbutanal (*ent*-(**3**))

Following the general flow procedure described in section 4.4, 181.8 mg (86% yield) of the title compound was obtained as yellow oil.

HRMS (ESI) calculated for $C_{12}H_{15}O_2$ (M+H): 191.1072, found: 191.1075.

The *ee* was determined by HPLC using a Chiralpak® IC column (heptane–*i*PrOH 90/10, 1.5 mL min^{-1}, 210 nm, 25 °C): t_{major}= 10.392 min, t_{minor}= 13.333 min, *ee*= 86%.

$[\alpha]_D^{25}$= –35.2 (c= 1.00, $CHCl_3$)

NMR data are identical to that of compound **3**.

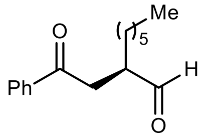

(*R*)-2-(2-Oxo-2-phenylethyl)octanal (**4**)

Following the general flow procedure described in section 4.4, 342.6 mg (84% yield) of the title compound was obtained as yellow oil.

HRMS (ESI) calculated for $C_{16}H_{23}O_2$ (M+H): 247.1698, found: 247.1705.

The *ee* was determined by HPLC using a Chiralpak® IC column (heptane–*i*PrOH 90/10, 1.5 mL min^{-1}, 210 nm, 25 °C): t_{minor}= 8.155 min, t_{major}= 11.029 min, *ee*= 88%.

$[\alpha]_D^{25}$= +46.4 (c= 1.30, $CHCl_3$) {lit.[S4] $[\alpha]_D^{23}$= +66.9 (c= 1.30, $CHCl_3$)}.

^{1}H ($CDCl_3$, 300 MHz): δ 9.85 (s, 1H), 8.04-7.95 (m, 2H), 7.64-7.55 (m, 1H), 7.53-7.44 (m, 2H), 3.49 (dd, *J* = 7.5 Hz, 17.5 Hz, 1H), 3.17-2.99 (m, 2H), 1.89-1.75 (m, 1H), 1.63-1.49 (m, 1H), 1.45-1.25 (m, 8H), 0.90 (t, *J* = 7.2 Hz, 3H). ^{13}C ($CDCl_3$, 75 MHz): δ 203.7, 198.0, 136.6, 133.3, 128.6, 128.1, 46.8, 37.7, 31.6, 29.3, 28.9, 27.1, 22.6, 14.0.

NMR data of the compound matches the reported literature.[S4]

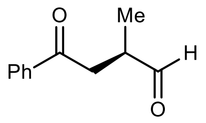

(R)-2-Methyl-4-oxo-4-phenylbutanal (**5**)

Following the general flow procedure described in section 4.4, 312.0 mg (80% yield) of the title compound was obtained as yellow oil.

HRMS (ESI) calculated for $C_{11}H_{13}O_2$ (M+H): 177.0916, found: 177.0915.

The *ee* was determined by HPLC using a Chiralpak® IC column (heptane–*i*PrOH 90/10, 1.5 mL min^{-1}, 210 nm, 25 °C): t_{minor}= 10.086 min, t_{major}= 11.949 min, *ee*= 80%.

$[\alpha]_D^{25}$= +49.9 (c= 1.00, $CHCl_3$)

^{1}H ($CDCl_3$, 300 MHz): δ 9.80 (s, 1H), 8.04-7.95 (m, 2H), 7.60-7.55 (m, 1H), 7.53-7.44 (m, 2H), 3.51 (dd, J = 6.4 Hz, 17.7 Hz, 1H), 3.20-2.98 (m, 2H), 1.25 (d, J = 7.3 Hz, 3H). ^{13}C ($CDCl_3$, 75 MHz): δ 203.5, 197.8, 136.5, 133.4, 128.7, 128.1, 41.6, 39.4, 13.8.

NMR data of the compound matches the reported literature.[S5]

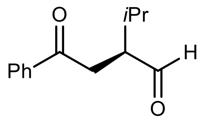

(S)-2-Isopropyl-4-oxo-4-phenylbutanal (**6**)

Following the general flow procedure described in section 4.4, 254.2 mg (75% yield) of the title compound was obtained as yellow oil.

HRMS (ESI) calculated for $C_{13}H_{17}O_2$ (M+H): 205.1229, found: 205.1235.

The *ee* was determined by HPLC using a Chiralpak® IC column (heptane–*i*PrOH 90/10, 1.5 mL min^{-1}, 210 nm, 25 °C): t_{minor}= 8.684 min, t_{major}= 11.743 min, *ee*= 87%.

$[\alpha]_D^{25}$= +87.9 (c= 1.00, $CHCl_3$) {lit.[S6] $[\alpha]_D^{25}$= +6.5 (c= 0.20, CH_2Cl_2)}.

^{1}H ($CDCl_3$, 300 MHz): δ 9.88 (s, 1H), 8.04-7.97 (m, 2H), 7.63-7.54 (m, 1H), 7.53-7.44 (m, 2H), 3.54 (dd, J = 9.1 Hz, 18.0 Hz, 1H), 3.20-3.10 (m, 1H), 2.93 (dd, J = 3.7 Hz, 18.1 Hz, 1H), 2.35-2.21 (m, 1H),), 1.09 (d, J = 7.0 Hz, 3H), 1.02 (d, J = 6.9 Hz, 3H). ^{13}C ($CDCl_3$, 75 MHz): δ 203.8, 198.4, 136.7, 133.2, 128.6, 128.1, 52.6, 34.3, 27.9, 20.5, 19.6.

NMR data of the compound matches the reported literature.[S6]

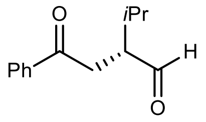

(R)-2-Isopropyl-4-oxo-4-phenylbutanal (*ent*-(**6**))

Following the general flow procedure described in section 4.4, 174.7 mg (77% yield) of the title compound was obtained as yellow oil.

HRMS (ESI) calculated for $C_{13}H_{17}O_2$ (M+H): 205.1229, found: 205.1233.

The *ee* was determined by HPLC using a Chiralpak® IC column (heptane–*i*PrOH 90/10, 1.5 mL min^{-1}, 210 nm, 25 °C): t_{major}= 8.638 min, t_{minor}= 11.855 min, *ee*= 85%.

$[\alpha]_D^{25}$= –66.3 (c= 1.00, $CHCl_3$)

NMR data are identical to that of compound **6**.

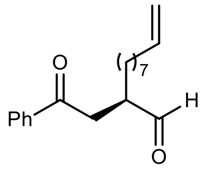

(R)-2-(2-Oxo-2-phenylethyl)undec-10-enal (**7**)

Following the general flow procedure described in section 4.4, 340.3 mg (86% yield) of the title compound was obtained as yellow oil.

HRMS (ESI) calculated for $C_{19}H_{27}O_2$ (M+H): 287.2011, found: 287.2020.

The *ee* was determined by HPLC using a Chiralpak® IC column (heptane–*i*PrOH 90/10, 1.5 mL min^{-1}, 210 nm, 25 °C): t_{minor}= 7.748 min, t_{major}= 10.434 min, *ee*= 87%.

$[\alpha]_D^{25}$= +34.9 (c= 0.66, $CHCl_3$) {lit.[S4] $[\alpha]_D^{23}$= +56.5 (c= 0.66, $CHCl_3$)}.

^{1}H ($CDCl_3$, 300 MHz): δ 9.85 (s, 1H), 8.04-7.95 (m, 2H), 7.65-7.55 (m, 1H), 7.54-7.44 (m, 2H), 5.90-5.75 (m, 1H), 5.06-4.90 (m, 2H), 3.50 (dd, J = 7.4 Hz, 17.2 Hz, 1H), 3.19-3.00 (m, 2H), 2.11-1.99 (m, 2H), 1.90-1.75 (m, 1H), 1.63-1.49 (m, 1H), 1.44-1.26 (m, 11H). ^{13}C ($CDCl_3$, 75 MHz): δ 203.6, 198.0, 139.1, 136.6, 133.3, 128.6, 128.1, 114.2, 46.8, 37.7, 33.8, 29.6, 29.2, 29.0, 28.9, 28.8, 27.1.

NMR data of the compound matches the reported literature.[S4]

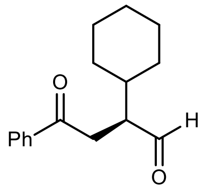

(S)-2-Cyclohexyl-4-oxo-4-phenylbutanal (**8**)

Following the general flow procedure described in section 4.4, 365.9 mg (90% yield) of the title compound was obtained as yellow oil.

HRMS (ESI) calculated for $C_{16}H_{21}O_2$ (M+H): 245.1542, found: 245.1544.

The *ee* was determined by HPLC using a Chiralpak® IC column (heptane–*i*PrOH 90/10, 1.5 mL min^{-1}, 210 nm, 25 °C): t_{minor}= 10.257 min, t_{major}= 15.314 min, *ee*= 87%.

$[\alpha]_D^{25}$= +80.6 (c= 0.46, $CHCl_3$) {lit.[S4] $[\alpha]_D^{23}$= +105.4 (c= 0.46, $CHCl_3$)}.

^{1}H ($CDCl_3$, 300 MHz): δ 9.89 (s, 1H), 8.04-7.96 (m, 2H), 7.64-7.54 (m, 1H), 7.53-7.43 (m, 2H), 3.55 (dd, J = 9.0 Hz, 18.1 Hz, 1H), 3.18-3.09 (m, 1H), 2.96 (dd, J = 3.7 Hz, 17.7 Hz, 1H), 1.94-1.61 (m, 6H), 1.39-1.09 (m, 5H). ^{13}C ($CDCl_3$, 75 MHz): δ 204.0, 198.4, 136.7, 133.2, 128.6, 128.1, 52.2, 38.1, 34.8, 31.1, 30.2, 26.5, 26.2.

NMR data of the compound matches the reported literature.[S4]

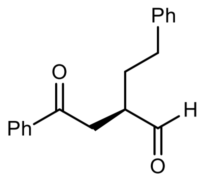

(R)-4-Oxo-2-phenethyl-4-phenylbutanal (**9**)

Following the general flow procedure described in section 4.4, 231.1 mg (78% yield) of the title compound was obtained as amber oil.

HRMS (ESI) calculated for $C_{18}H_{19}O_2$ (M+H): 267.1385, found: 267.1391.

The *ee* was determined by HPLC using a Chiralpak® IC column (heptane–*i*PrOH 90/10, 1.5 mL min^{-1}, 210 nm, 25 °C): t_{minor}= 12.134 min, t_{major}= 15.611 min, *ee*= 82%.

$[\alpha]_D^{25}$= +24.4 (c= 1.00, $CHCl_3$)

^1H ($CDCl_3$, 300 MHz): δ 9.85 (s, 1H), 8.03-7.95 (m, 2H), 7.65-7.57 (m, 1H), 7.55-7.45 (m, 2H), 7.36-7.28 (m, 2H), 7.27-7.18 (m, 3H), 3.59-3.47 (m, 1H), 3.22-3.06 (m, 2H), 2.76 (t, *J* = 7.9 Hz, 2H), 2.26-2.11 (m, 1H), 1.97-1.82 (m, 1H). ^{13}C ($CDCl_3$, 75 MHz): δ 204.0, 198.4, 136.7, 133.2, 128.6, 128.1, 52.2, 38.1, 34.8, 31.1, 30.2, 26.5, 26.2.

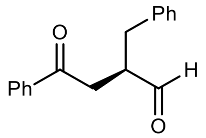

(*R*)-2-Benzyl-4-oxo-4-phenylbutanal (**10**)

Following the general flow procedure described in section 4.4, 241.0 mg (86% yield) of the title compound was obtained as yellow oil.

HRMS (ESI) calculated for $C_{17}H_{17}O_2$ (M+H): 253.1229, found: 253.1233.

The *ee* was determined by HPLC using a Chiralpak® IC column (heptane–*i*PrOH 90/10, 1.5 mL min^{-1}, 210 nm, 25 °C): t_{minor}= 10.553 min, t_{major}= 12.881 min, *ee*= 81%.

$[\alpha]_D^{25}$= +14.5 (c= 1.00, $CHCl_3$) {lit.[S4] $[\alpha]_D^{25}$= +25.4 (c= 1.00, $CHCl_3$)}.

^1H ($CDCl_3$, 300 MHz): δ 9.92 (s, 1H), 7.99-7.89 (m, 2H), 7.62-7.55 (m, 1H), 7.51-7.42 (m, 2H), 7.37-7.20 (m, 5H), 3.52-3.36 (m, 2H), 3.26-3.14 (m, 1H), 3.11-2.99 (m, 1H), 2.90-2.79 (m, 1H). ^{13}C ($CDCl_3$, 75 MHz): δ 203.0, 197.9, 138.2, 136.5, 133.4, 129.1, 128.8, 128.6, 128.1, 126.8, 48.4, 37.3, 34.8.

NMR data of the compound matches the reported literature.[S4]

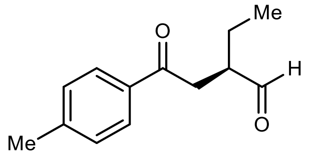

(*R*)-2-Ethyl-4-oxo-4-(*p*-tolyl)butanal (**13**)

Following the general flow procedure described in section 4.4, 253.7 mg (90% yield) of the title compound was obtained as pale-yellow oil.

HRMS (ESI) calculated for $C_{13}H_{17}O_2$ (M+H): 205.1229, found: 205.1230.

The *ee* was determined by HPLC using a Chiralpak® IC column (heptane–*i*PrOH 90/10, 1.5 mL min^{-1}, 210 nm, 25 °C): t_{minor}= 13.401 min, t_{major}= 15.836 min, *ee*= 82%.

$[\alpha]_D^{25}$= +54.0 (c= 1.00, $CHCl_3$)

^1H ($CDCl_3$, 300 MHz): δ 9.84 (s, 1H), 7.89 (d, *J* = 8.0 Hz, 2H), 7.27 (d, *J* = 7.8 Hz, 2H), 3.53-3.39 (m, 1H), 3.13-2.95 (m, 1H), 2.42 (s, 3H), 1.93-1.78 (m, 1H), 1.72-1.58 (m, 1H), 1.02 (t, *J* = 7.5 Hz, 3H). ^{13}C ($CDCl_3$, 75 MHz): δ 203.7, 197.6, 144.1, 134.1, 129.3, 128.2, 48.1, 37.0, 21.9, 21.7, 11.5.

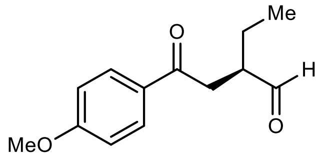

(*R*)-2-Ethyl-4-(4-methoxyphenyl)-4-oxobutanal (**14**)

Following the general flow procedure described in section 4.4, 268.8 mg (88% yield) of the title compound was obtained as colorless oil.

HRMS (ESI) calculated for $C_{13}H_{17}O_3$ (M+H): 221.1178, found: 221.1185.

The *ee* was determined by HPLC using a Chiralpak® IC column (heptane–*i*PrOH 90/10, 1.5 mL min^{-1}, 210 nm, 25 °C): t_{minor}= 26.726 min, t_{major}= 28.117 min, *ee*= 82%.

$[\alpha]_D^{25}$= +50.4 (c= 1.00, $CHCl_3$)

^1H ($CDCl_3$, 300 MHz): δ 9.84 (s, 1H), 7.97 (d, *J* = 9.1 Hz, 2H), 6.95 (d, *J* = 9.1 Hz, 2H), 3.89 (s, 3H), 3.49-3.37 (m, 1H), 3.10-2.95 (m, 1H), 1.94-1.77 (m, 1H), 1.71-1.55 (m, 1H), 1.02 (t, *J* = 7.5 Hz, 3H). ^{13}C ($CDCl_3$, 75 MHz): δ 203.8, 196.5, 163.6, 130.4, 129.7, 113.8, 55.5, 48.1, 36.8, 21.9, 11.5.

NMR data of the compound matches the reported literature.[S7]

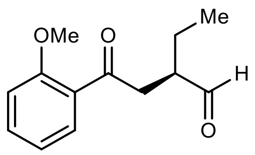

(*R*)-2-Ethyl-4-(2-methoxyphenyl)-4-oxobutanal (**15**)

Following the general flow procedure described in section 4.4, 250.9 mg (82% yield) of the title compound was obtained as yellow oil.

HRMS (ESI) calculated for $C_{13}H_{17}O_3$ (M+H): 221.1178, found: 221.1187.

The *ee* was determined by HPLC using a Chiralpak® IC column (heptane–*i*PrOH 90/10, 1.5 mL min^{-1}, 210 nm, 25 °C): t_{minor}= 16.963 min, t_{major}= 20.778 min, *ee*= 82%.

$[\alpha]_D^{25}$= +28.4 (c= 1.00, $CHCl_3$)

^1H ($CDCl_3$, 300 MHz): δ 9.81 (s, 1H), 7.73 (dd, *J* = 1.8 Hz, 7.6 Hz, 1H), 7.54-7.43 (m, 1H), 7.07-6.93 (m, 2H), 3.93 (s, 3H), 3.44 (dd, *J* = 8.1 Hz, 18.3 Hz, 1H), 3.13 (dd, *J* = 5.2 Hz, 18.3 Hz, 1H), 3.03-2.92 (m, 1H), 1.91-1.74 (m, 1H), 1.68-1.53 (m, 1H), 1.00 (t, *J* = 7.5 Hz, 3H). ^{13}C ($CDCl_3$, 75 MHz): δ 204.1, 200.1, 158.7, 133.8, 130.5, 127.6, 120.7, 111.6, 55.5, 48.6, 42.8, 21.9, 11.6.

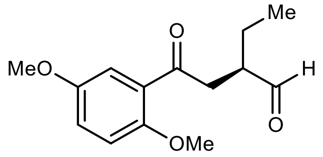

(*R*)-4-(2,5-Dimethoxyphenyl)-2-ethyl-4-oxobutanal (**16**)

Following the general flow procedure described in section 4.4, 327.8 mg (79% yield) of the title compound was obtained as yellow oil.

HRMS (ESI) calculated for $C_{14}H_{19}O_4$ (M+H): 251.1283, found: 251.1294.

The *ee* was determined by HPLC using a Chiralpak® IA column (heptane–*i*PrOH 90/10, 1.0 mL min^{-1}, 254 nm, 25 °C): t_{major}= 8.927 min, t_{minor}= 9.901 min, *ee*= 81%.

$[\alpha]_D^{25}$= +25.4 (c= 1.00, $CHCl_3$)

^1H ($CDCl_3$, 300 MHz): δ 9.81 (s, 1H), 7.28 (d, *J* = 3.1 Hz, 1H), 7.06 (dd, *J* = 3.2 Hz, 9.0 Hz, 1H), 6.93 (d, *J* = 8.9 Hz, 1H), 3.89 (s, 3H), 3.80 (s, 3H), 3.44 (dd, *J* = 7.9 Hz, 18.3 Hz, 1H), 3.14 (dd, *J* = 5.0 Hz, 18.4 Hz, 1H), 3.02-2.90 (m, 1H), 1.91-1.74 (m, 1H), 1.67-1.51 (m, 1H), 1.00 (t, *J* = 7.5 Hz, 3H). ^{13}C ($CDCl_3$, 75 MHz): δ 204.1, 199.6, 153.4, 153.3, 127.6, 120.6, 113.8, 113.1, 56.0, 55.8, 48.6, 42.8, 21.9, 11.6.

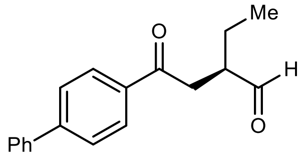

(*R*)-4-([1,1'-Biphenyl]-4-yl)-2-ethyl-4-oxobutanal (**17**)

Following the general flow procedure described in section 4.4, 294.3 mg (80% yield) of the title compound was obtained as yellowish solid.

HRMS (ESI) calculated for $C_{18}H_{19}O_2$ (M+H): 267.1385, found: 267.1395.

The *ee* was determined by HPLC using a Chiralpak® IC column (heptane–*i*PrOH 90/10, 1.0 mL min^{-1}, 254 nm, 25 °C): t_{minor}= 12.105 min, t_{major}= 19.346 min, *ee*= 84%.

$[\alpha]_D^{25}$= +42.1 (c= 1.00, $CHCl_3$) {lit.[S3] $[\alpha]_D^{26}$= +27.0 (c= 1.00, $CHCl_3$)}.

^{1}H ($CDCl_3$, 300 MHz): δ 9.87 (s, 1H), 8.08 (d, *J* = 9.1 Hz, 2H), 7.75-7.62 (m, 4H), 7.55-7.40 (m, 3H), 3.59-3.46 (m, 1H), 3.18-3.01 (m, 2H), 1.98-1.80 (m, 1H), 1.76-1.62 (m, 1H), 1.06 (t, *J* = 7.5 Hz, 3H). ^{13}C ($CDCl_3$, 75 MHz): δ 203.7, 197.7, 146.0, 139.8, 135.3, 129.0, 128.7, 128.3, 127.3, 48.1, 37.1, 21.9, 11.5.

NMR data of the compound matches the reported literature.[S3]

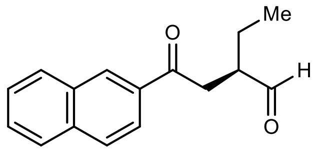

(*R*)-2-Ethyl-4-(naphthalen-2-yl)-4-oxobutanal (**18**)

Following the general flow procedure described in section 4.4, 283.6 mg (85% yield) of the title compound was obtained as yellow oil.

HRMS (ESI) calculated for $C_{16}H_{17}O_2$ (M+H): 241.1229, found: 241.1236.

The *ee* was determined by HPLC using a Chiralcel® OD-H column (heptane–*i*PrOH 90/10, 1.0 mL min^{-1}, 254 nm, 25 °C): t_{major}= 9.900 min, t_{minor}= 11.268 min, *ee*= 76%.

$[\alpha]_D^{25}$= +49.1 (c= 1.00, $CHCl_3$) {lit.[S3] $[\alpha]_D^{27}$= +23.9 (c= 1.00, $CHCl_3$)}.

^{1}H ($CDCl_3$, 300 MHz): δ 9.89 (s, 1H), 8.53 (s, 1H), 8.06 (dd, *J* = 1.8 Hz, 8.6 Hz, 1H), 8.00 (d, *J* = 8.1 Hz, 1H), 7.95-7.88 (m, 2H), 7.67-7.55 (m, 2H), 3.71-3.58 (m, 1H), 3.25-3.10 (m, 2H), 2.00-1.83 (m, 1H), 1.78-1.60 (m, 1H), 1.07 (t, *J* = 7.4 Hz, 3H). ^{13}C ($CDCl_3$, 75 MHz): δ 203.7, 198.0, 135.7, 133.9, 132.5, 129.9, 129.6, 128.6, 128.5, 127.8, 126.9, 123.8, 48.2, 37.1, 21.9, 11.5.

NMR data of the compound matches the reported literature.[S3]

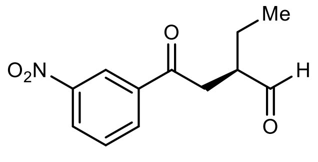

(*R*)-2-Ethyl-4-(3-nitrophenyl)-4-oxobutanal (**19**)

Following the general flow procedure described in section 4.4, 182.0 mg (70% yield) of the title compound was obtained as amber oil.

HRMS (ESI) calculated for $C_{12}H_{14}NO_4$ (M+H): 236.0923, found: 236.0919.

The *ee* was determined by HPLC using a Chiralpak® IC column (heptane–*i*PrOH 90/10, 1.0 mL min^{-1}, 254 nm, 25 °C): t_{minor}= 16.149 min, t_{major}= 18.503 min, *ee*= 80%.

$[\alpha]_D^{25}$= +65.1 (c= 1.00, $CHCl_3$) {lit.[S3] $[\alpha]_D^{25}$= +56.4 (c= 1.00, $CHCl_3$)}.

^{1}H ($CDCl_3$, 300 MHz): δ 9.83 (s, 1H), 8.81 (t, *J* = 1.8 Hz, 1H), 8.48-8.42 (m, 1H), 8.35-8.30 (m, 1H), 7.71 (t, *J* = 8.0 Hz, 1H), 3.55 (dd, *J* = 8.3 Hz, 18.0 Hz, 1H), 3.22-3.11 (m, 1H), 3.00 (dd, *J* = 5.0 Hz, 18.2 Hz, 1H), 1.99-1.83 (m, 1H), 1.77-1.61 (m, 1H), 1.07 (t, *J* = 7.5 Hz, 1H). ^{13}C ($CDCl_3$, 75 MHz): δ 203.1, 196.0, 148.4, 137.8, 133.7, 130.0, 127.6, 123.0, 48.1, 37.0, 21.7, 11.5.

NMR data of the compound matches the reported literature.[S3]

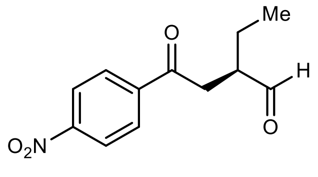

(*R*)-2-Ethyl-4-(4-nitrophenyl)-4-oxobutanal (**20**)

Following the general flow procedure described in section 4.4, 193.2 mg (74% yield) of the title compound was obtained as off-white solid.

HRMS (ESI) calculated for $C_{12}H_{14}NO_4$ (M+H): 236.0923, found: 236.0913.

The *ee* was determined by HPLC using a Chiralpak® IC column (heptane–*i*PrOH 80/20, 1.0 mL min^{-1}, 254 nm, 25 °C): t_{minor}= 22.386 min, t_{major}= 24.613 min, *ee*= 91%.

$[\alpha]_D^{25}$= +95.0 (c= 1.00, $CHCl_3$) {lit.[S3] $[\alpha]_D^{27}$= +51.8 (c= 1.00, $CHCl_3$)}.

^{1}H ($CDCl_3$, 300 MHz): δ 9.83 (s, 1H), 8.35 (d, *J* = 9.0 Hz, 2H), 8.15 (d, *J* = 8.9 Hz, 2H), 3.54 (dd, *J* = 8.2 Hz, 17.6 Hz, 1H), 3.21-3.10 (m, 1H), 2.98 (dd, *J* = 4.2 Hz, 18.0 Hz, 1H), 1.99-1.83 (m, 1H), 1.78-1.63 (m, 1H), 1.06 (t, *J* = 7.4 Hz, 1H). ^{13}C ($CDCl_3$, 75 MHz): δ 203.0, 196.7, 150.4, 141.1, 129,1, 123.9, 48.2, 37.2, 21.8, 11.5.

NMR data of the compound matches the reported literature.[S3]

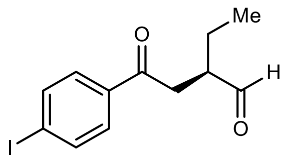

(*R*)-2-Ethyl-4-(4-iodophenyl)-4-oxobutanal (**21**)

Following the general flow procedure described in section 4.4, 311.4 mg (89% yield) of the title compound was obtained as dark-yellow oil.

HRMS (ESI) calculated for $C_{12}H_{14}IO_2$ (M+H): 317.0038, found: 317.0042.

The *ee* was determined by HPLC using a Chiralpak® IC column (heptane–*i*PrOH 90/10, 1.5 mL min^{-1}, 210 nm, 25 °C): t_{minor}= 9.443 min, t_{major}= 10.426 min, *ee*= 80%.

$[\alpha]_D^{25}$= +39.8 (c= 1.00, $CHCl_3$).

^{1}H ($CDCl_3$, 300 MHz): δ 9.83 (s, 1H), 7.85 (d, *J* = 7.8 Hz, 2H), 7.7 (d, *J* = 7.7 Hz, 2H), 3.44 (dd, *J* = 7.9 Hz, 17.7 Hz, 1H), 3.14-3.03 (m, 1H), 2.92 (dd, *J* = 4.7 Hz, 17.8 Hz, 1H), 1.94-1.78 (m, 1H), 1.73-1.57 (m, 1H), 1.03 (t, *J* = 7.4 Hz, 1H). ^{13}C ($CDCl_3$, 75 MHz): δ 203.4, 197.4, 138.0, 135.8, 129.5, 101.3, 48.0, 36.9, 21.8, 11.5.

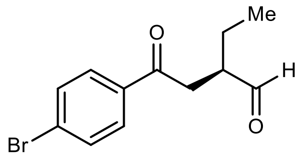

(*R*)-4-(4-Bromophenyl)-2-ethyl-4-oxobutanal (**22**)

Following the general flow procedure described in section 4.4, 335.9 mg (90% yield) of the title compound was obtained as amber oil.

HRMS (ESI) calculated for $C_{12}H_{14}BrO_2$ (M+H): 269.0177, found: 269.0172.

The *ee* was determined by HPLC using a Chiralpak® IC column (heptane–*i*PrOH 90/10, 1.5 mL min^{-1}, 210 nm, 25 °C): t_{minor}= 8.609 min, t_{major}= 9.783 min, *ee*= 72%.

$[\alpha]_D^{25}$= +38.2 (c= 1.00, $CHCl_3$)

^{1}H ($CDCl_3$, 300 MHz): δ 9.82 (s, 1H), 7.85 (d, *J* = 8.7 Hz, 2H), 7.6 (d, *J* = 8.8 Hz, 2H), 3.45 (dd, *J* = 7.8 Hz, 17.8 Hz, 1H), 3.14-3.03 (m, 1H), 2.95 (dd, *J* = 4.8 Hz, 17.8 Hz, 1H), 1.94-1.78 (m, 1H), 1.74-1.56 (m, 1H), 1.03 (t, *J* = 7.6 Hz, 1H). ^{13}C ($CDCl_3$, 75 MHz): δ 203.4, 197.1, 135.3, 132.0, 129.6, 128.5, 48.0, 36.9, 21.8, 11.5.

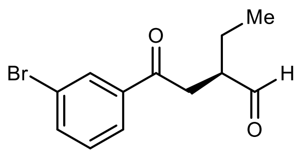

(*R*)-4-(3-Bromophenyl)-2-ethyl-4-oxobutanal (**23**)

Following the general flow procedure described in section 4.4, 300.3 mg (81% yield) of the title compound was obtained as yellow oil.

HRMS (ESI) calculated for $C_{12}H_{14}BrO_2$ (M+H): 269.0177, found: 269.0169.

The *ee* was determined by HPLC using a Chiralpak® IC column (heptane–*i*PrOH 90/10, 1.5 mL min^{-1}, 210 nm, 25 °C): t_{minor}= 7.797 min, t_{major}= 9.660 min, *ee*= 78%.

$[\alpha]_D^{25}$= +46.4 (c= 1.00, $CHCl_3$) {lit.[S3] $[\alpha]_D^{27}$= +46.1 (c= 1.00, $CHCl_3$)}.

^{1}H ($CDCl_3$, 300 MHz): δ 9.82 (s, 1H), 8.11 (t, *J* = 1.8 Hz, 1H), 7.93-7.89 (m, 1H), 7.73-7.67 (m, 1H), 7.36 (t, *J* = 8.0 Hz, 1H), 3.45 (dd, *J* = 7.9 Hz, 17.9 Hz, 1H), 3.14-3.04 (m, 1H), 2.96 (dd, *J* = 5.1 Hz, 18.0 Hz, 1H), 1.95-1.78 (m, 1H), 1.74-1.56 (m, 1H), 1.03 (t, *J* = 7.5 Hz, 1H). ^{13}C ($CDCl_3$, 75 MHz): δ 203.3, 196.7, 138.3, 136.1, 131.2, 130.3, 126.6, 123.0, 48.0, 37.0, 21.8, 11.5.

NMR data of the compound matches the reported literature.[S3]

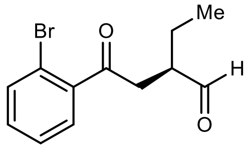

(*R*)-4-(2-Bromophenyl)-2-ethyl-4-oxobutanal (**24**)

Following the general flow procedure described in section 4.4, 309.4 mg (83% yield) of the title compound was obtained as amber oil.

HRMS (ESI) calculated for $C_{12}H_{14}BrO_2$ (M+H): 269.0177, found: 269.0175.

The *ee* was determined by HPLC using a Chiralpak® IC column (heptane–*i*PrOH 90/10, 1.0 mL min^{-1}, 210 nm, 25 °C): t_{minor}= 13.571 min, t_{major}= 14.859 min, *ee*= 71%.

$[\alpha]_D^{25}$= +20.7 (c= 1.00, $CHCl_3$)

^{1}H ($CDCl_3$, 300 MHz): δ 9.80 (s, 1H), 7.62 (dd, *J* = 1.2 Hz, 7.9 Hz, 1H), 7.51 (dd, *J* = 1.8 Hz, 7.5 Hz, 1H), 7.43-7.37 (m, 1H), 7.35-7.28 (m, 1H), 3.36 (dd, *J* = 8.0 Hz, 17.6 Hz, 1H), 3.14-2.95 (m, 2H), 1.95-1.79 (m, 1H), 1.74-1.59 (m, 1H), 1.03 (t, *J* = 7.5 Hz, 1H). ^{13}C ($CDCl_3$, 75 MHz): δ 203.2, 202.2, 141.3, 133.7, 131.7, 128.7, 127.5, 118.5, 48.5, 41.0, 21.6, 11.4.

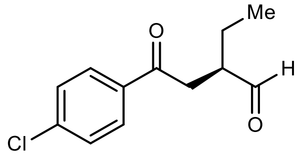

(*R*)-4-(4-Chlorophenyl)-2-ethyl-4-oxobutanal (**25**)

Following the general flow procedure described in section 4.4, 327.3 mg (88% yield) of the title compound was obtained as amber oil.

HRMS (ESI) calculated for $C_{12}H_{14}ClO_2$ (M+H): 225.0682, found: 225.0684.

The *ee* was determined by HPLC using a Chiralpak® IC column (heptane–*i*PrOH 90/10, 1.5 mL min^{-1}, 210 nm, 25 °C): t_{minor}= 8.126 min, t_{major}= 9.506 min, *ee*= 72%.

$[\alpha]_D^{25}$= +53.4 (c= 1.00, $CHCl_3$).

^{1}H ($CDCl_3$, 300 MHz): δ 9.82 (s, 1H), 7.93 (d, *J* = 8.7 Hz, 2H), 7.45 (d, *J* = 8.7 Hz, 2H), 3.45 (dd, *J* = 7.8 Hz, 17.6 Hz, 1H), 3.14-2.90 (m, 2H), 1.94-1.78 (m, 1H), 1.73-1.57 (m, 1H), 1.02 (t, *J* = 7.4 Hz, 1H). ^{13}C ($CDCl_3$, 75 MHz): δ 203.4, 196.9, 139.7, 134.9, 129,5, 128.9, 48.0, 37.0, 21.8, 11.4.

NMR data of the compound matches the reported literature.[S7]

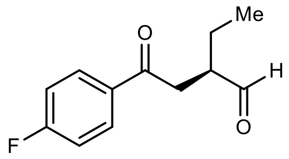

(*R*)-2-Ethyl-4-(4-fluorophenyl)-4-oxobutanal (**26**)

Following the general flow procedure described in section 4.4, 287.9 mg (83% yield) of the title compound was obtained as greenish oil.

HRMS (ESI) calculated for $C_{12}H_{14}FO_2$ (M+H): 209.0978, found: 209.0978.

The *ee* was determined by HPLC using a Chiralpak® IC column (heptane–*i*PrOH 90/10, 1.5 mL min^{-1}, 210 nm, 25 °C): t_{minor}= 8.092 min, t_{major}= 10.519 min, *ee*= 74%.

$[\alpha]_D^{25}$= +51.1 (c= 1.00, $CHCl_3$).

^{1}H ($CDCl_3$, 300 MHz): δ 9.82 (s, 1H), 8.00 (dd, *J* = 5.3 Hz, 8.8 Hz, 2H), 7.14 (t, *J* = 8.6 Hz, 2H), 3.45 (dd, *J* = 7.7 Hz, 17.5 Hz, 1H), 3.12-2.92 (m, 2H), 1.94-1.77 (m, 1H), 1.72-1.56 (m, 1H), 1.02 (t, *J* = 7.4 Hz, 1H). ^{13}C ($CDCl_3$, 75 MHz): δ 203.5, 196.4, 167.5, 164.1, 133.1, 133.0, 130.8, 130.7, 115.9, 115.6, 48.1, 36.9, 21.8, 11.4. (Signal multiplication due to carbon–fluorine coupling.)

NMR data of the compound matches the reported literature.[S7]

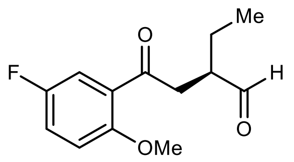

(*R*)-2-Ethyl-4-(5-fluoro-2-methoxyphenyl)-4-oxobutanal (**27**)

Following the general flow procedure described in section 4.4, 254.8 mg (77% yield) of the title compound was obtained as yellow oil.

HRMS (ESI) calculated for $C_{13}H_{16}FO_3$ (M+H): 239.1083, found: 239.1091.

The *ee* was determined by HPLC using a Chiralpak® IC column (heptane–*i*PrOH 90/10, 1.5 mL min^{-1}, 210 nm, 25 °C): t_{minor}= 12.302 min, t_{major}= 14.296 min, *ee*= 75%.

$[\alpha]_D^{25}$= +25.8 (c= 1.00, $CHCl_3$)

^{1}H ($CDCl_3$, 300 MHz): δ 9.79 (s, 1H), 7.44 (dd, *J* = 3.2 Hz, 8.9 Hz, 1H), 7.21-7.13 (m, 1H), 6.93 (dd, *J* = 4.2 Hz, 9.1 Hz, 1H), 3.91 (s, 3H), 3.42 (dd, *J* = 8.1 Hz, 18.6 Hz, 1H), 3.09 (dd, *J* = 4.9 Hz, 18.5 Hz, 1H), 3.03-2.91 (m, 1H), 1.90-1.74 (m, 1H), 1.67-1.50 (m, 1H), 0.99 (t, *J* = 7.4 Hz, 1H). ^{13}C ($CDCl_3$, 75 MHz): δ 203.9, 198.7, 198.7, 158.3, 155.1, 155.0, 155.0, 128.3, 128.3, 120.3, 120.0, 116.8, 116.5, 113.0 112.9, 56.01, 48.5, 42.6, 21.8, 11.5. (Signal multiplication due to carbon–fluorine coupling.)

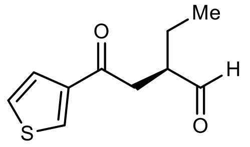

(*R*)-2-Ethyl-4-oxo-4-(thiophen-3-yl)butanal (**28**)

Following the general flow procedure described in section 4.4, 373.2 mg (86% yield) of the title compound was obtained as pale-yellow oil.

HRMS (ESI) calculated for $C_{10}H_{13}O_3S$ (M+H): 197.0636, found: 197.0631.

The *ee* was determined by HPLC using a Chiralpak® IC column (heptane–*i*PrOH 90/10, 1.5 mL min^{-1}, 210 nm, 25 °C): t_{minor}= 13.081 min, t_{major}= 16.688 min, *ee*= 86%.

$[\alpha]_D^{25}$= +59.5 (c= 1.00, $CHCl_3$)

^{1}H ($CDCl_3$, 300 MHz): δ 9.82 (s, 1H), 8.10 (dd, *J* = 1.3 Hz, 3.0 Hz, 1H), 7.56 (dd, *J* = 1.3 Hz, 5.2 Hz, 1H), 7.34 (dd, *J* = 2.8 Hz, 5.0 Hz, 1H), 3.39 (dd, *J* = 7.3 Hz, 17.5 Hz, 1H), 3.10-2.90 (m, 2H), 1.93-1.77 (m, 1H), 1.72-1.56 (m, 1H), 1.01 (t, *J* = 7.4 Hz, 1H). ^{13}C ($CDCl_3$, 75 MHz): δ 203.5, 192.3, 141.8, 132.2, 126.9, 126.5, 48.0, 38.1, 21.8, 11.4.

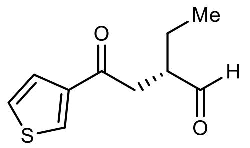

(S)-2-Ethyl-4-oxo-4-(thiophen-3-yl)butanal (*ent*-(**28**))

Following the general flow procedure described in section 4.4, 238.7 mg (88% yield) of the title compound was obtained as yellow oil.

HRMS (ESI) calculated for $C_{10}H_{13}O_3S$ (M+H): 197.0636, found: 197.0629.

The *ee* was determined by HPLC using a Chiralpak® IC column (heptane–*i*PrOH 90/10, 1.5 mL min^{-1}, 210 nm, 25 °C): t_{major}= 13.124 min, t_{minor}= 16.855 min, *ee*= 85%.

$[\alpha]_D^{25}$= –65.0 (c= 1.00, $CHCl_3$)

NMR data are identical to that of compound **28**.

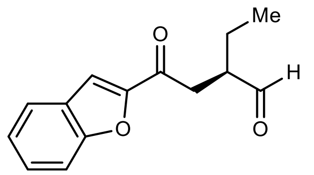

((R)-4-(Benzofuran-2-yl)-2-ethyl-4-oxobutanal (**29**)

Following the general flow procedure described in section 4.4, 246.2 mg (77% yield) of the title compound was obtained as amber oil.

HRMS (ESI) calculated for $C_{14}H_{15}O_3$ (M+H): 231.1021, found: 231.1017.

The *ee* was determined by HPLC using a Chiralpak® IC column (heptane–*i*PrOH 90/10, 1.5 mL min^{-1}, 210 nm, 25 °C): t_{minor}= 17.895 min, t_{major}= 20.638 min, *ee*= 81%.

$[\alpha]_D^{25}$= +57.0 (c= 1.00, $CHCl_3$)

^{1}H ($CDCl_3$, 300 MHz): δ 9.82 (s, 1H), 7.73 (d, J = 8.0 Hz, 1H), 7.62-7.55 (m, 2H), 7.53-7.45 (m, 1H), 7.36-7.28 (m, 1H), 3.48 (dd, J = 7.4 Hz, 17.0 Hz, 1H), 3.18-2.96 (m, 2H), 1.97-1.81 (m, 1H), 1.77-1.61 (m, 1H), 1.04 (t, J = 7.4 Hz, 1H). ^{13}C ($CDCl_3$, 75 MHz): δ 203.1, 189.2, 155.6, 152.3, 128.4, 127.0, 124.0, 123.4, 112.9, 112.5, 47.9, 37.0, 21.8, 11.4.

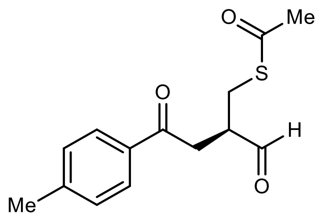

(R)-S-(2-Formyl-4-oxo-4-(*p*-tolyl)butyl) ethanethioate (**31**)

Following the flow procedure described in section 4.7, 573.6 mg (78% yield) of the title compound was obtained as yellow oil.

HRMS (ESI) calculated for $C_{14}H_{17}O_3S$ (M+H): 265.0898, found: 265.0908.

The *ee* was determined by HPLC using a Chiralcel® OD-H column (heptane–*i*PrOH 90/10, 1.0 mL min^{-1}, 254 nm, 25 °C): t_{minor}= 14.900 min, t_{major}= 16.446 min, *ee*= 77%.

$[\alpha]_D^{25}$= +13.1 (c= 1.00, $CHCl_3$)

^{1}H ($CDCl_3$, 300 MHz): δ 9.81 (s, 1H), 7.87 (d, J = 8.2 Hz, 2H), 7.28 (d, J = 8.2 Hz, 2H), 3.55-3.45 (m, 1H), 3.38-3.09 (m, 4H), 2.43 (s, 3H), 2.35 (s, 3H). ^{13}C ($CDCl_3$, 75 MHz): δ 201.4, 196.7, 195.1, 144.5, 133.7, 129.4, 128.3, 46.9, 37.1, 30.6, 27.6, 21.7.

7. Collection of NMR spectra and HPLC chromatograms

300 MHz, $CDCl_3$

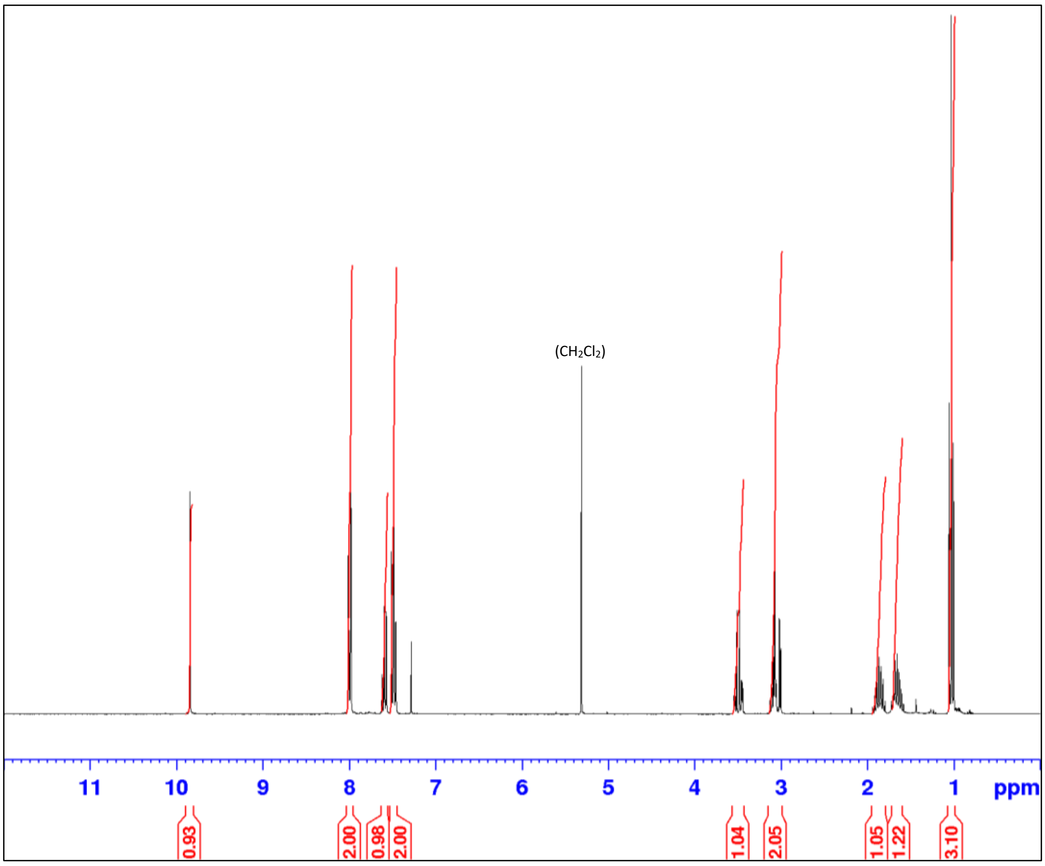

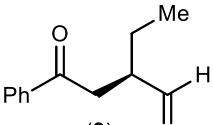

(3)

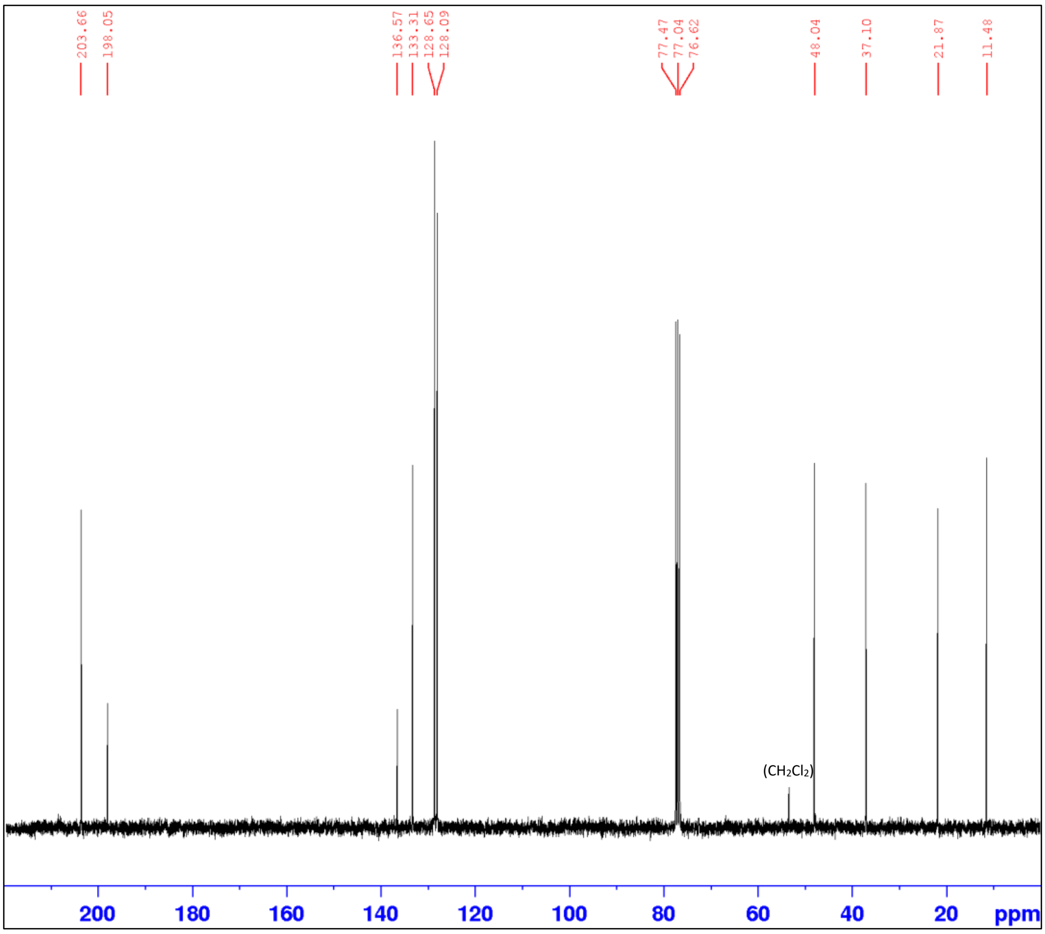

75 MHz, $CDCl_3$

HPLC chromatograms

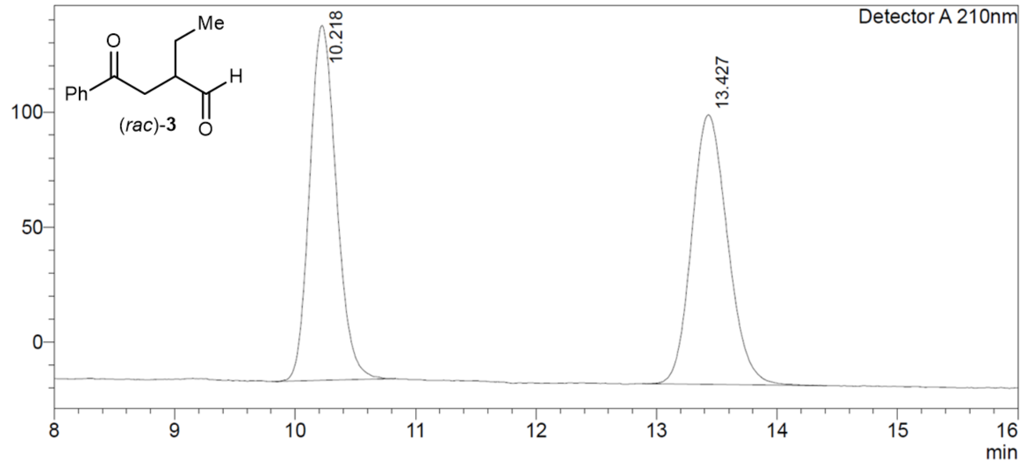

Peak#	Ret. Time	Area	Height	Area%
1	10.218	2386959	154300	49.542
2	13.427	2431133	117260	50.458
Total		4818092	271560	100.000

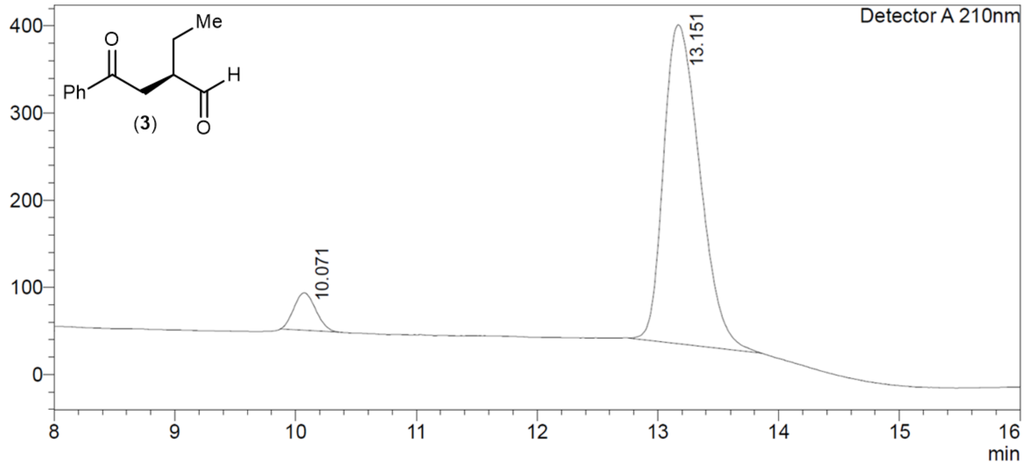

Peak#	Ret. Time	Area	Height	Area%
1	10.071	417733	37822	5.185
2	13.151	7638612	367094	94.815
Total		8056345	404916	100.000

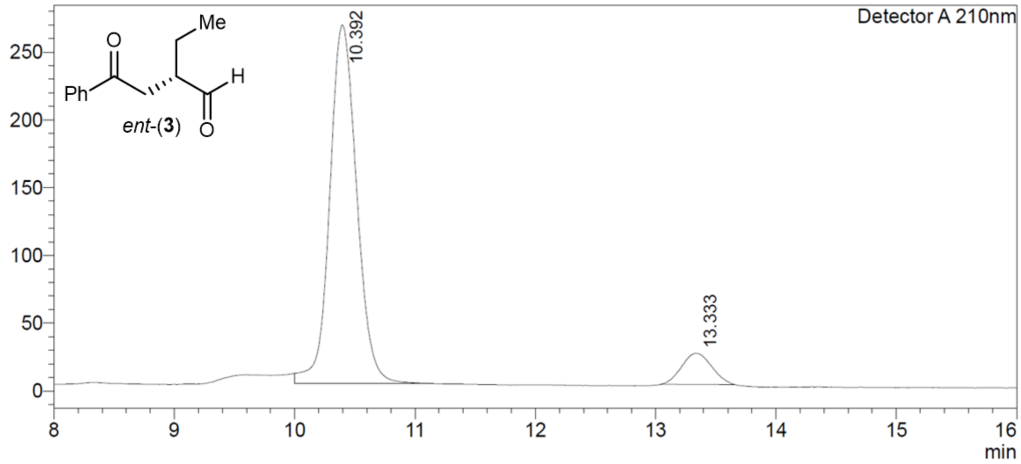

Peak#	Ret. Time	Area	Height	Area%
1	10.392	4135750	264377	92.833
2	13.333	319288	20468	7.167
Total		4455038	284846	100.000

300 MHz, CDCl3

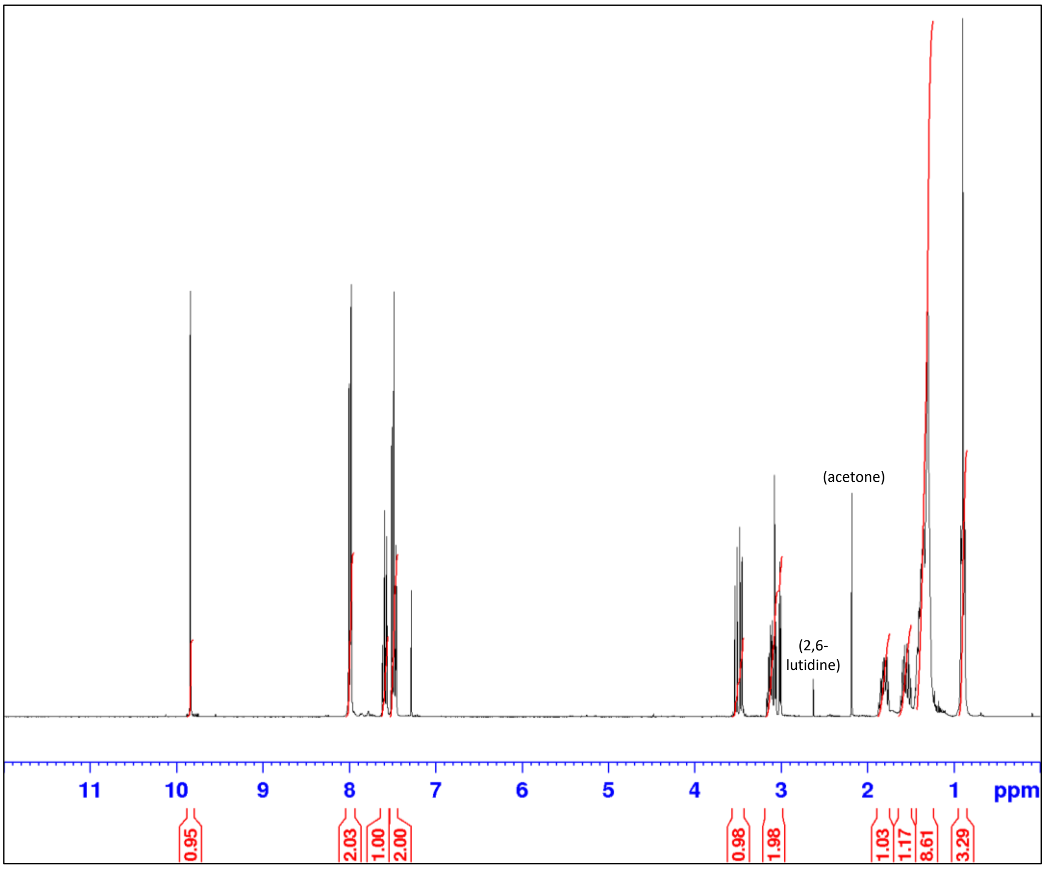

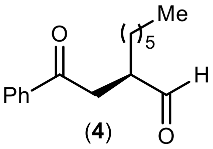

(**4**)

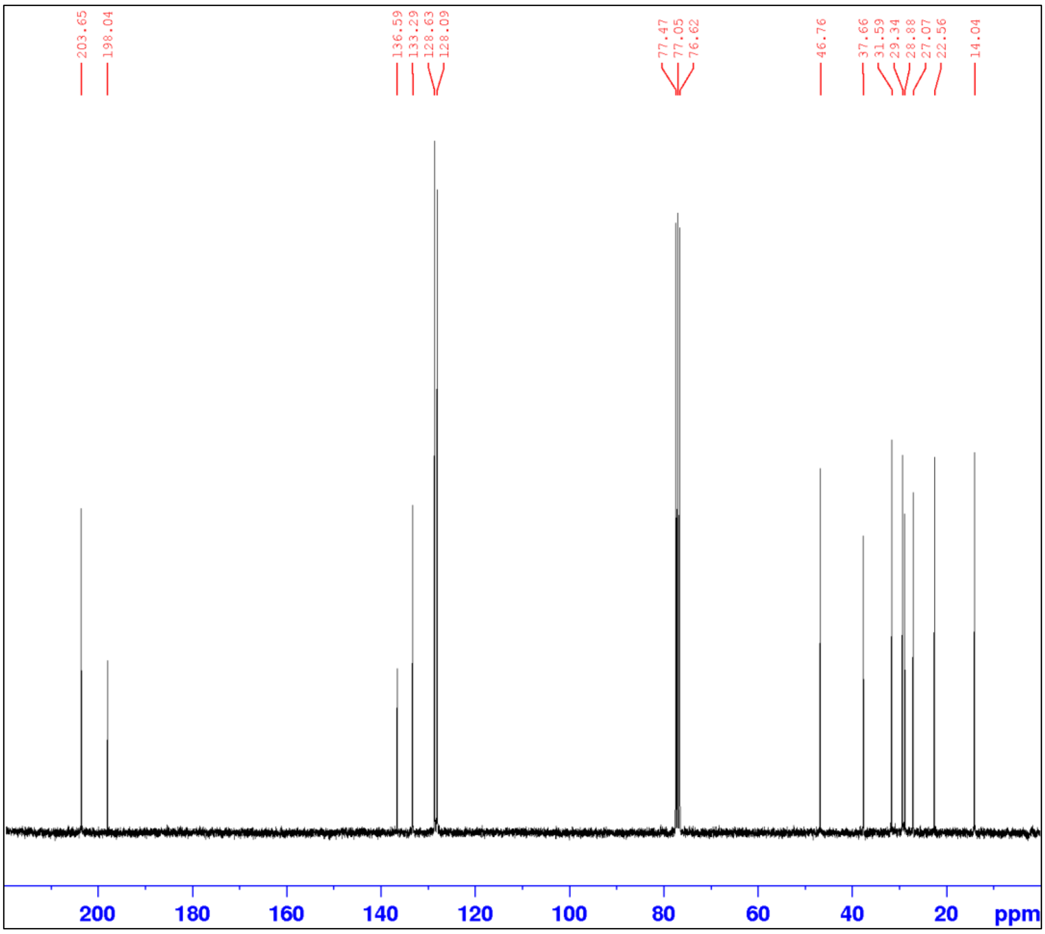

75 MHz, CDCl3

HPLC chromatograms

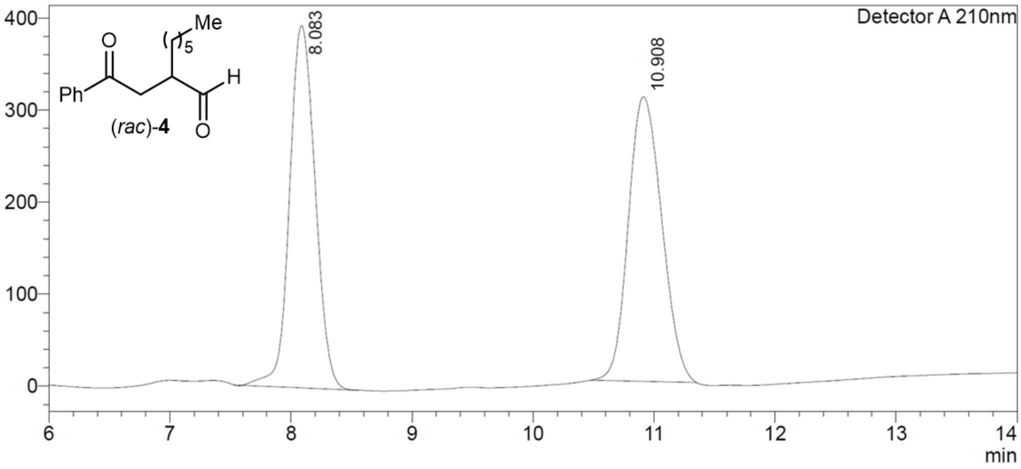

Peak#	Ret. Time	Area	Height	Area%
1	8.083	5807518	393766	49.371
2	10.908	5955508	309355	50.629
Total		11763026	703121	100.000

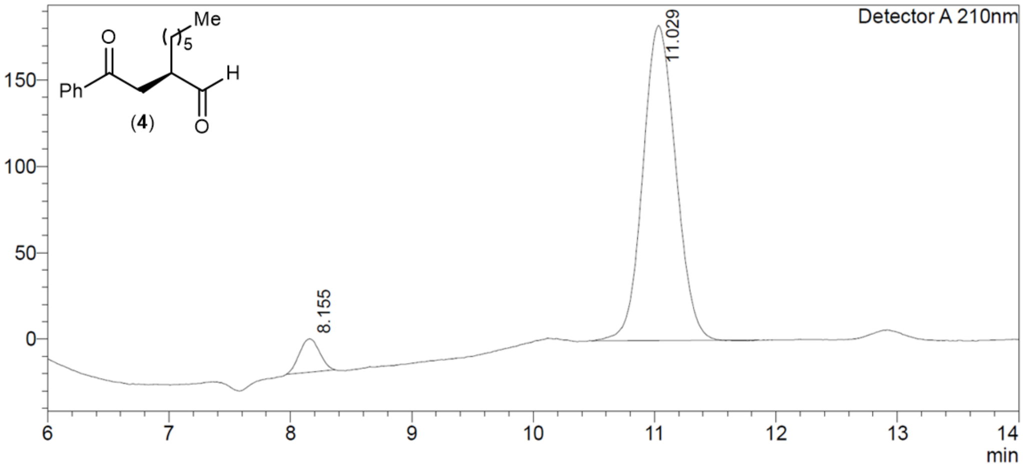

Peak#	Ret. Time	Area	Height	Area%
1	8.155	223209	19328	6.090
2	11.029	3442064	182496	93.910
Total		3665273	201825	100.000

300 MHz, $CDCl_3$

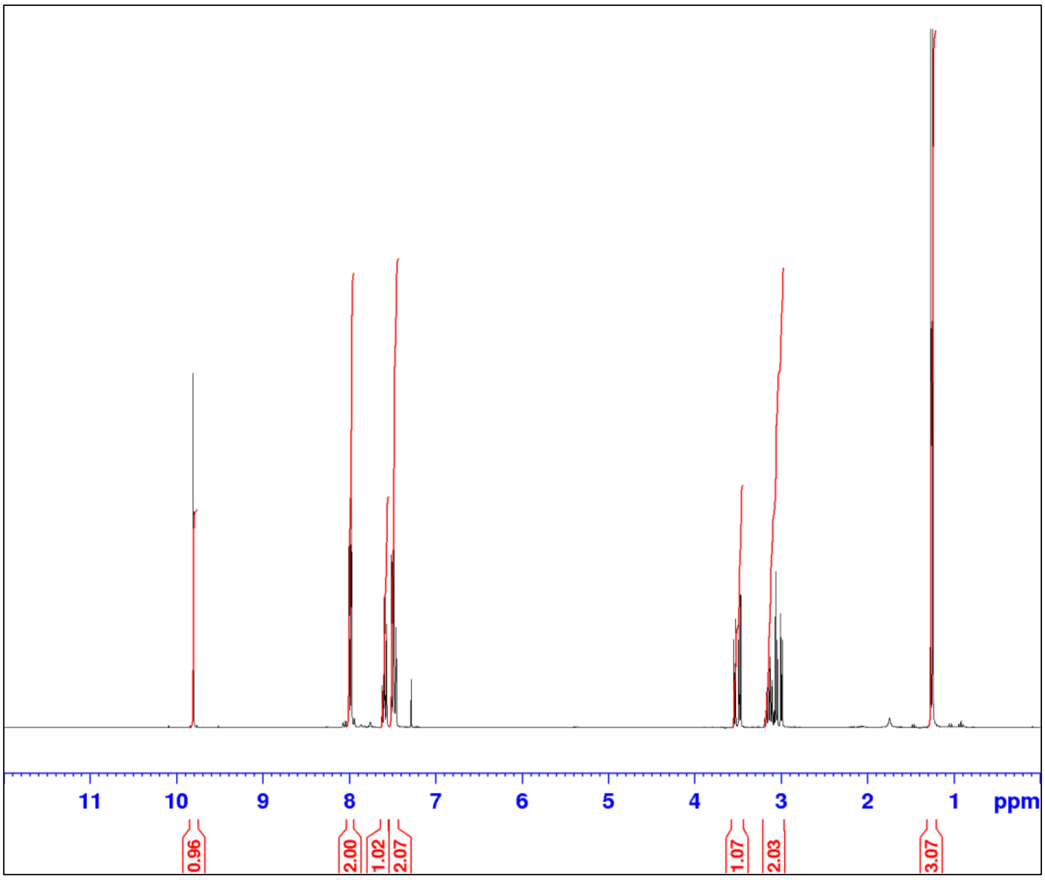

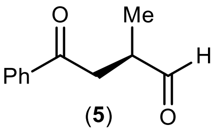

(**5**)

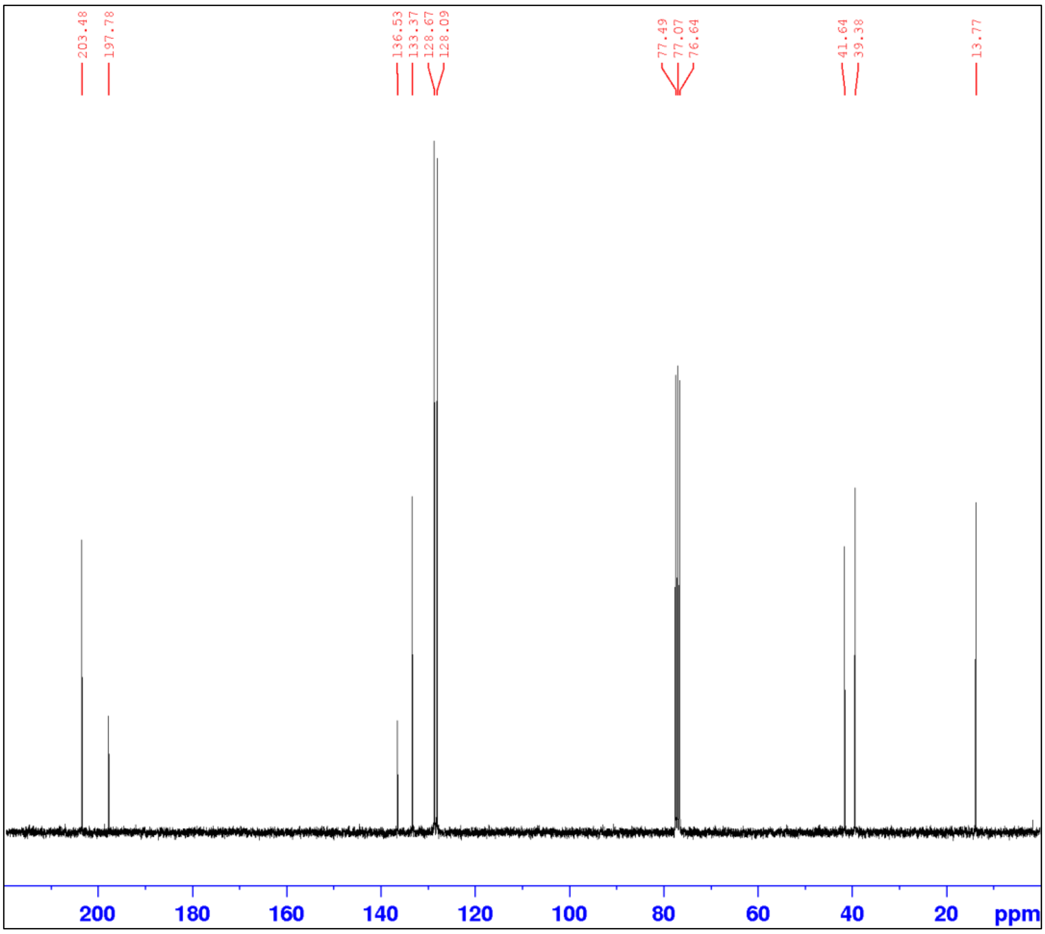

75 MHz, $CDCl_3$

HPLC chromatograms

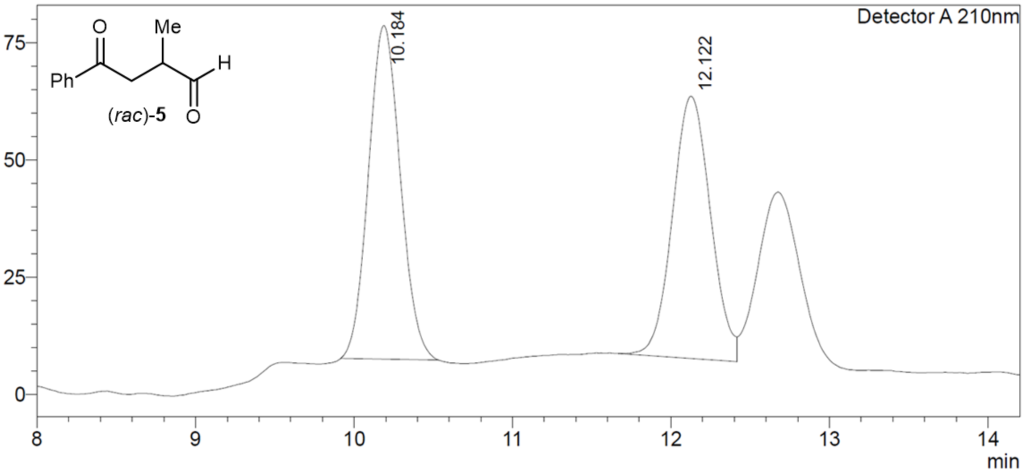

Peak#	Ret. Time	Area	Height	Area%
1	10.184	995159	71073	51.592
2	12.122	933740	55907	48.408
Total		1928900	126979	100.000

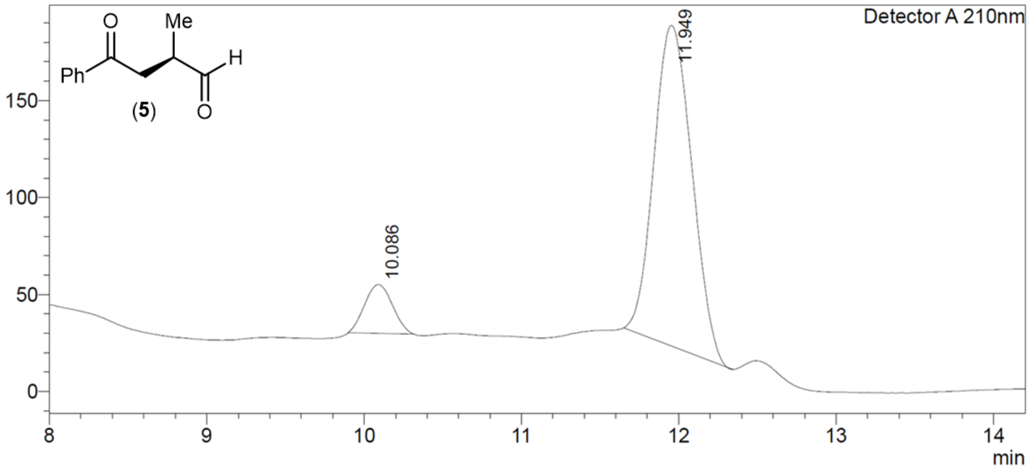

Peak#	Ret. Time	Area	Height	Area%
1	10.086	309656	25190	10.035
2	11.949	2776054	165168	89.965
Total		3085710	190358	100.000

300 MHz, $CDCl_3$

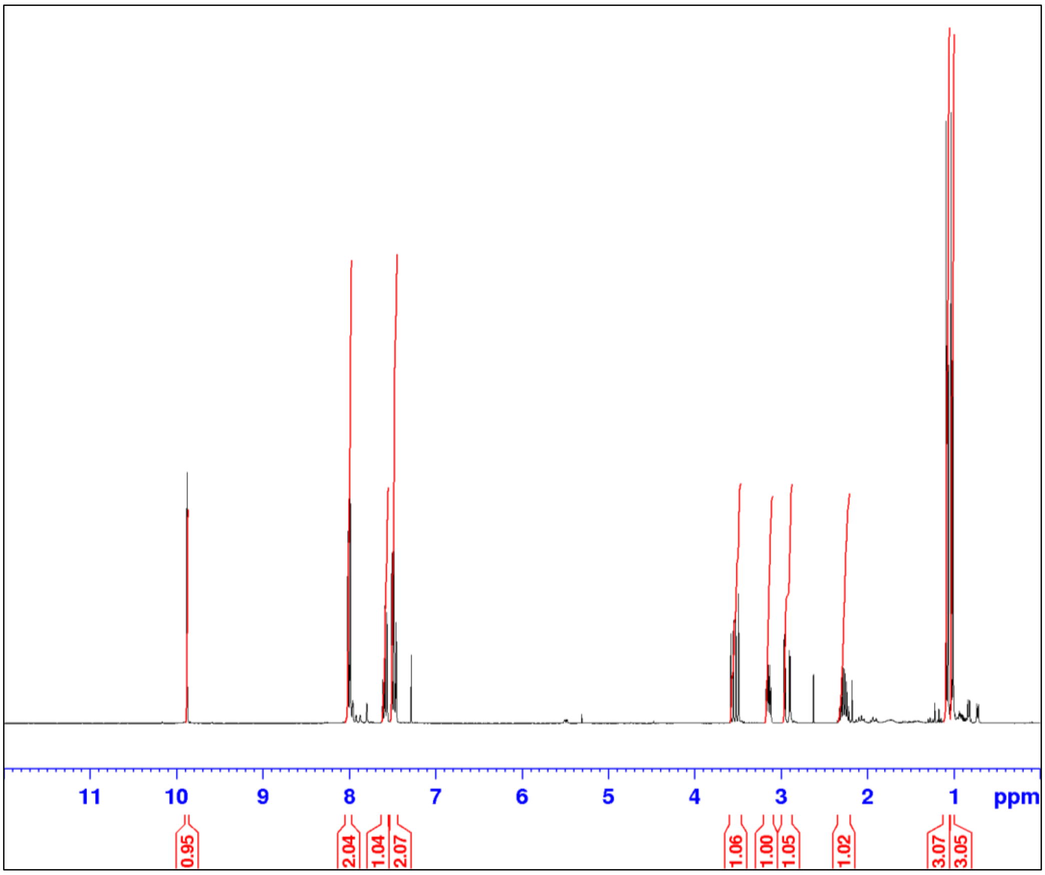

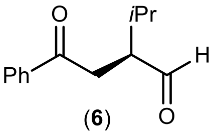

(**6**)

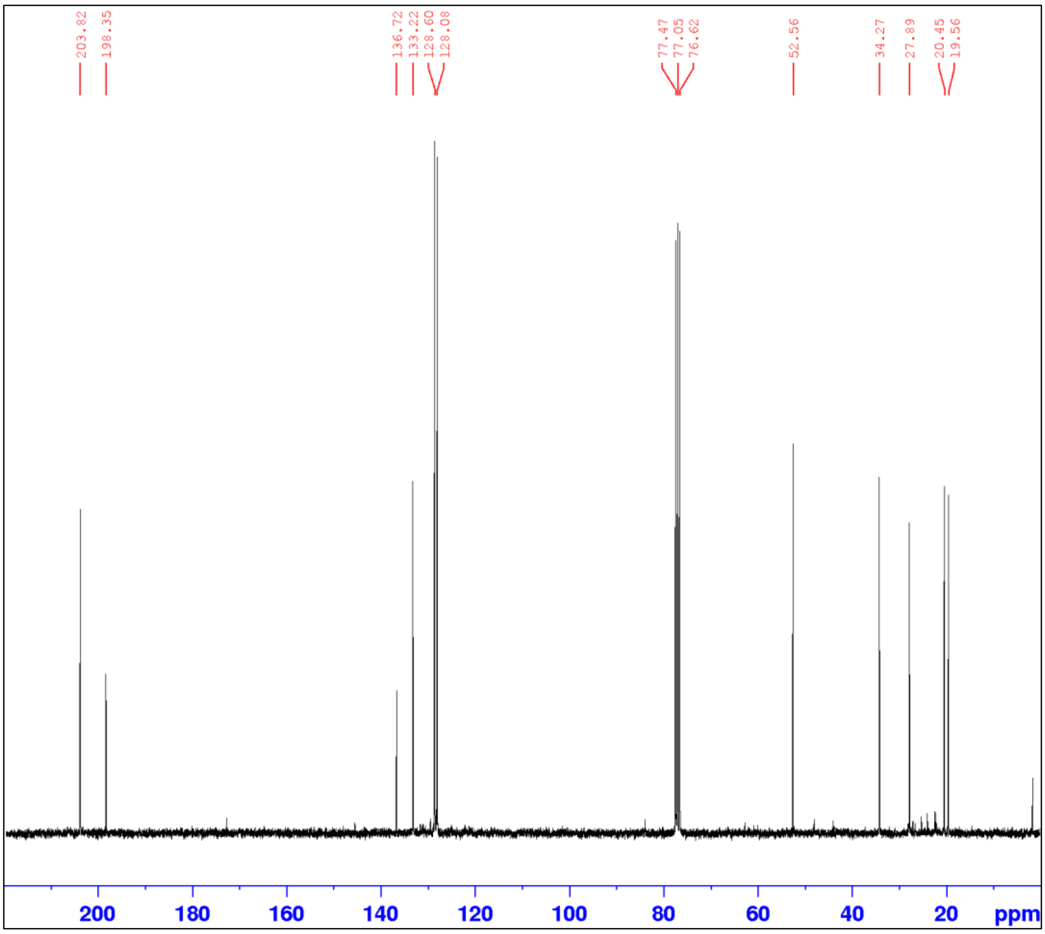

75 MHz, $CDCl_3$

HPLC chromatograms

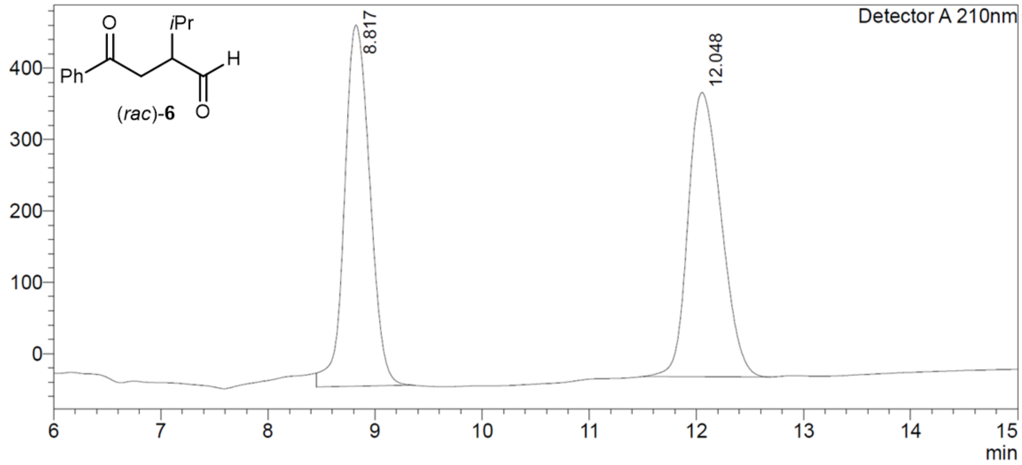

Peak#	Ret. Time	Area	Height	Area%
1	8.817	8344064	505900	49.305
2	12.048	8579353	398169	50.695
Total		16923417	904070	100.000

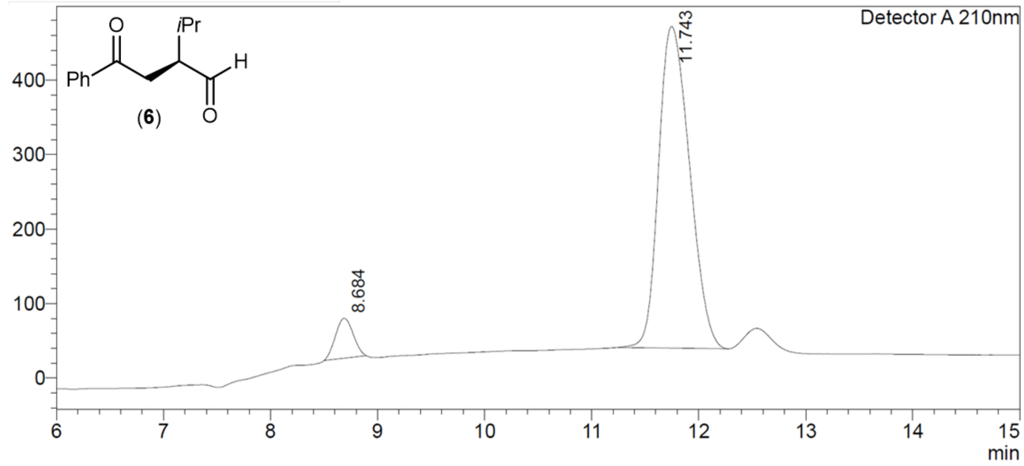

Peak#	Ret. Time	Area	Height	Area%
1	8.684	618864	53634	6.661
2	11.743	8672430	431476	93.339
Total		9291294	485111	100.000

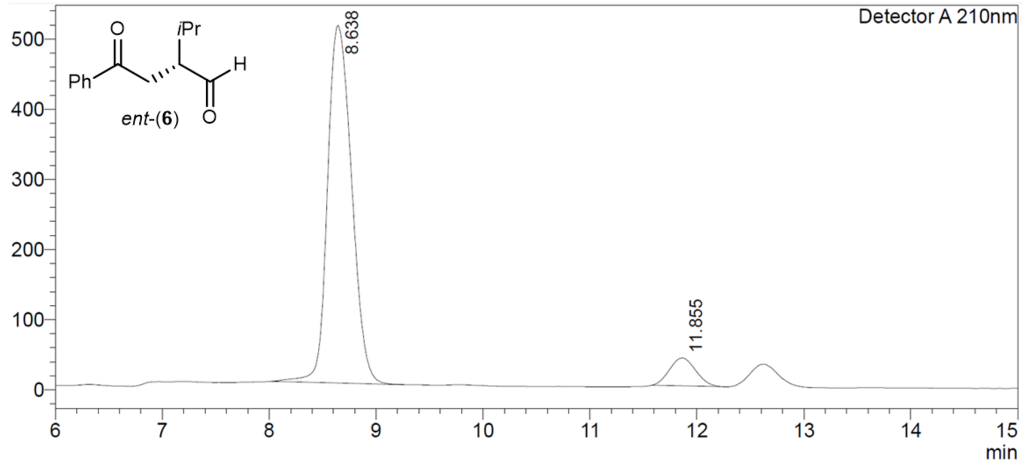

Peak#	Ret. Time	Area	Height	Area%
1	8.638	8000616	509587	92.266
2	11.855	670668	39619	7.734
Total		8671285	549206	100.000

300 MHz, CDCl3

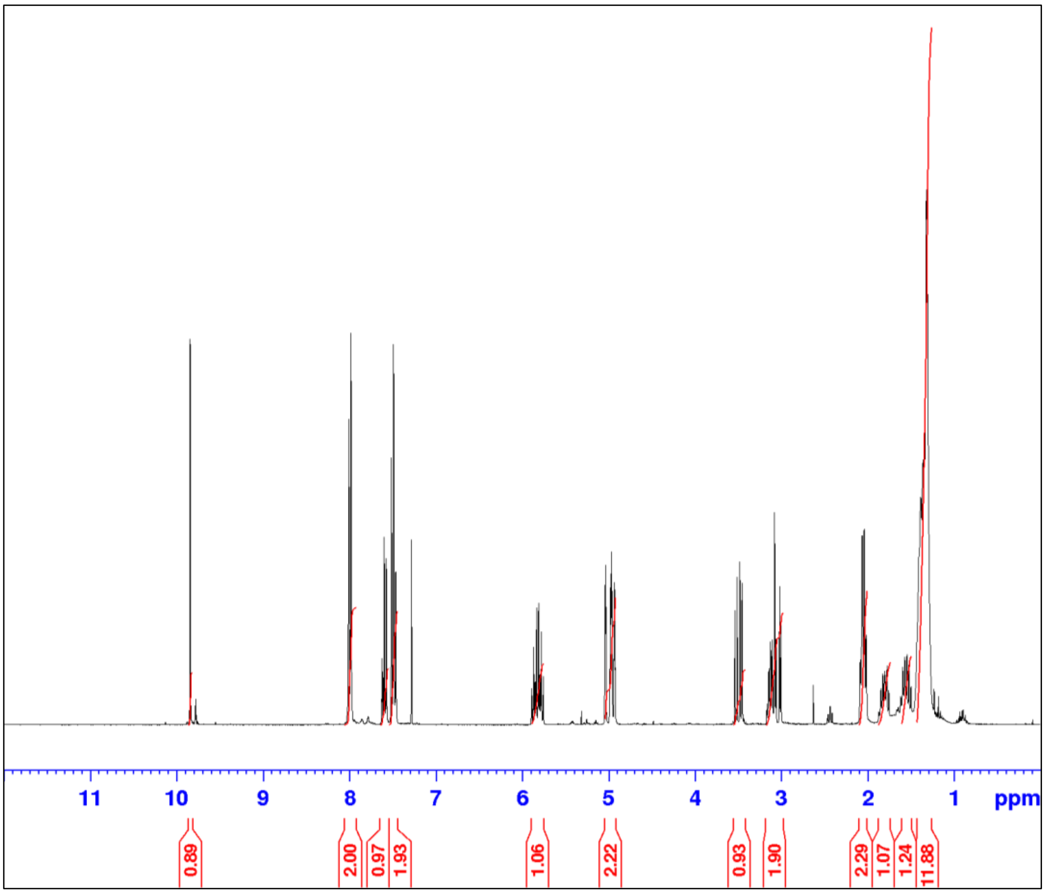

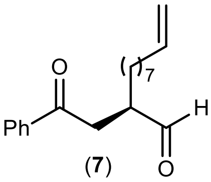

(**7**)

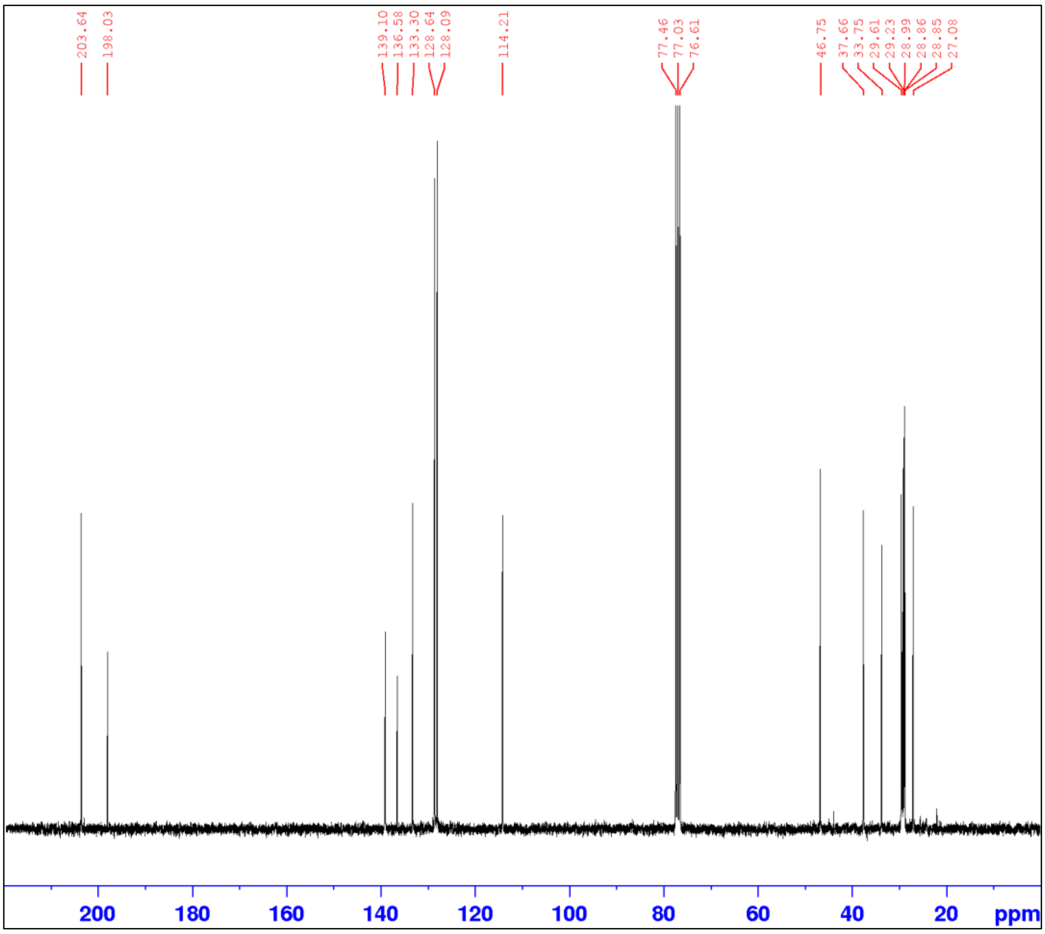

75 MHz, CDCl3

HPLC chromatograms

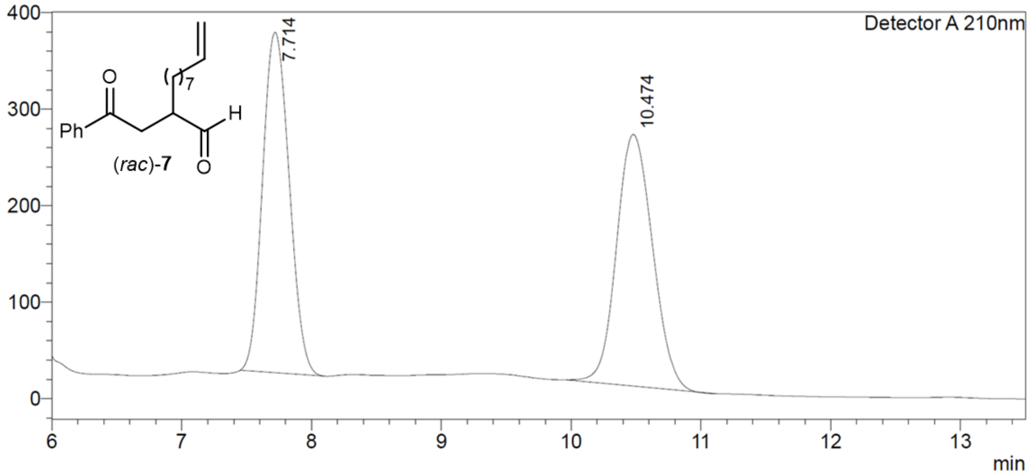

Peak#	Ret. Time	Area	Height	Area%
1	7.714	5028163	352771	49.934
2	10.474	5041516	260738	50.066
Total		10069679	613509	100.000

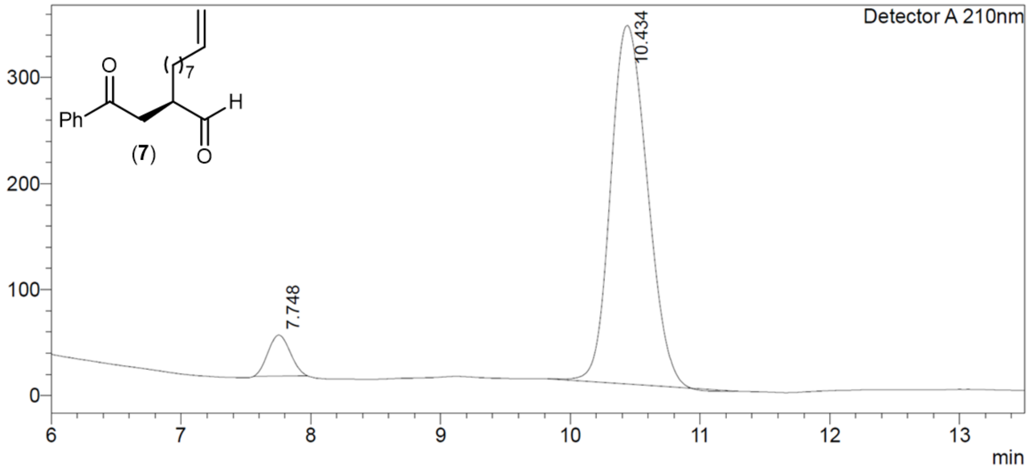

Peak#	Ret. Time	Area	Height	Area%
1	7.748	459214	38726	6.466
2	10.434	6642744	338348	93.534
Total		7101958	377074	100.000

300 MHz, $CDCl_3$

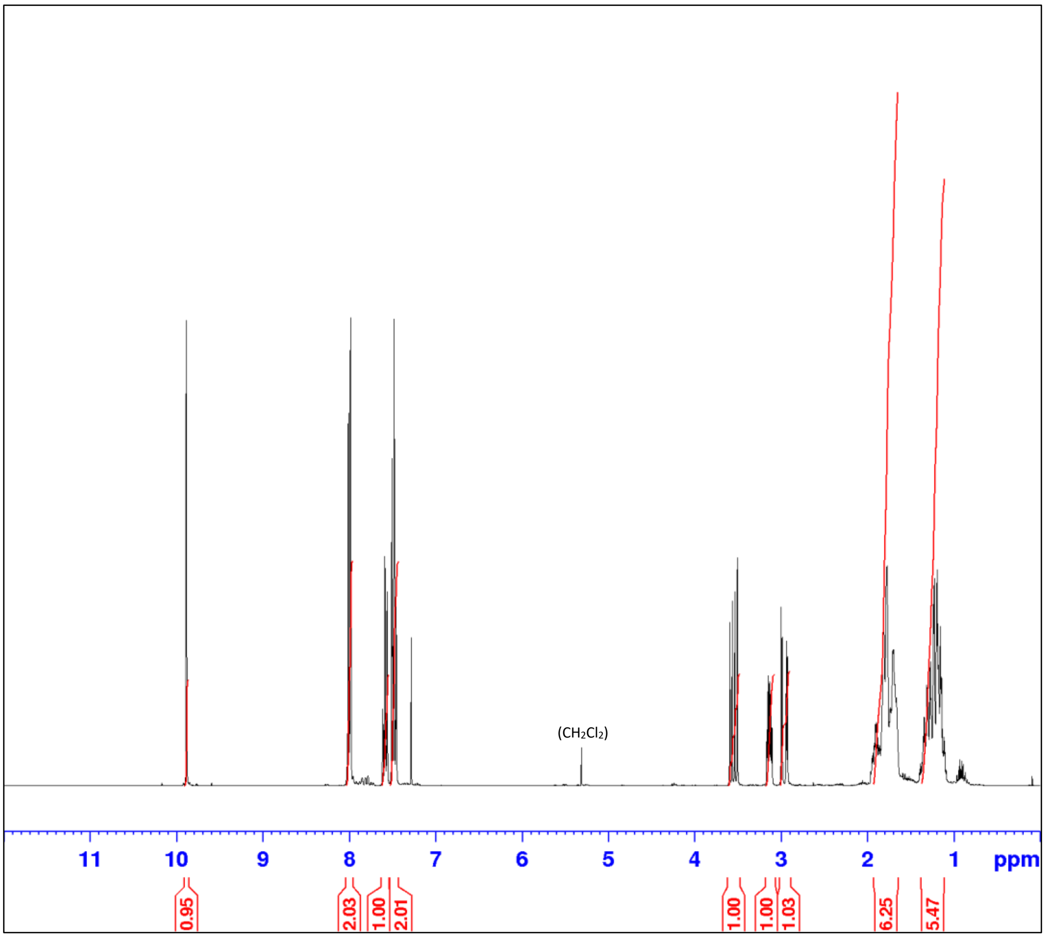

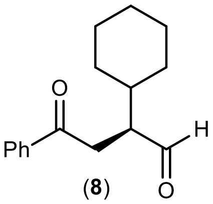

(**8**)

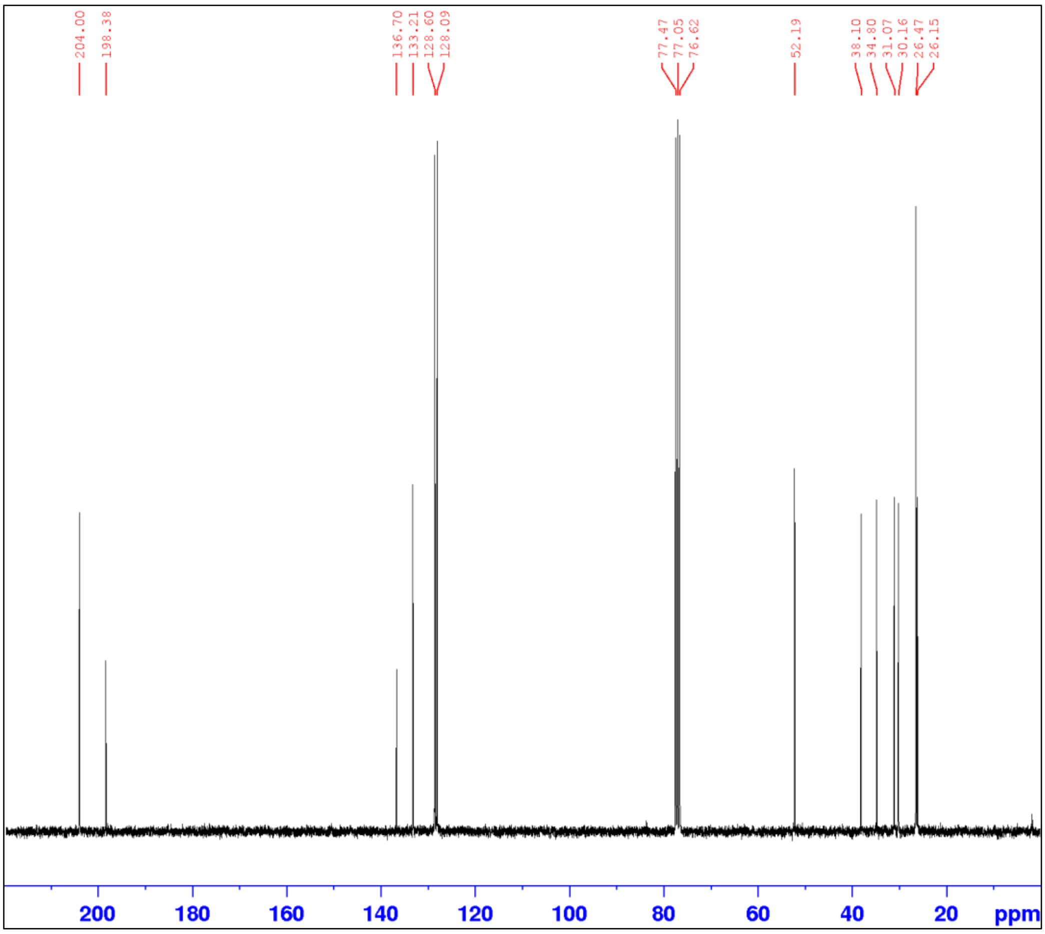

75 MHz, $CDCl_3$

HPLC chromatograms

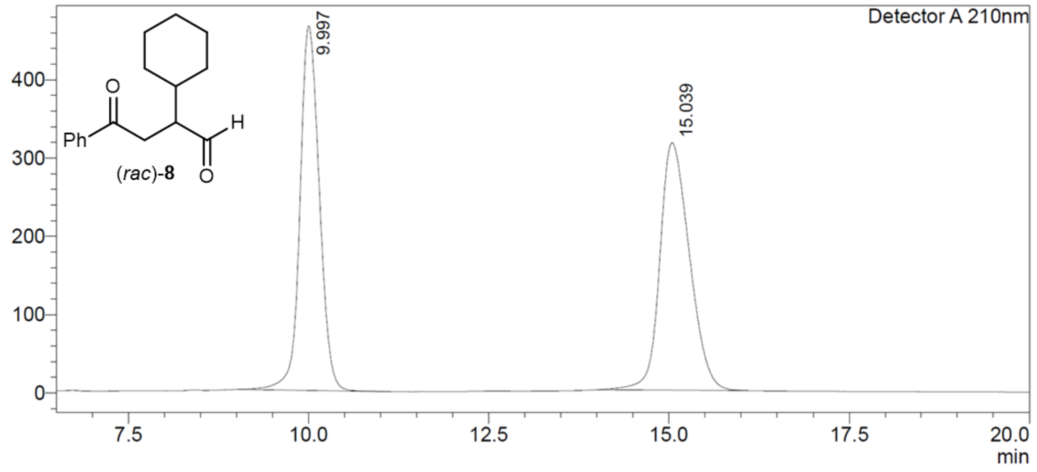

Peak#	Ret. Time	Area	Height	Area%
1	9.997	8746694	465616	49.144
2	15.039	9051262	315984	50.856
Total		17797957	781600	100.000

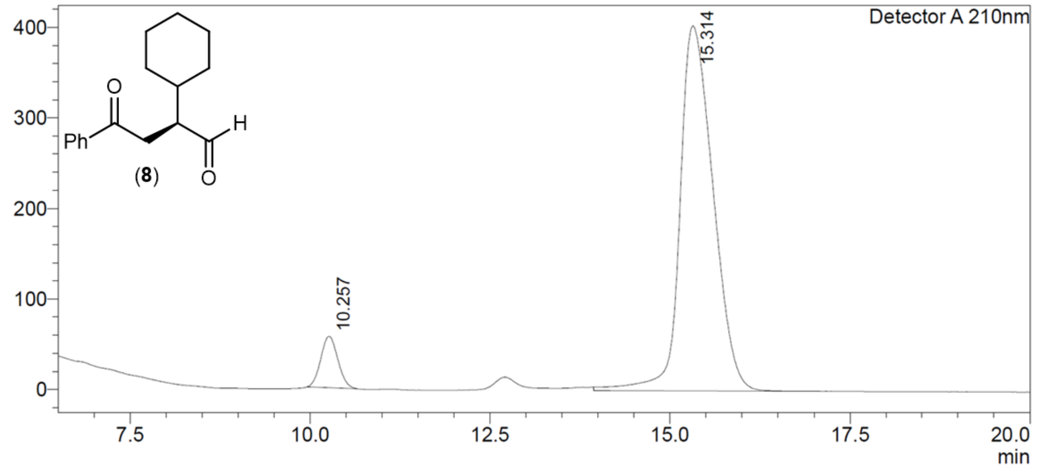

Peak#	Ret. Time	Area	Height	Area%
1	10.257	935519	56760	6.724
2	15.314	12977875	403355	93.276
Total		13913393	460115	100.000

300 MHz, $CDCl_3$

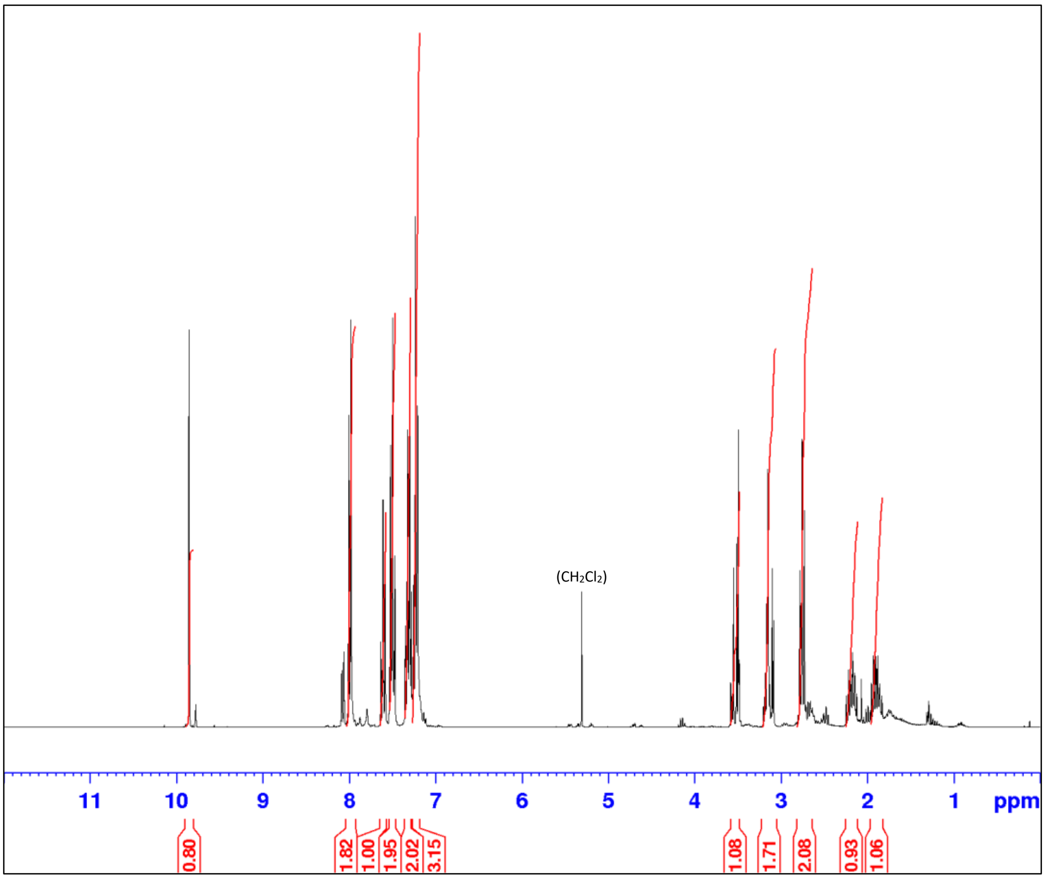

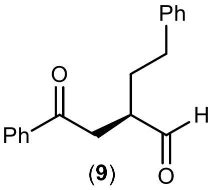

(**9**)

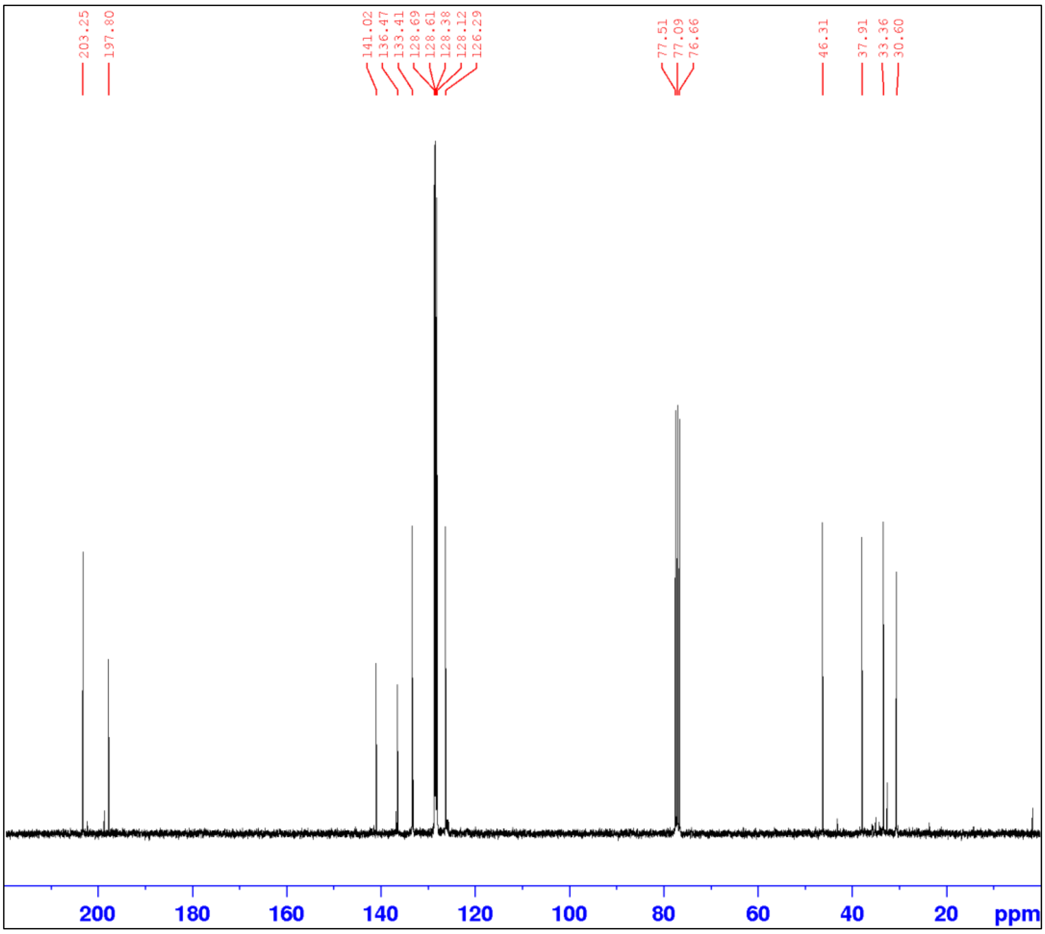

75 MHz, $CDCl_3$

HPLC chromatograms

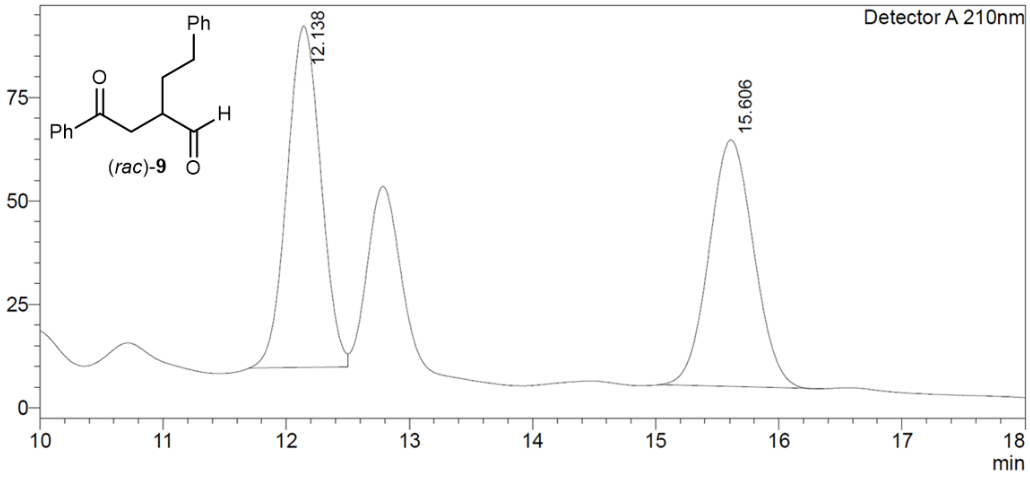

Peak#	Ret. Time	Area	Height	Area%
1	12.138	1578643	82574	51.193
2	15.606	1505074	59650	48.807
Total		3083717	142224	100.000

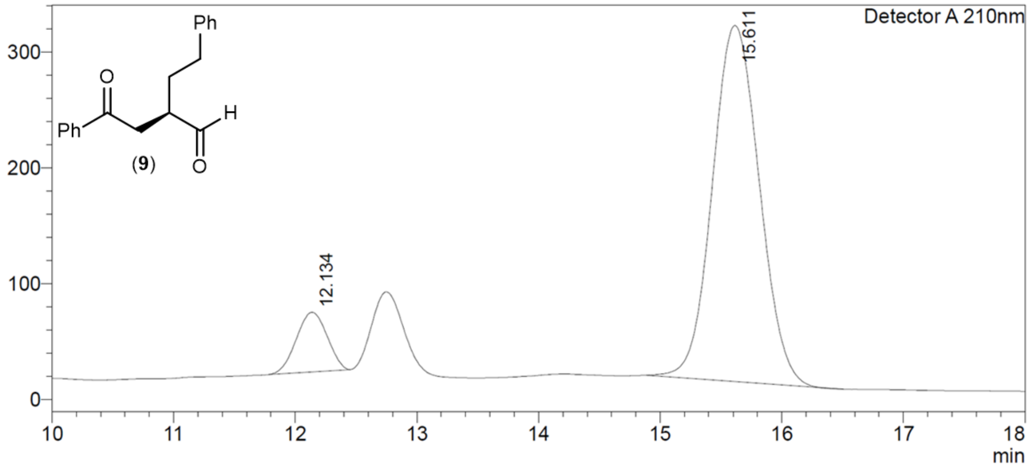

Peak#	Ret. Time	Area	Height	Area%
1	12.134	829651	49232	9.137
2	15.611	8250096	307218	90.863
Total		9079747	356450	100.000

300 MHz, $CDCl_3$

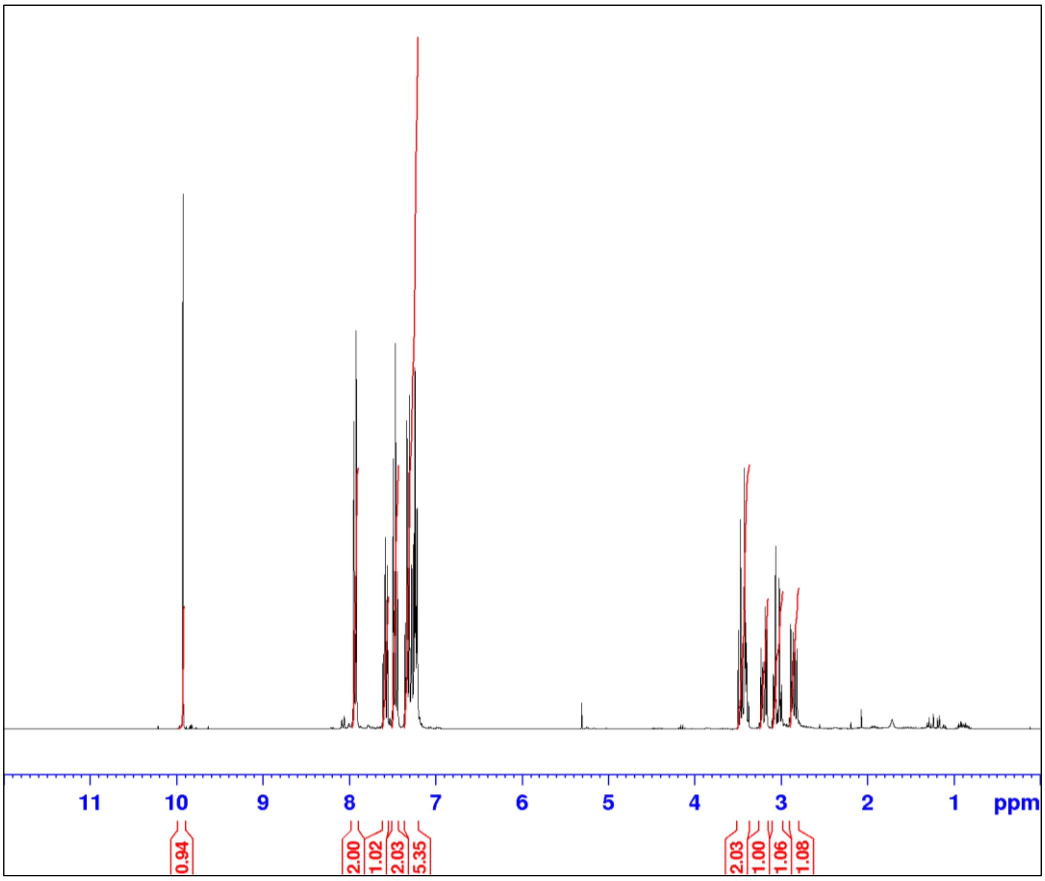

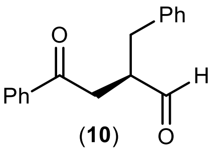

(**10**)

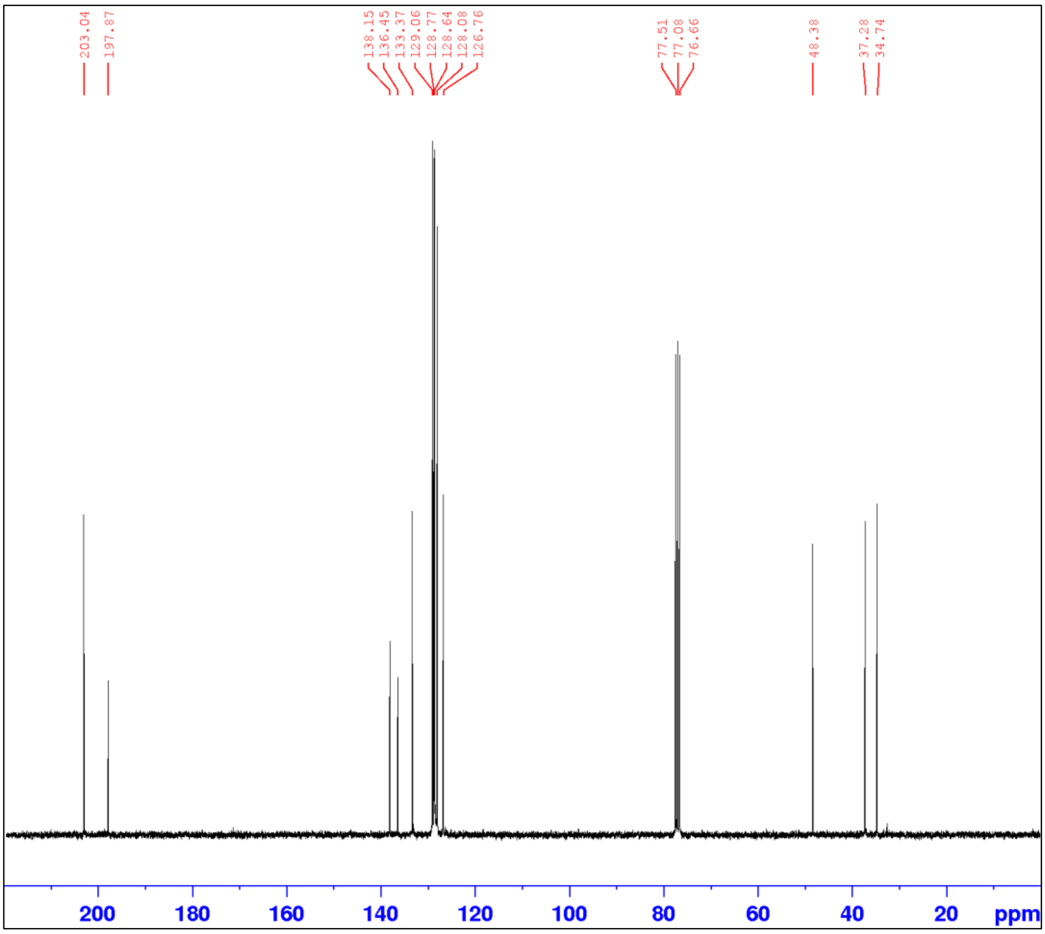

75 MHz, $CDCl_3$

HPLC chromatograms

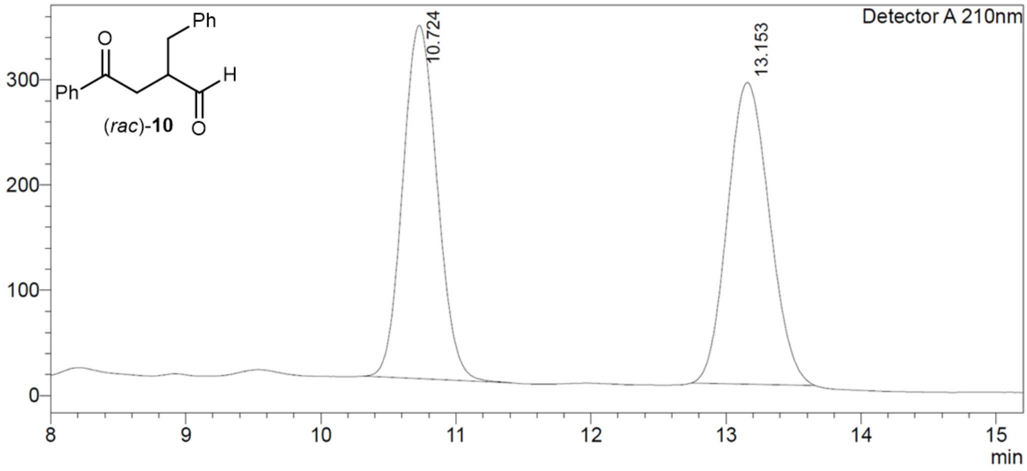

Peak#	Ret. Time	Area	Height	Area%
1	10.724	5952358	335688	49.006
2	13.153	6193941	286732	50.994
Total		12146298	622421	100.000

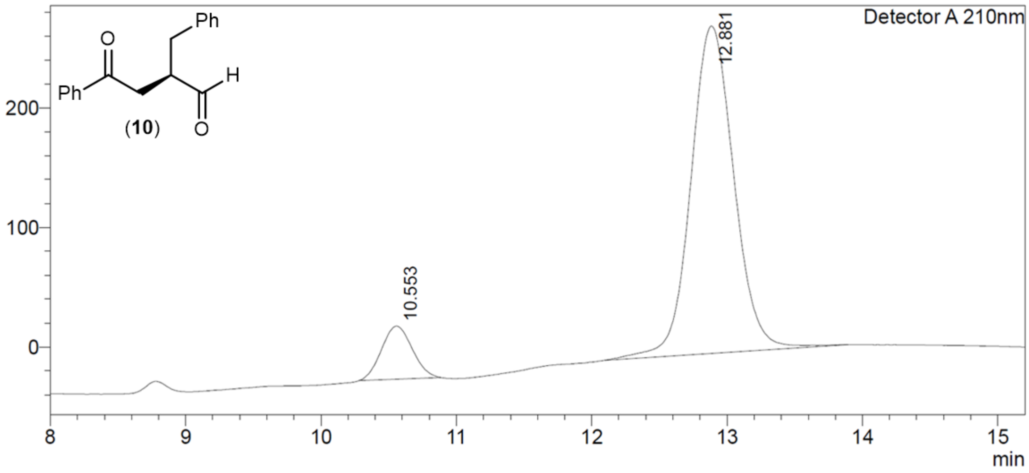

Peak#	Ret. Time	Area	Height	Area%
1	10.553	647176	42774	9.525
2	12.881	6147556	274662	90.475
Total		6794732	317436	100.000

300 MHz, $CDCl_3$

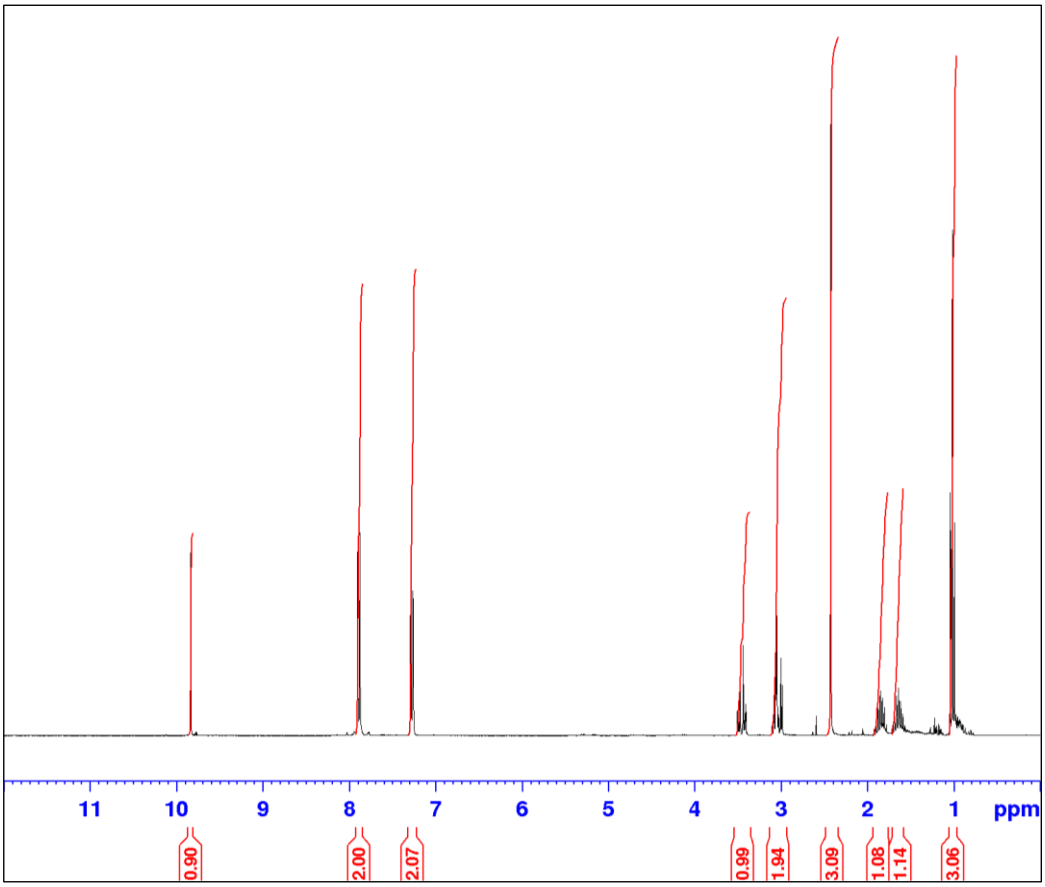

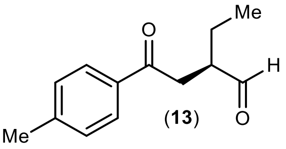

(**13**)

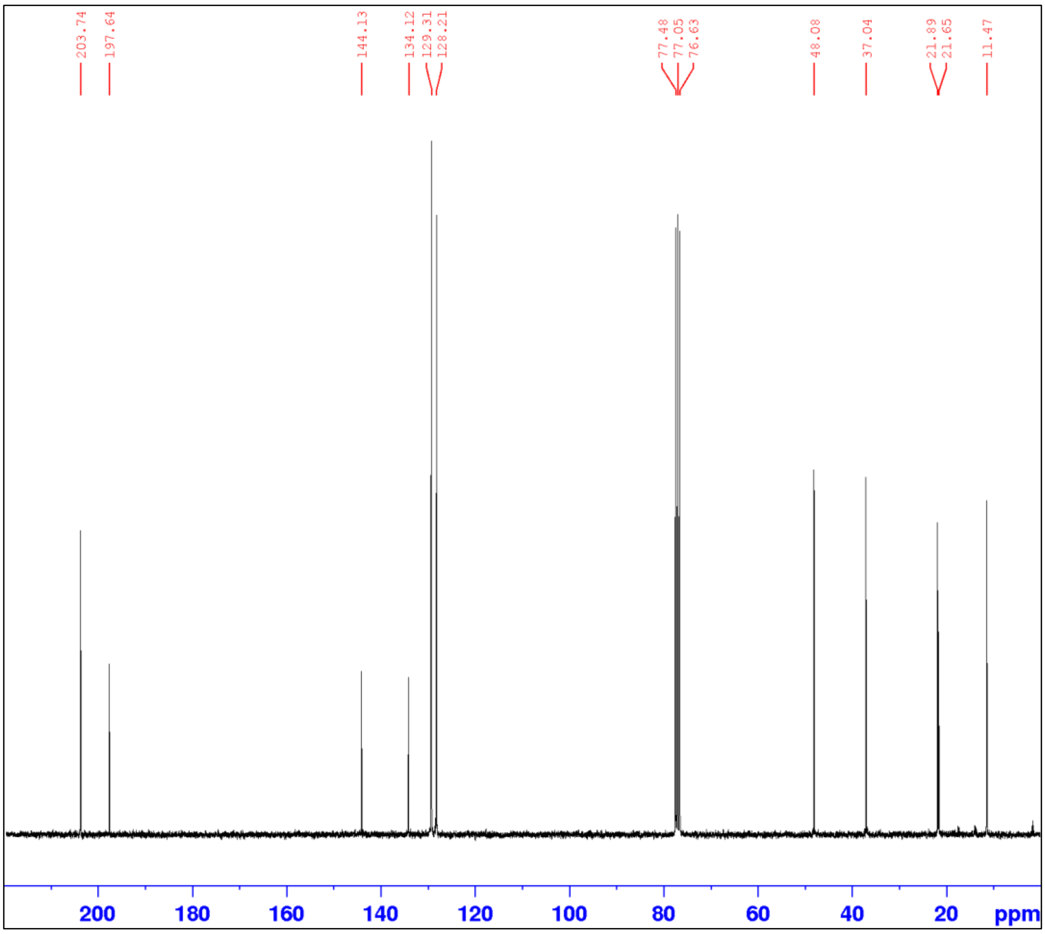

75 MHz, $CDCl_3$

HPLC chromatograms

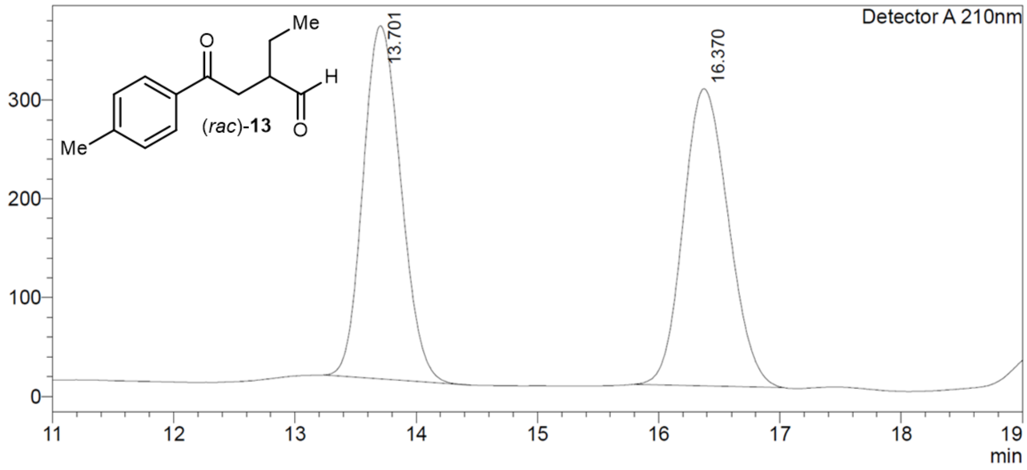

Peak#	Ret. Time	Area	Height	Area%
1	13.701	7765785	357105	49.840
2	16.370	7815568	300600	50.160
Total		15581352	657705	100.000

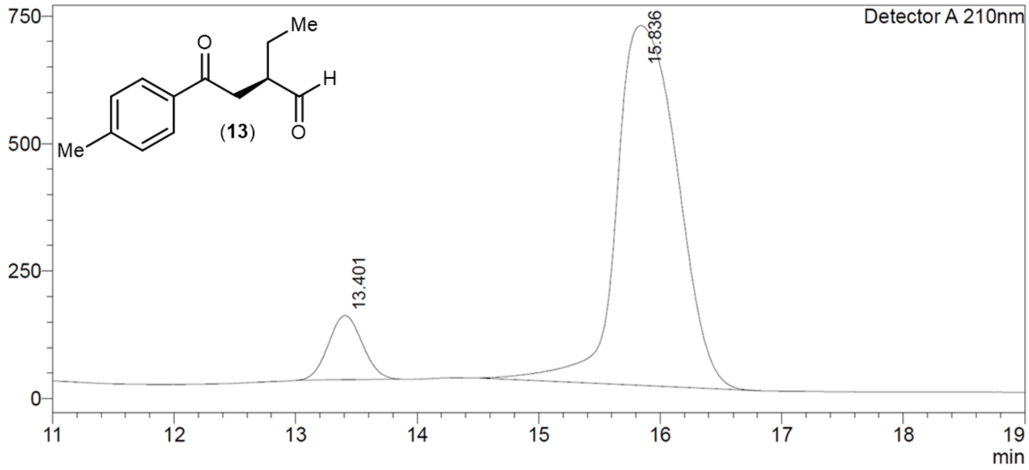

Peak#	Ret. Time	Area	Height	Area%
1	13.401	2446220	125979	8.989
2	15.836	24766679	705459	91.011
Total		27212900	831438	100.000

300 MHz, CDCl$_3$

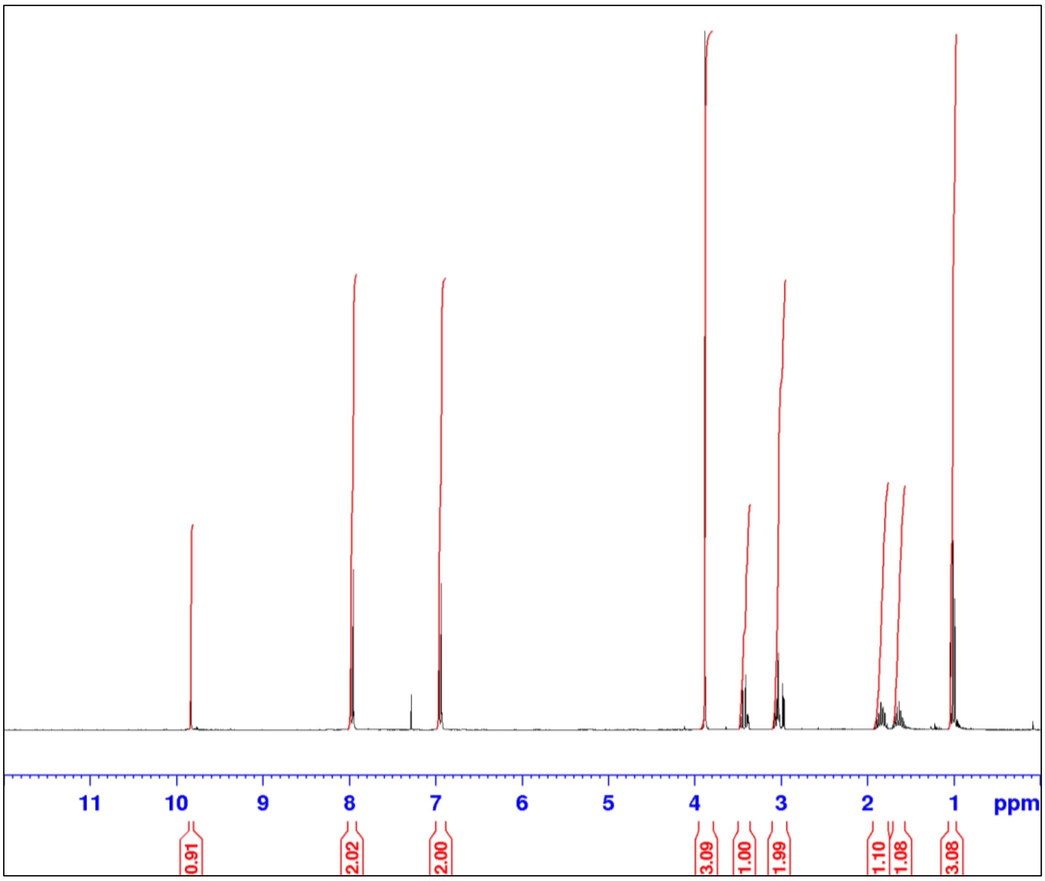

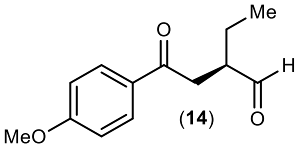

(**14**)

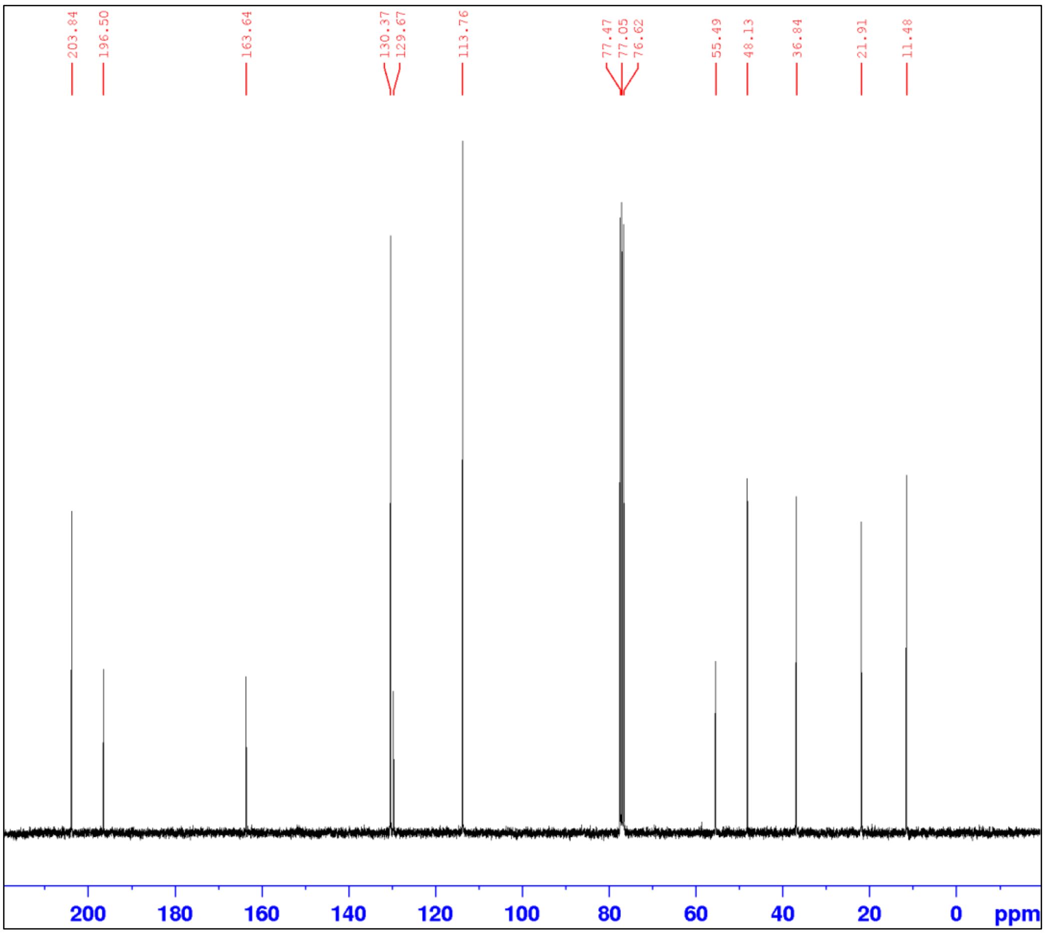

75 MHz, CDCl$_3$

HPLC chromatograms

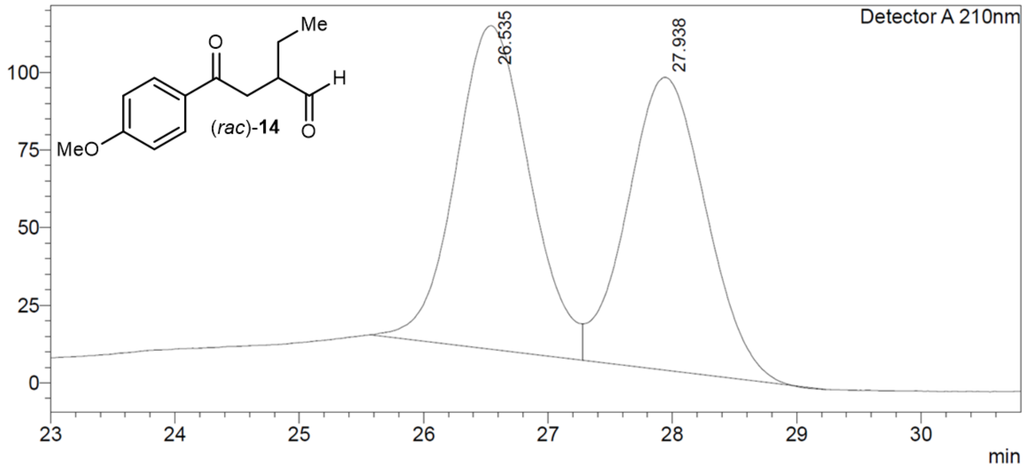

Peak#	Ret. Time	Area	Height	Area%
1	26.535	4337543	104193	51.025
2	27.938	4163272	94280	48.975
Total		8500814	198473	100.000

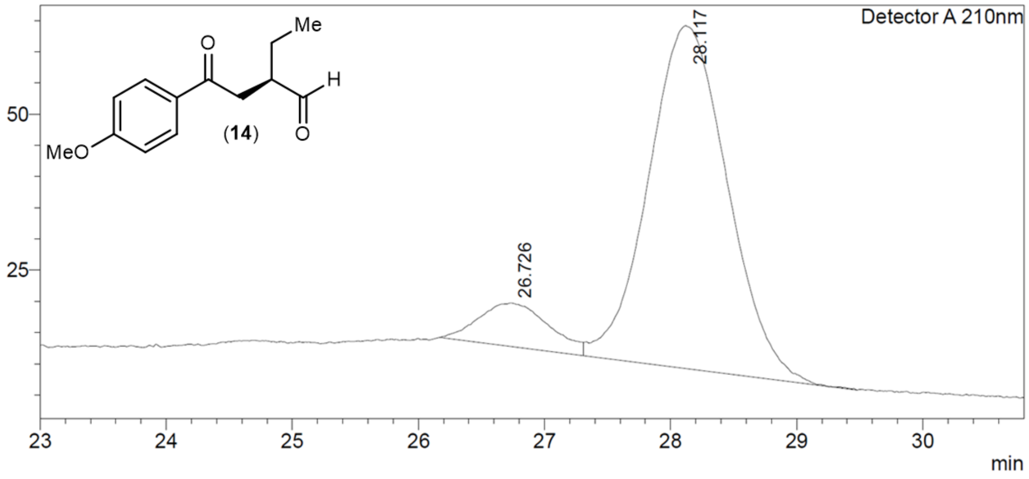

Peak#	Ret. Time	Area	Height	Area%
1	26.726	232293	6335	8.988
2	28.117	2352164	54518	91.012
Total		2584457	60853	100.000

300 MHz, $CDCl_3$

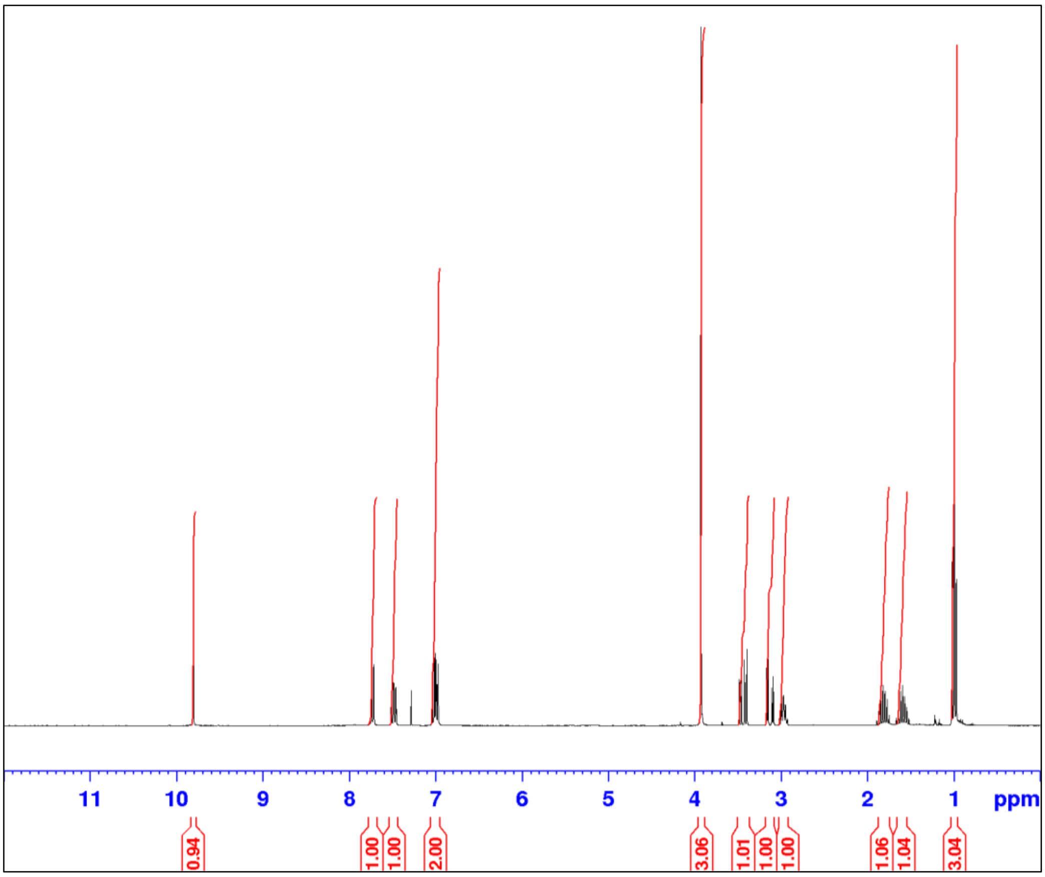

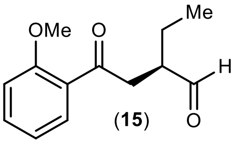

(**15**)

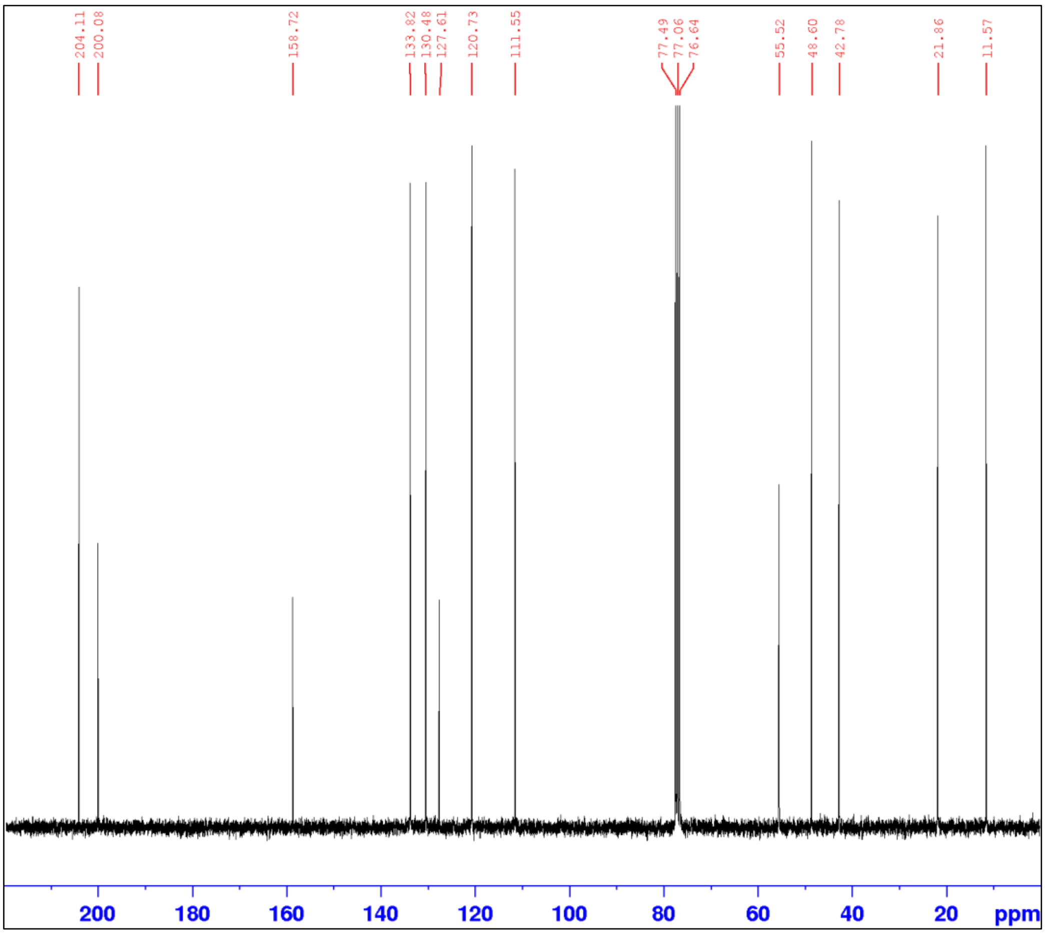

75 MHz, $CDCl_3$

HPLC chromatograms

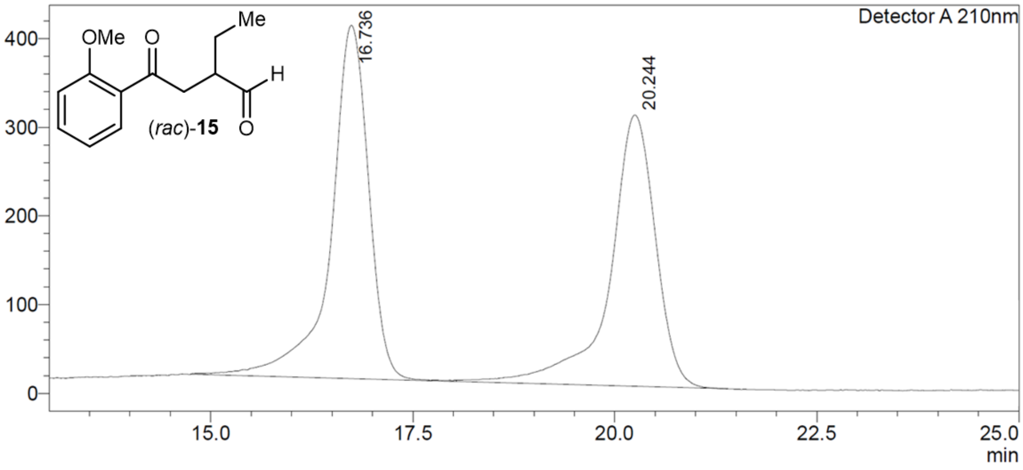

Peak#	Ret. Time	Area	Height	Area%
1	16.736	13028942	398362	51.760
2	20.244	12143080	306105	48.240
Total		25172022	704466	100.000

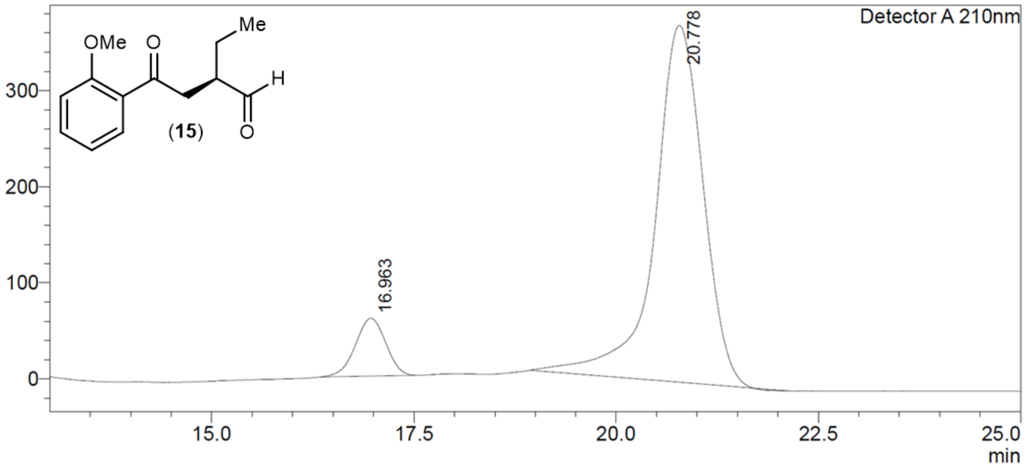

Peak#	Ret. Time	Area	Height	Area%
1	16.963	1572417	60276	9.222
2	20.778	15479047	371277	90.778
Total		17051465	431554	100.000

300 MHz, $CDCl_3$

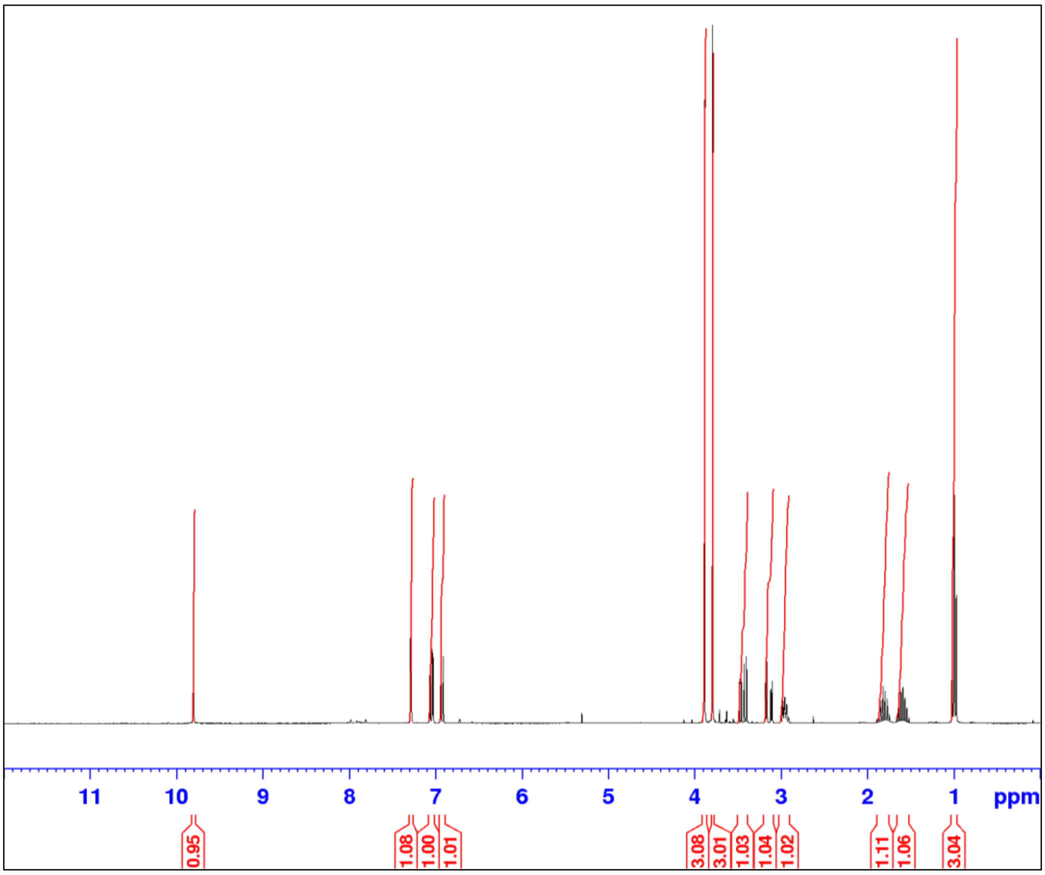

(**16**)

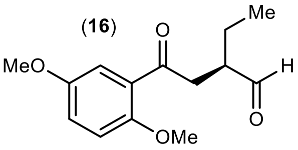

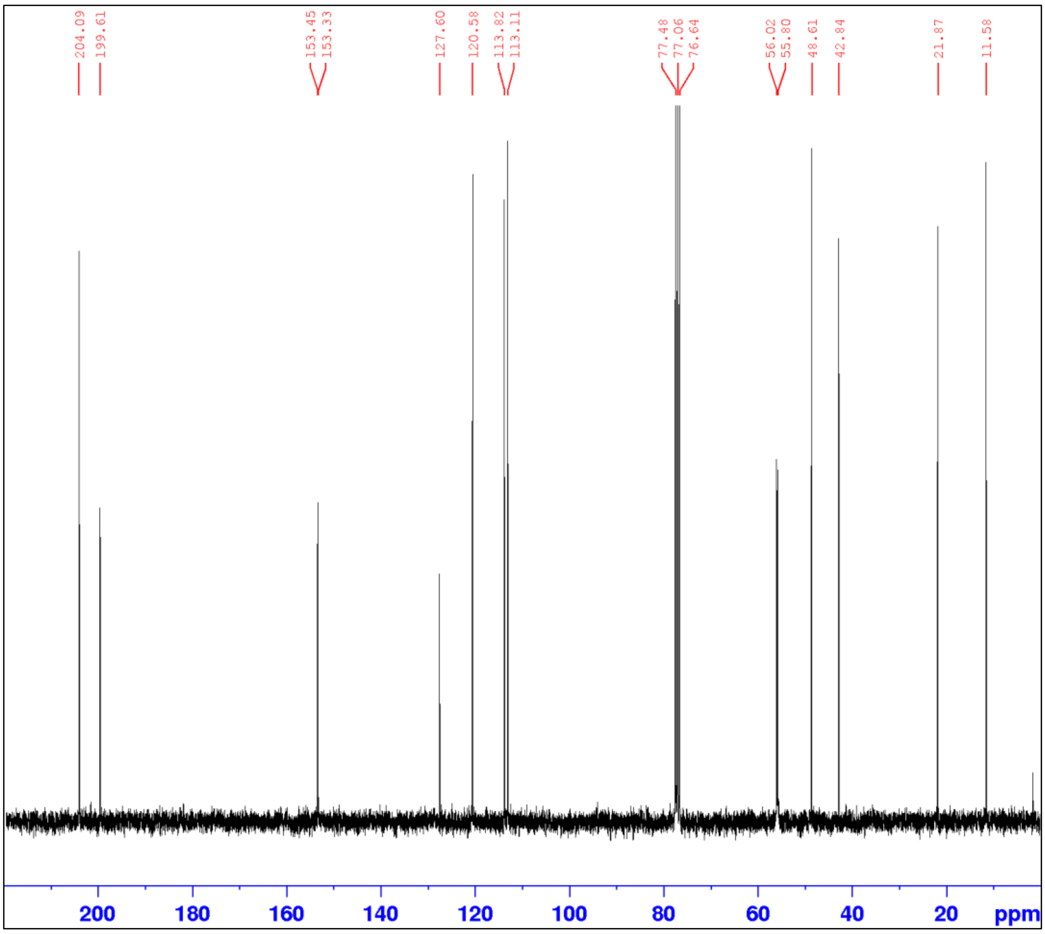

75 MHz, $CDCl_3$

HPLC chromatograms

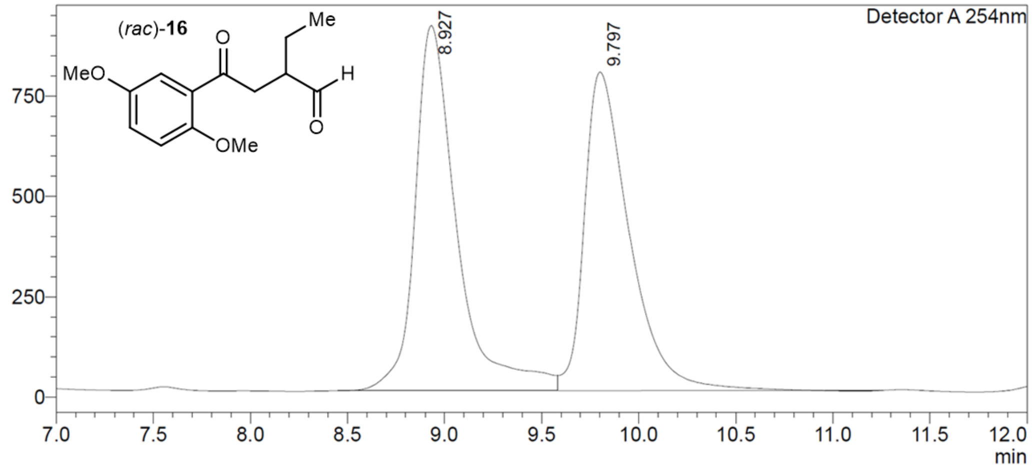

Peak#	Ret. Time	Area	Height	Area%
1	8.927	13665422	909045	51.700
2	9.797	12766688	793008	48.300
Total		26432110	1702052	100.000

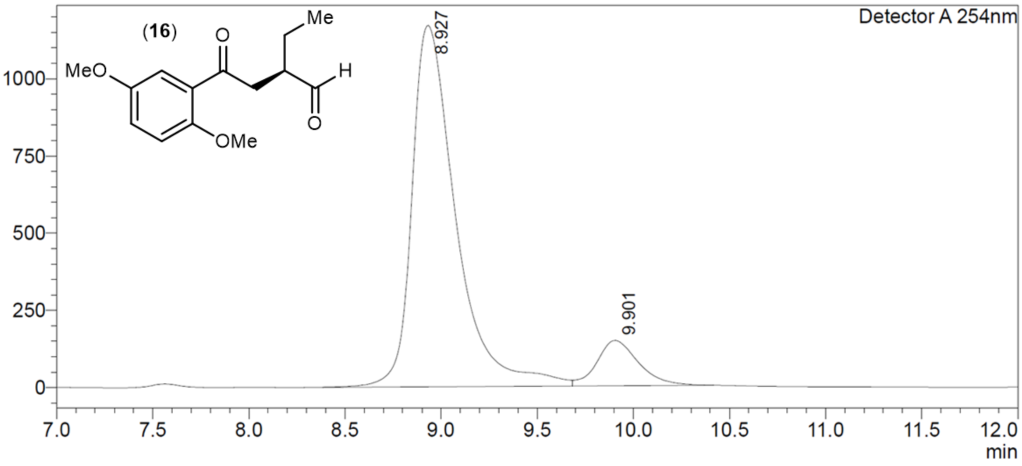

Peak#	Ret. Time	Area	Height	Area%
1	8.927	18261115	1166370	90.528
2	9.901	1910704	138528	9.472
Total		20171820	1304897	100.000

300 MHz, $CDCl_3$

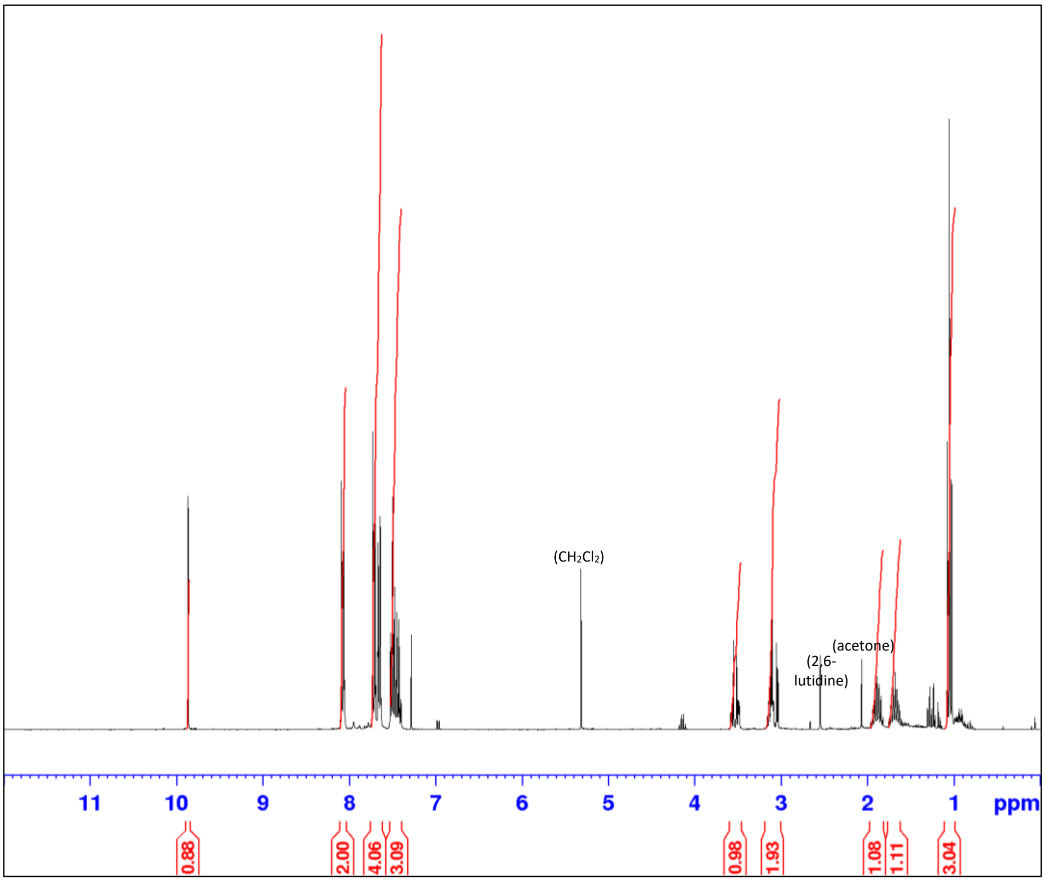

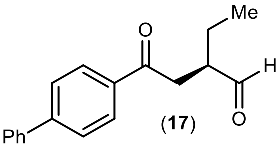

(17)

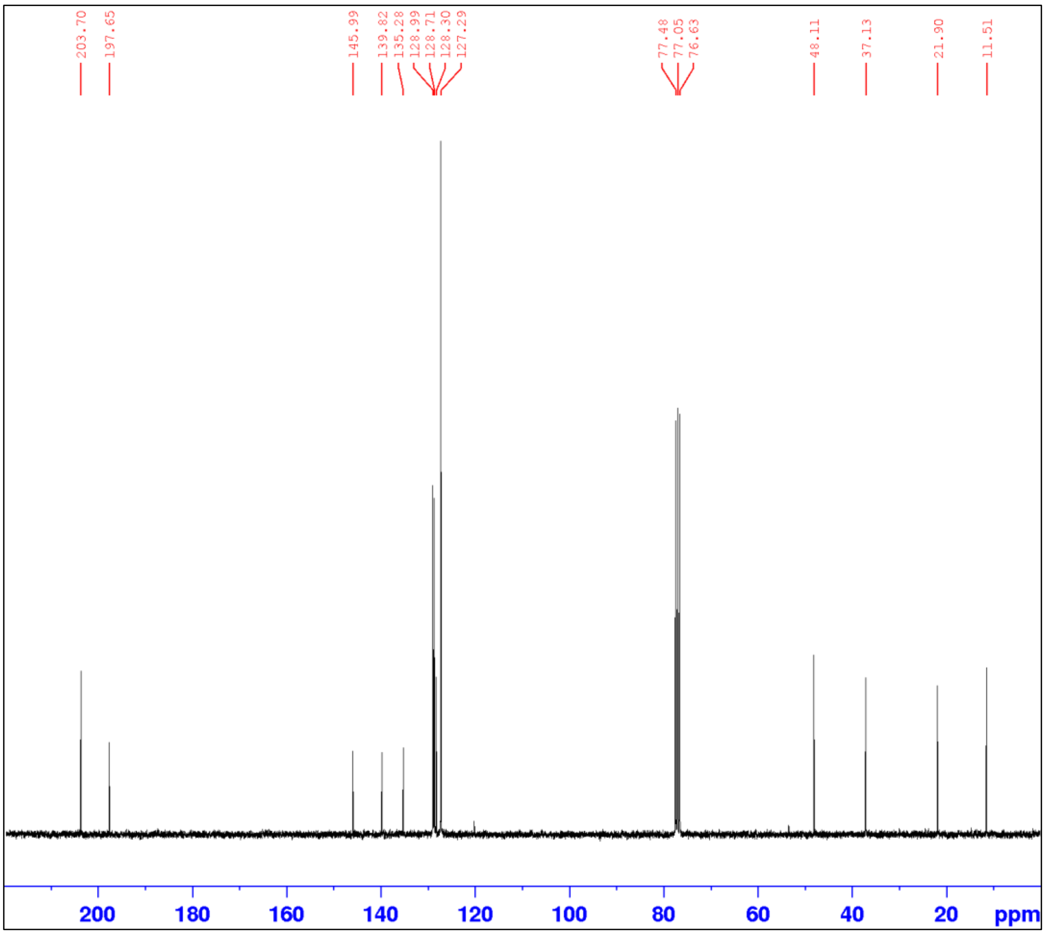

75 MHz, $CDCl_3$

HPLC chromatograms

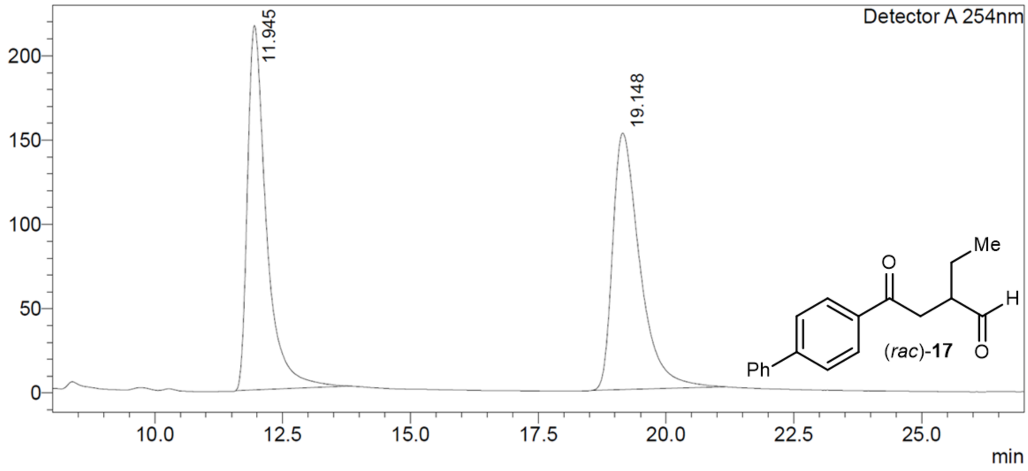

Peak#	Ret. Time	Area	Height	Area%
1	11.945	5557645	216007	49.616
2	19.148	5643763	152069	50.384
Total		11201409	368076	100.000

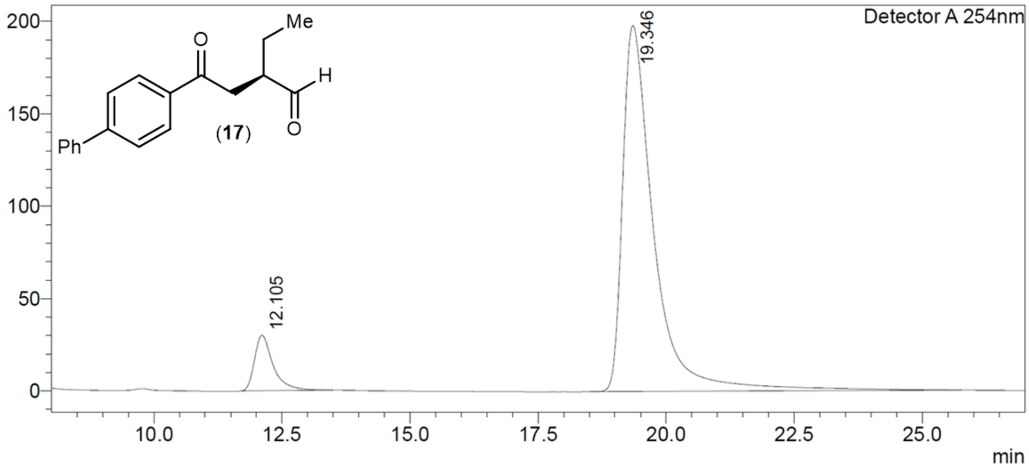

Peak#	Ret. Time	Area	Height	Area%
1	12.105	755368	29968	8.239
2	19.346	8412655	198116	91.761
Total		9168024	228084	100.000

300 MHz, $CDCl_3$

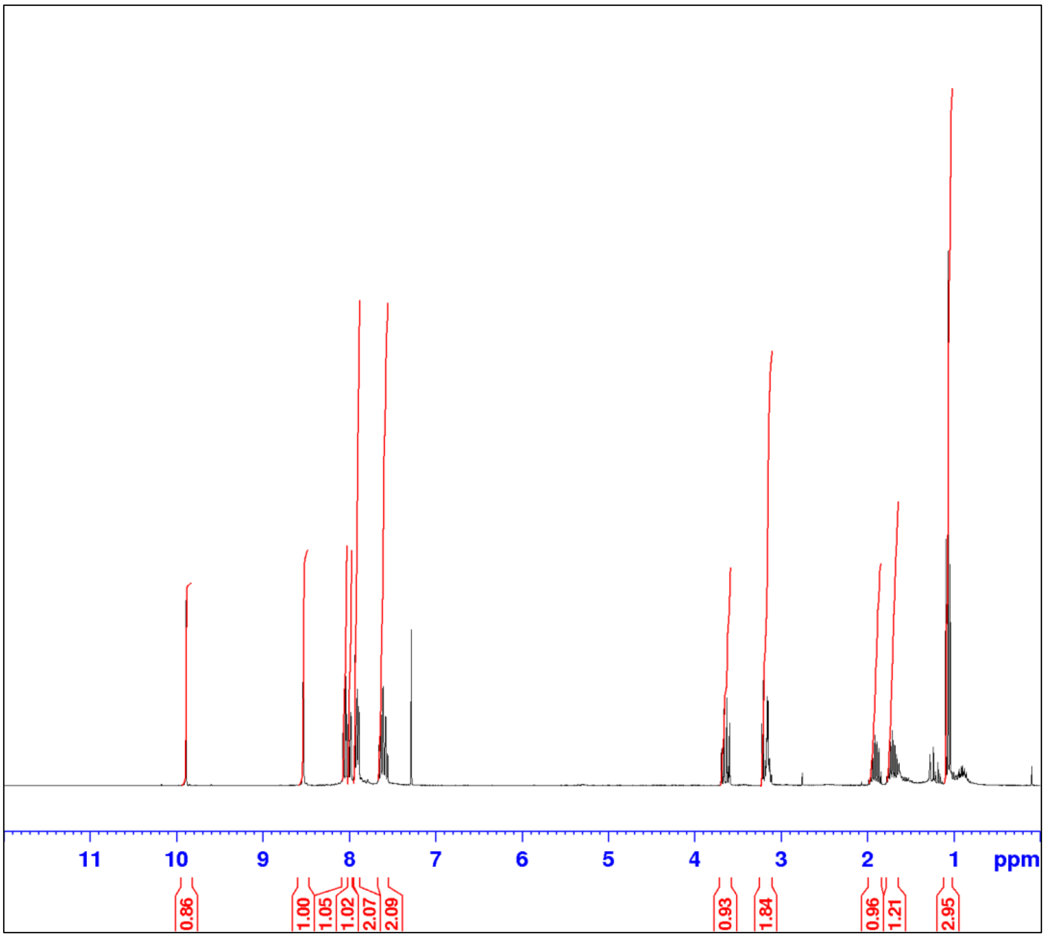

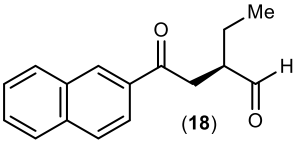

(**18**)

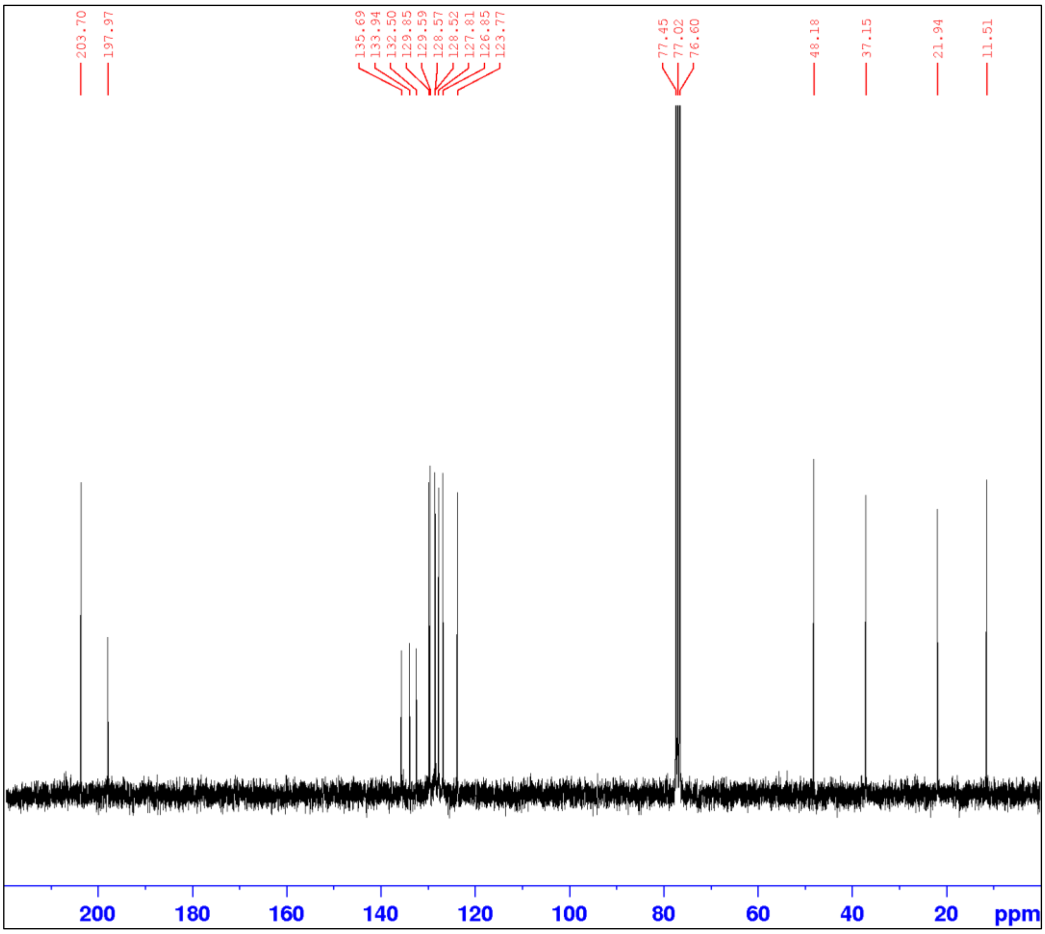

75 MHz, $CDCl_3$

HPLC chromatograms

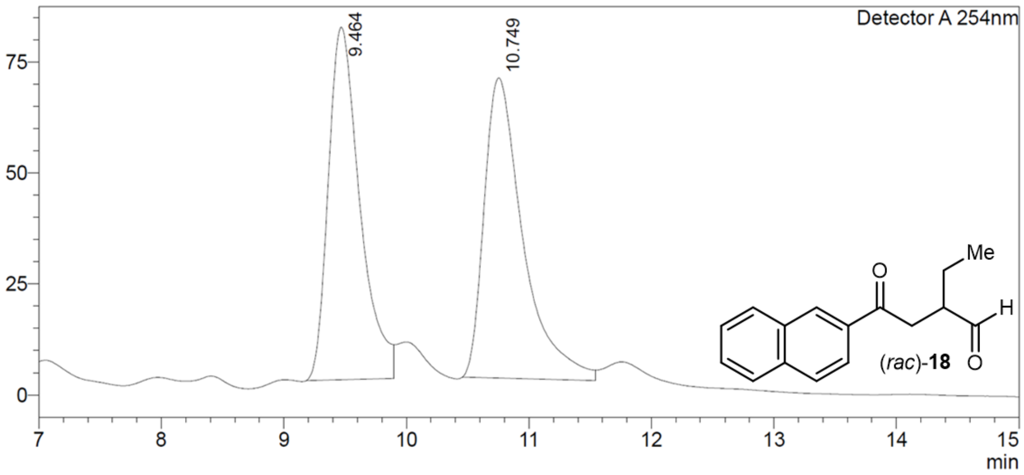

Peak#	Ret. Time	Area	Height	Area%
1	9.464	1426118	79466	48.826
2	10.749	1494711	67619	51.174
Total		2920830	147085	100.000

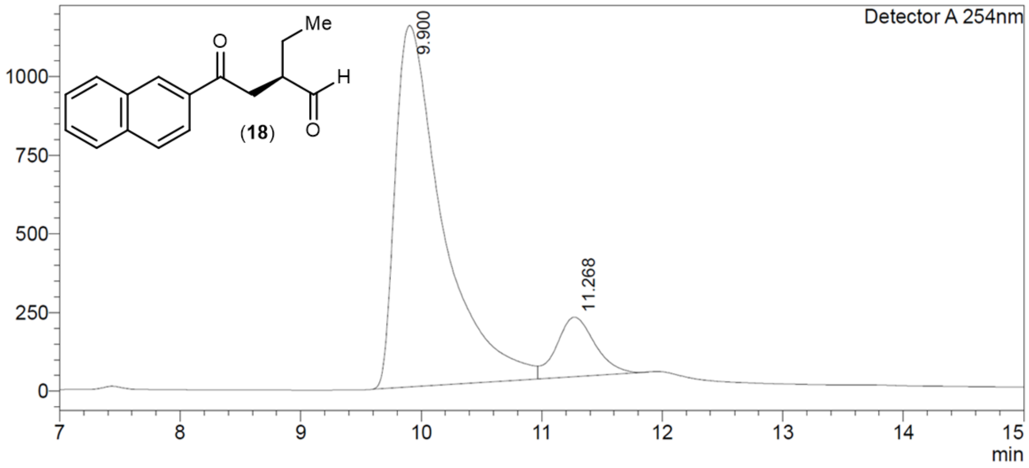

Peak#	Ret. Time	Area	Height	Area%
1	9.900	31298463	1148613	88.172
2	11.268	4198773	189019	11.828
Total		35497236	1337632	100.000

300 MHz, $CDCl_3$

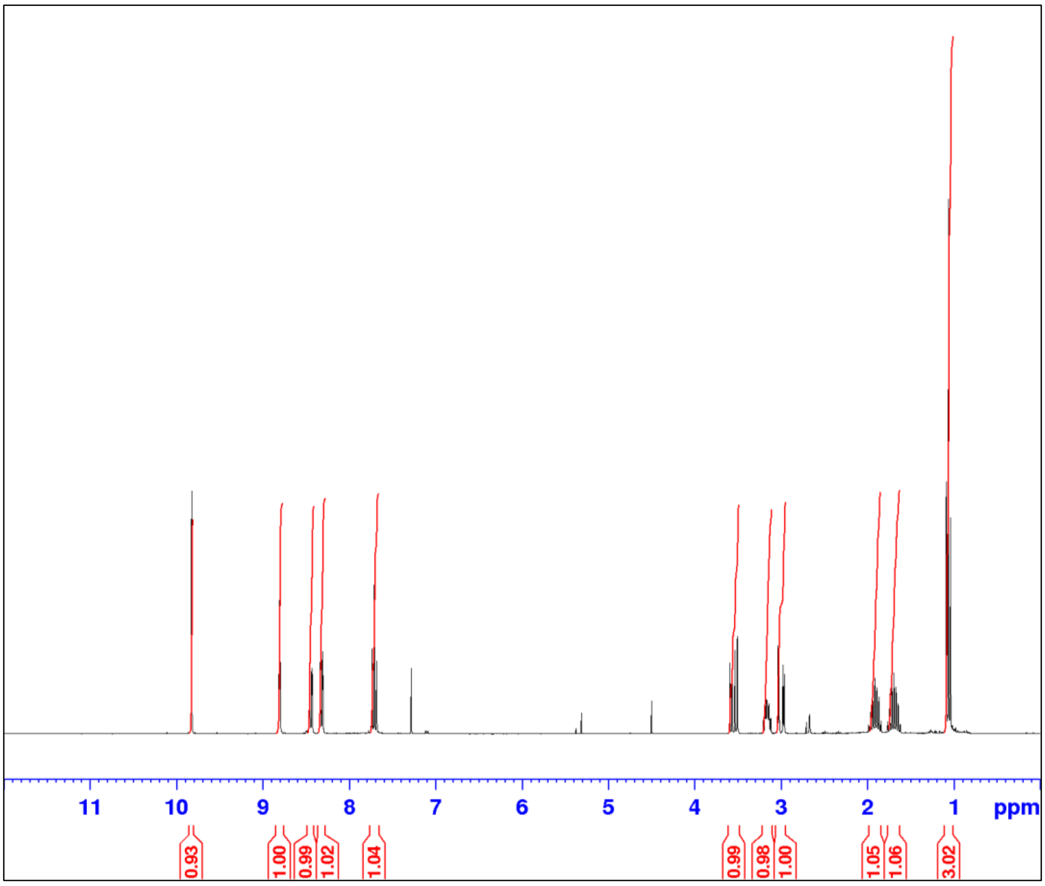

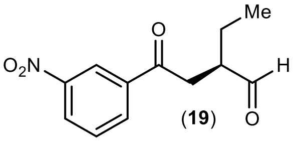

(**19**)

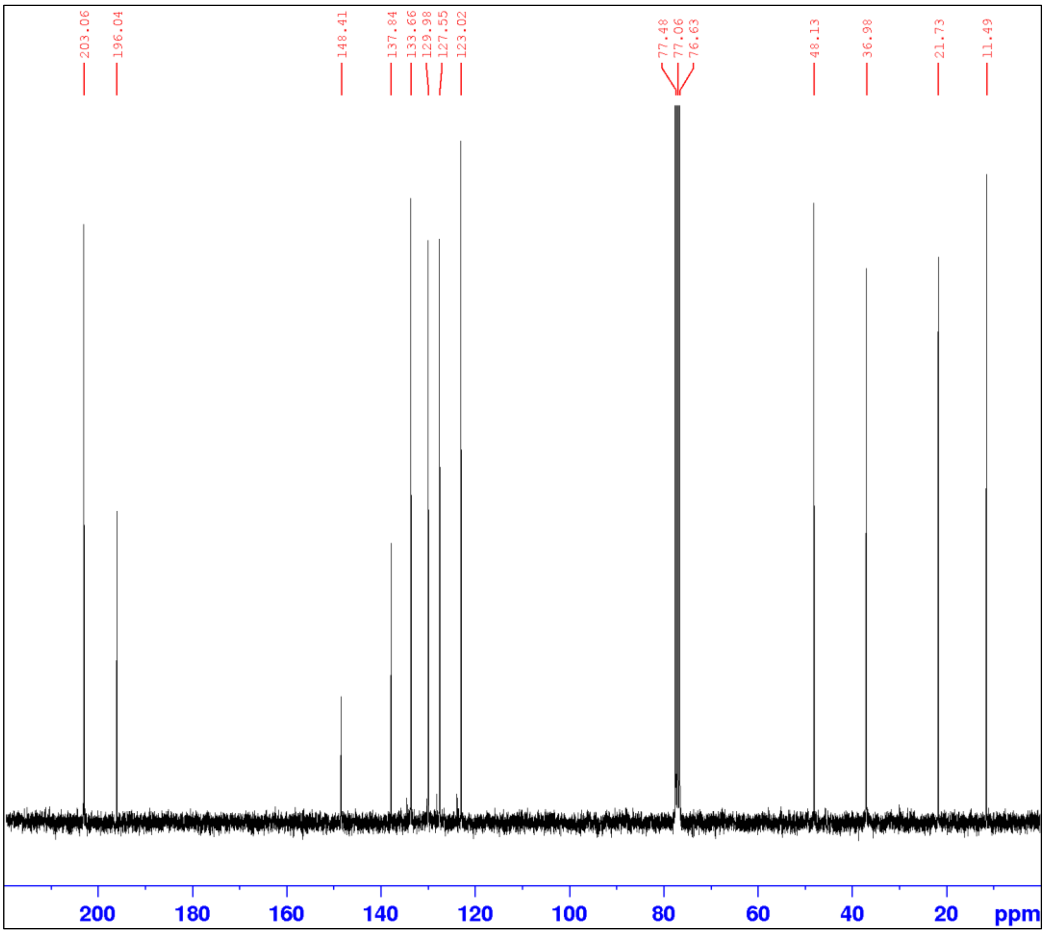

75 MHz, $CDCl_3$

HPLC chromatograms

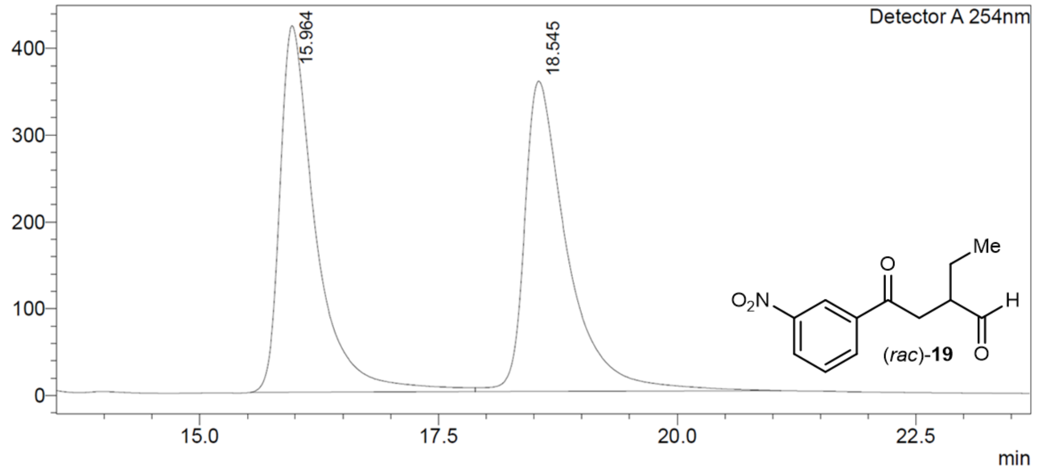

Peak#	Ret. Time	Area	Height	Area%
1	15.964	11091697	422696	49.758
2	18.545	11199496	357785	50.242
Total		22291193	780481	100.000

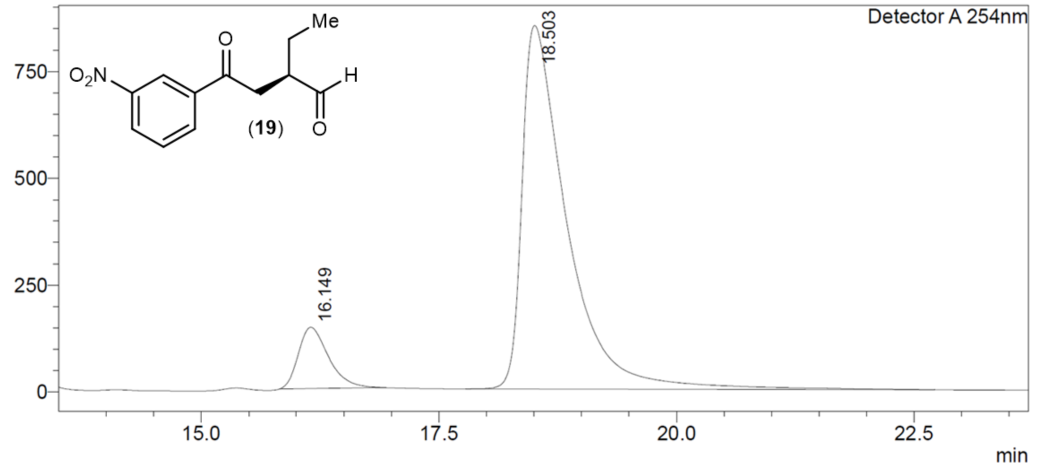

Peak#	Ret. Time	Area	Height	Area%
1	16.149	3161246	143375	10.147
2	18.503	27993239	849800	89.853
Total		31154485	993174	100.000

300 MHz, $CDCl_3$

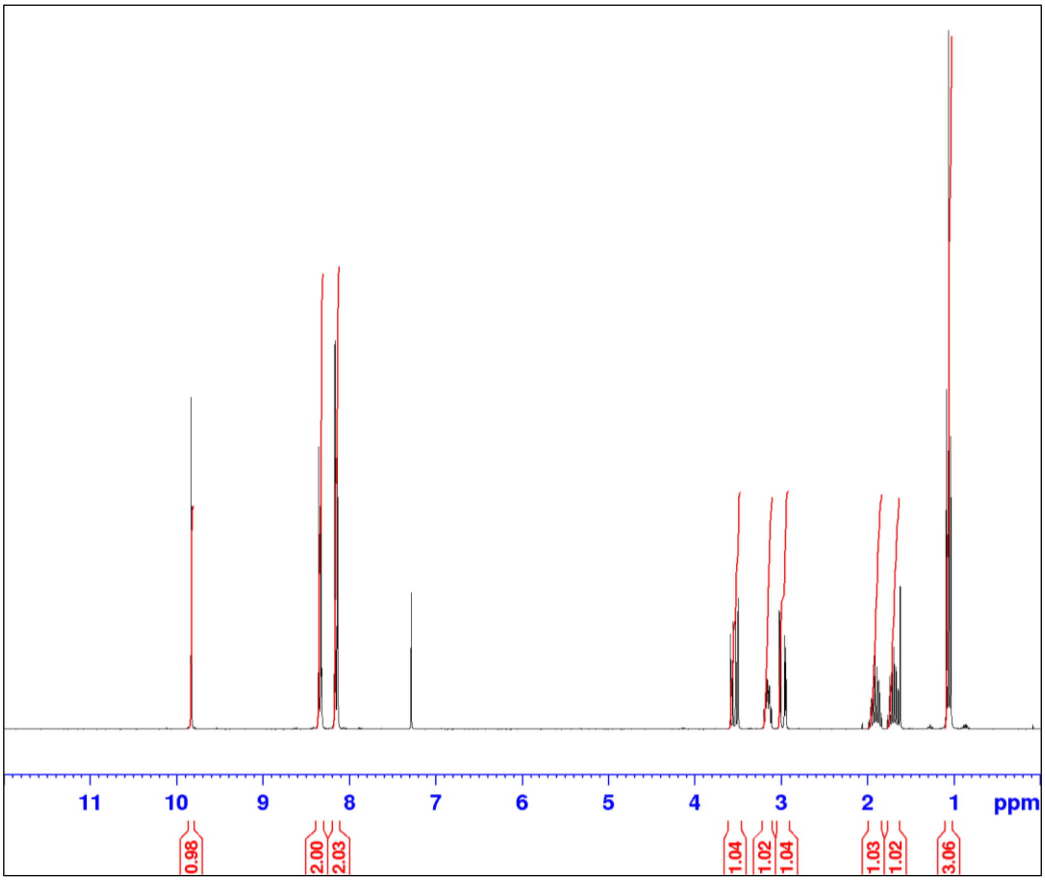

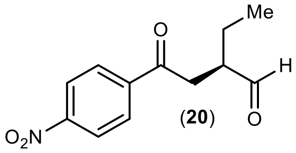

(**20**)

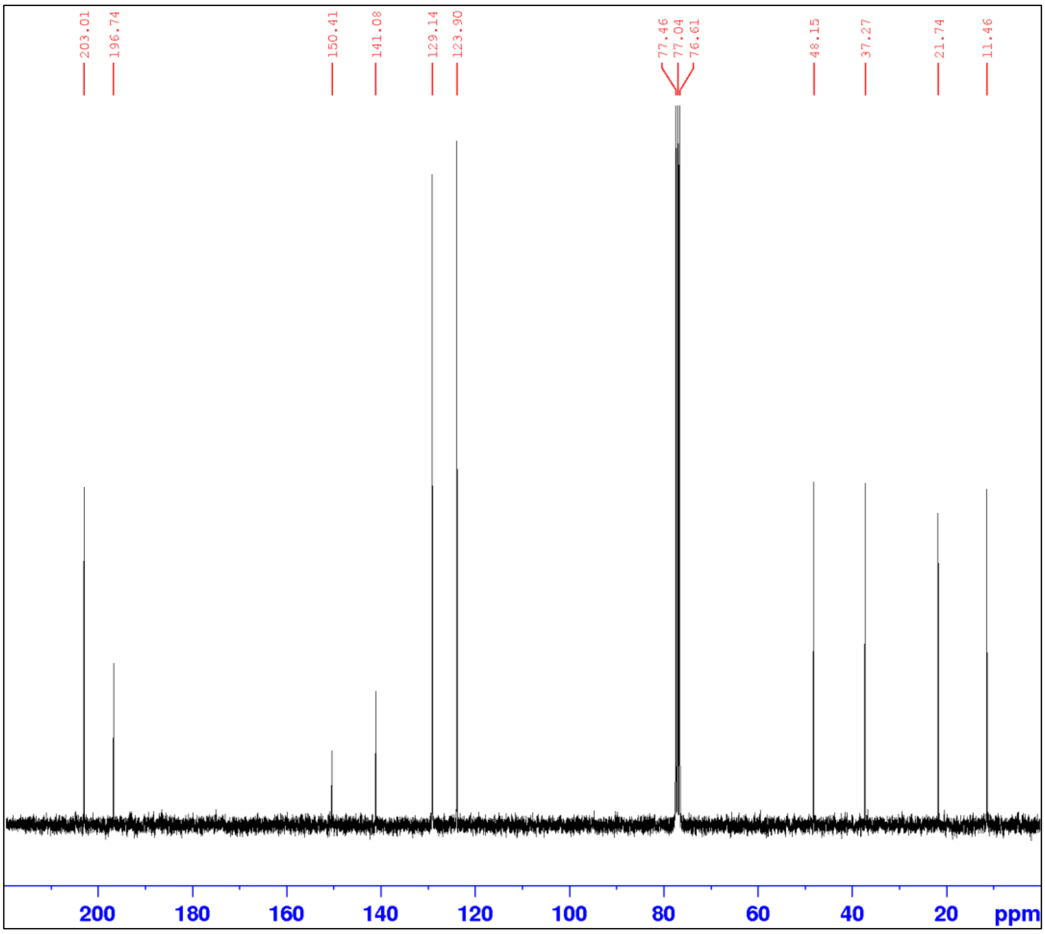

75 MHz, $CDCl_3$

HPLC chromatograms

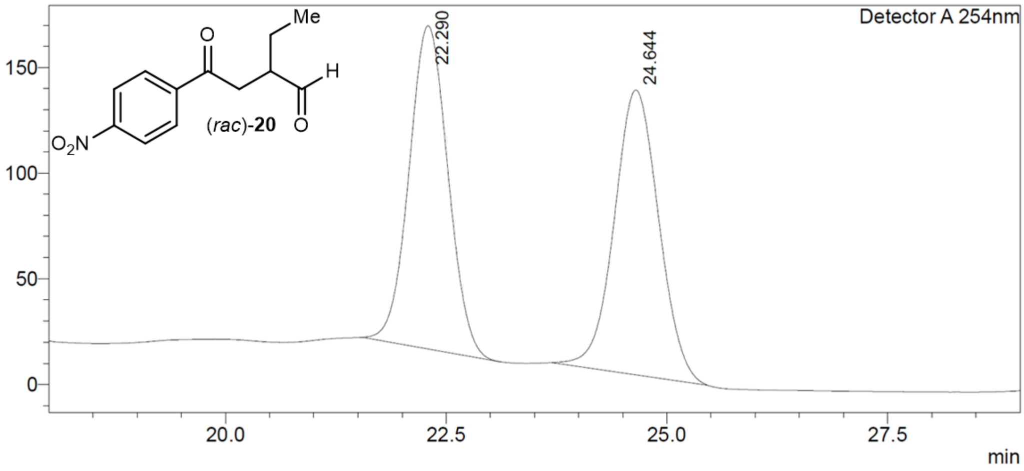

Peak#	Ret. Time	Area	Height	Area%
1	22.290	4692388	152943	49.778
2	24.644	4734281	134729	50.222
Total		9426669	287671	100.000

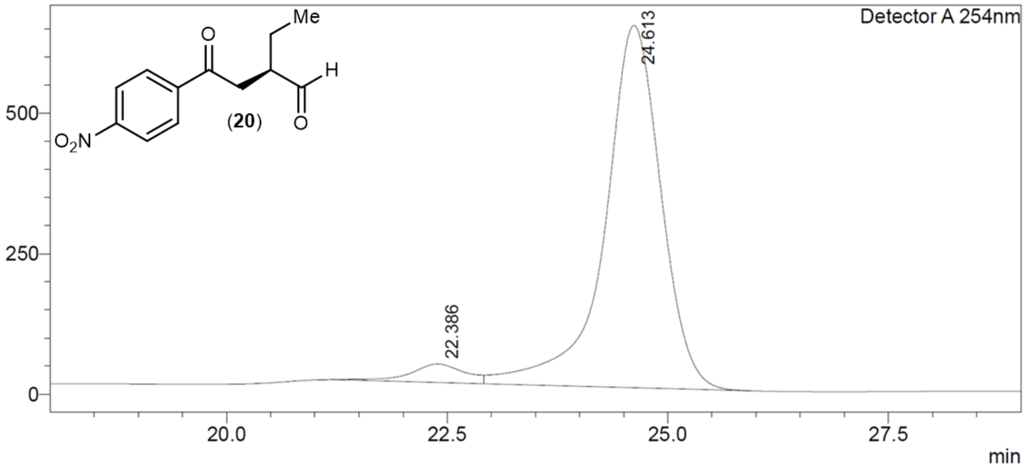

Peak#	Ret. Time	Area	Height	Area%
1	22.386	1415567	32753	4.676
2	24.613	28859674	644558	95.324
Total		30275241	677310	100.000

300 MHz, $CDCl_3$

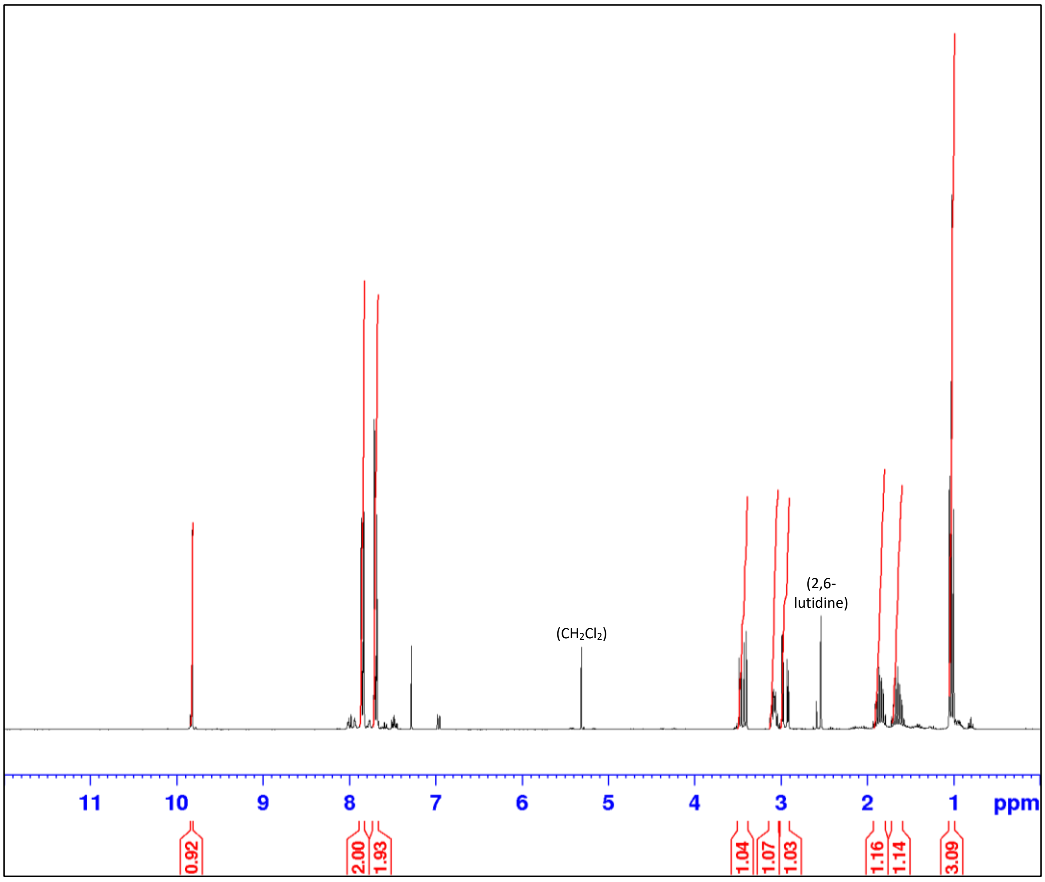

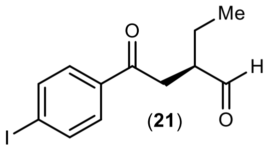

(**21**)

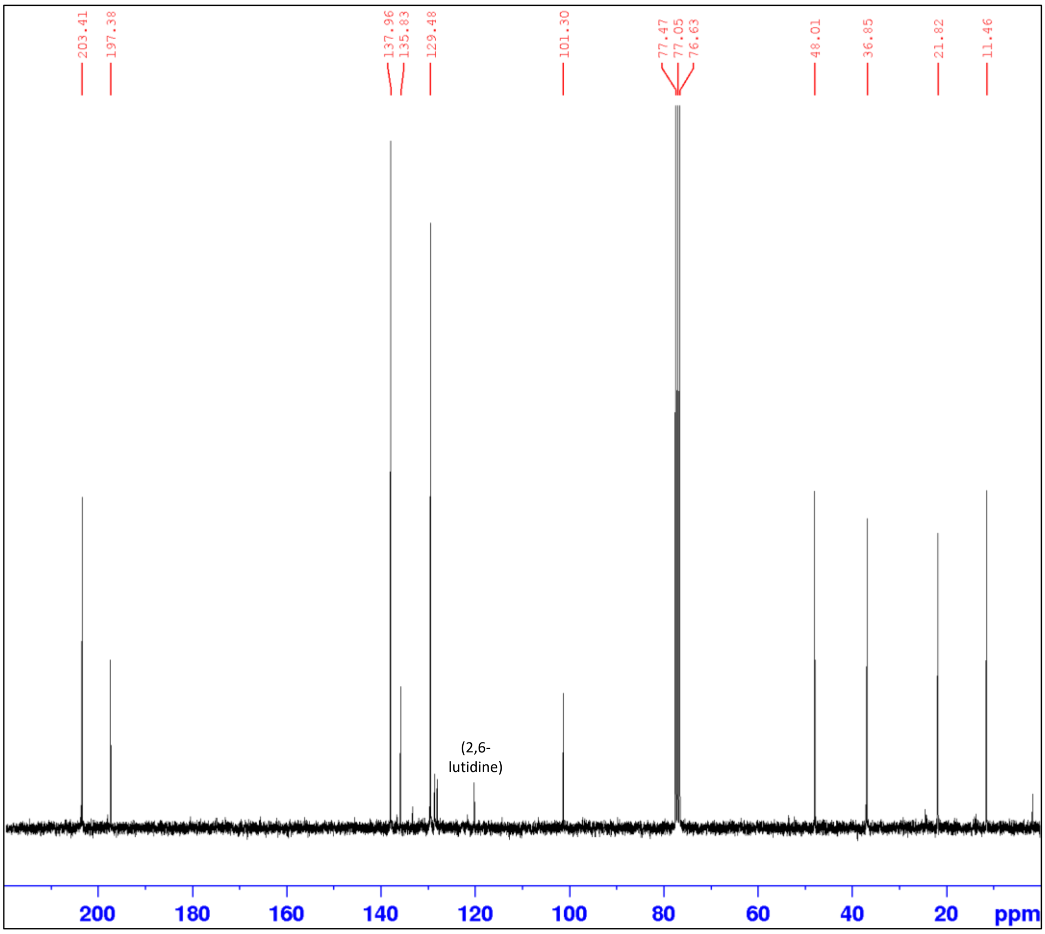

75 MHz, $CDCl_3$

HPLC chromatograms

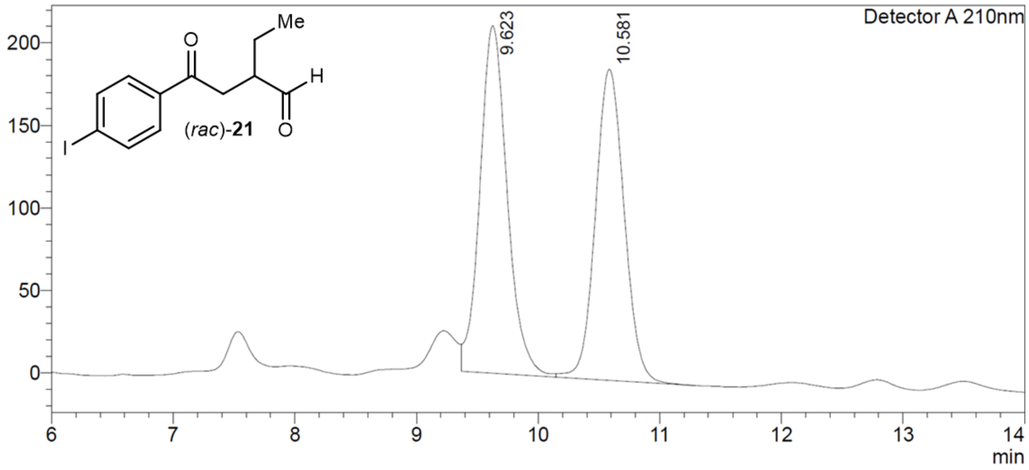

Peak#	Ret. Time	Area	Height	Area%
1	9.623	3288048	210651	51.501
2	10.581	3096348	188600	48.499
Total		6384396	399250	100.000

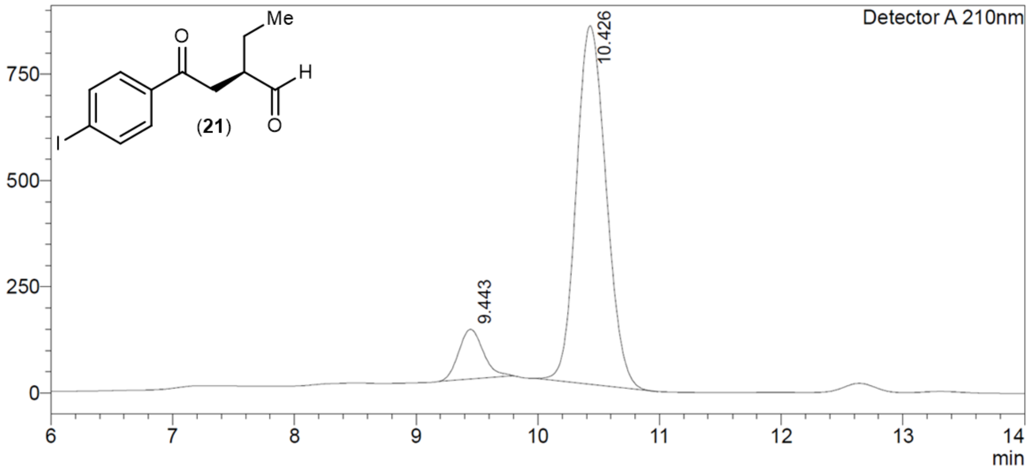

Peak#	Ret. Time	Area	Height	Area%
1	9.443	1639745	116959	10.234
2	10.426	14382097	843071	89.766
Total		16021842	960030	100.000

300 MHz, $CDCl_3$

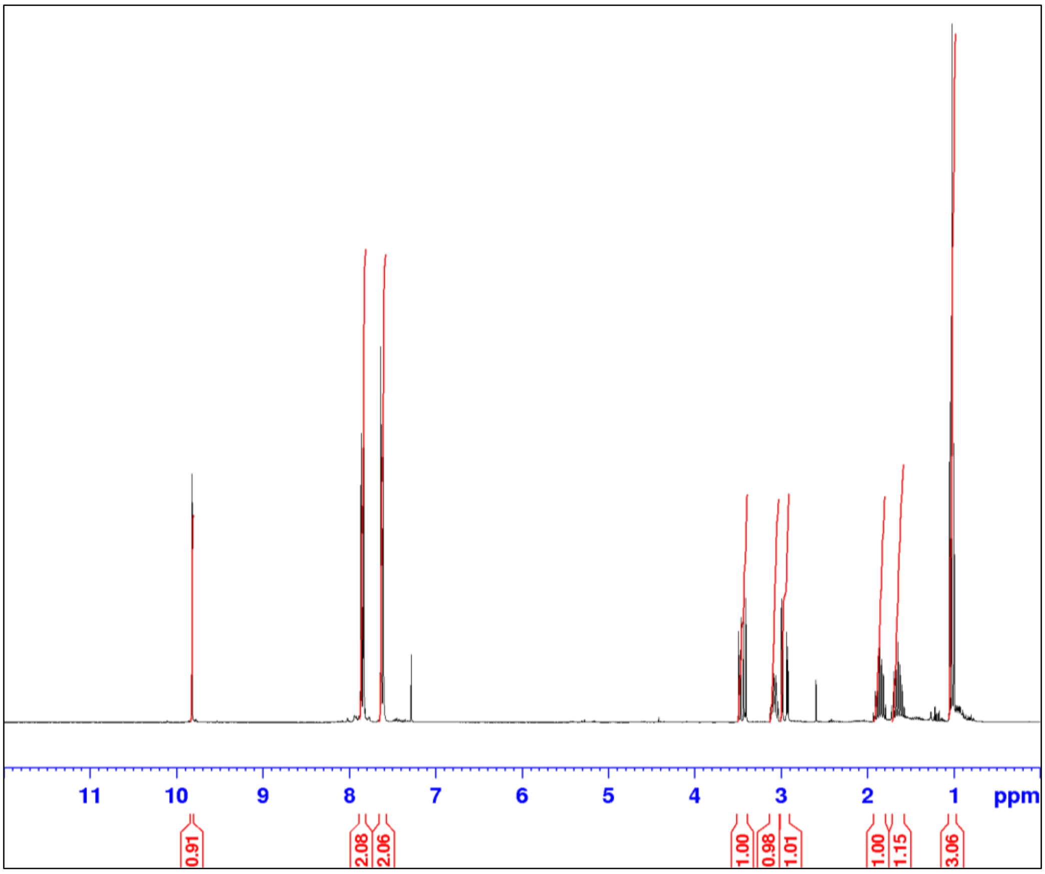

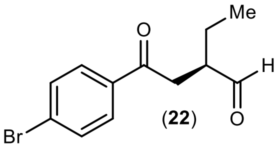

(**22**)

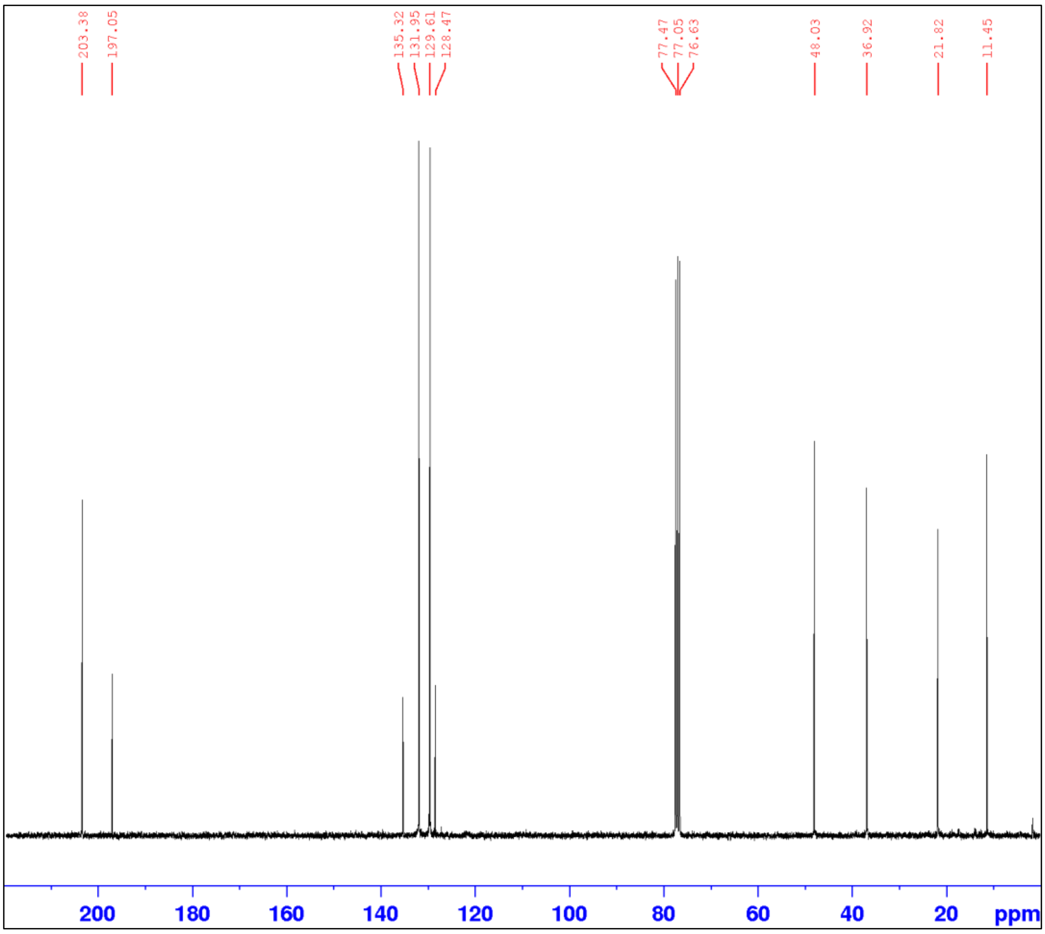

75 MHz, $CDCl_3$

HPLC chromatograms

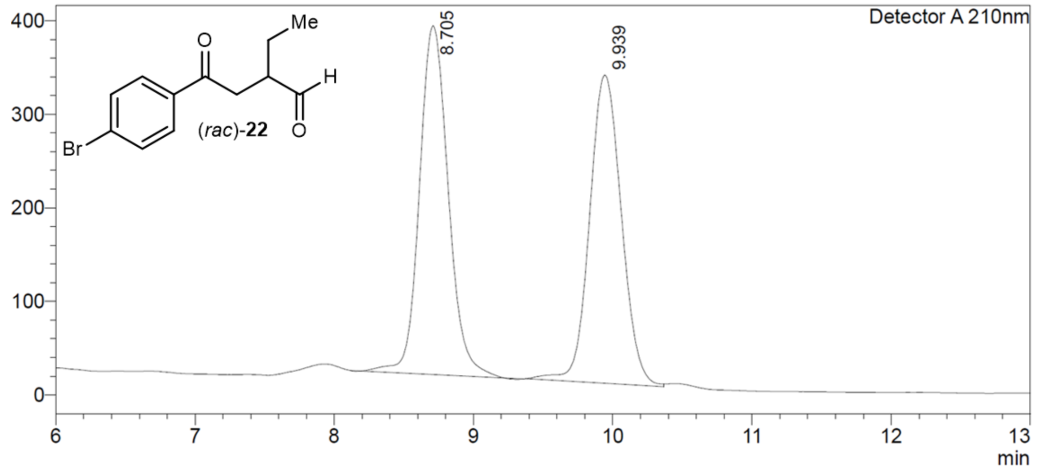

Peak#	Ret. Time	Area	Height	Area%
1	8.705	5379835	372834	50.628
2	9.939	5246353	329489	49.372
Total		10626188	702323	100.000

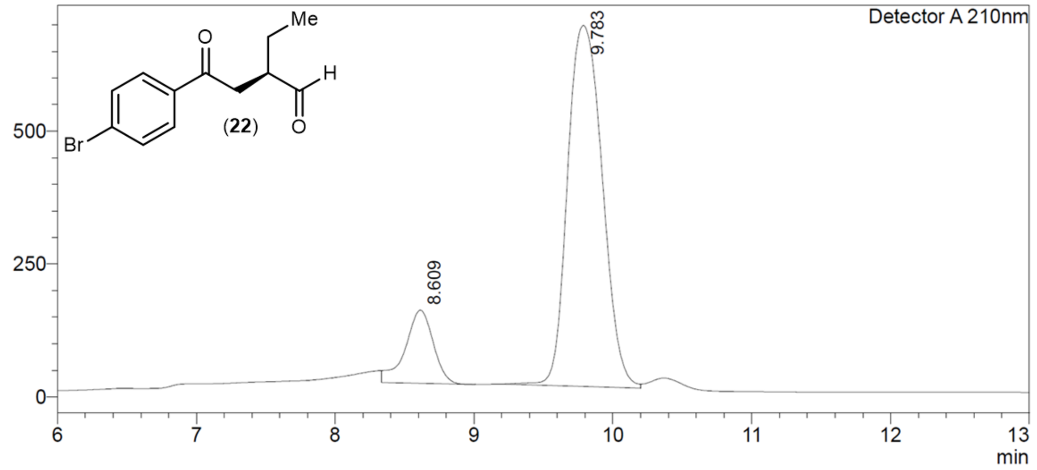

Peak#	Ret. Time	Area	Height	Area%
1	8.609	1926667	137533	13.981
2	9.783	11853641	679197	86.019
Total		13780308	816730	100.000

300 MHz, CDCl$_3$

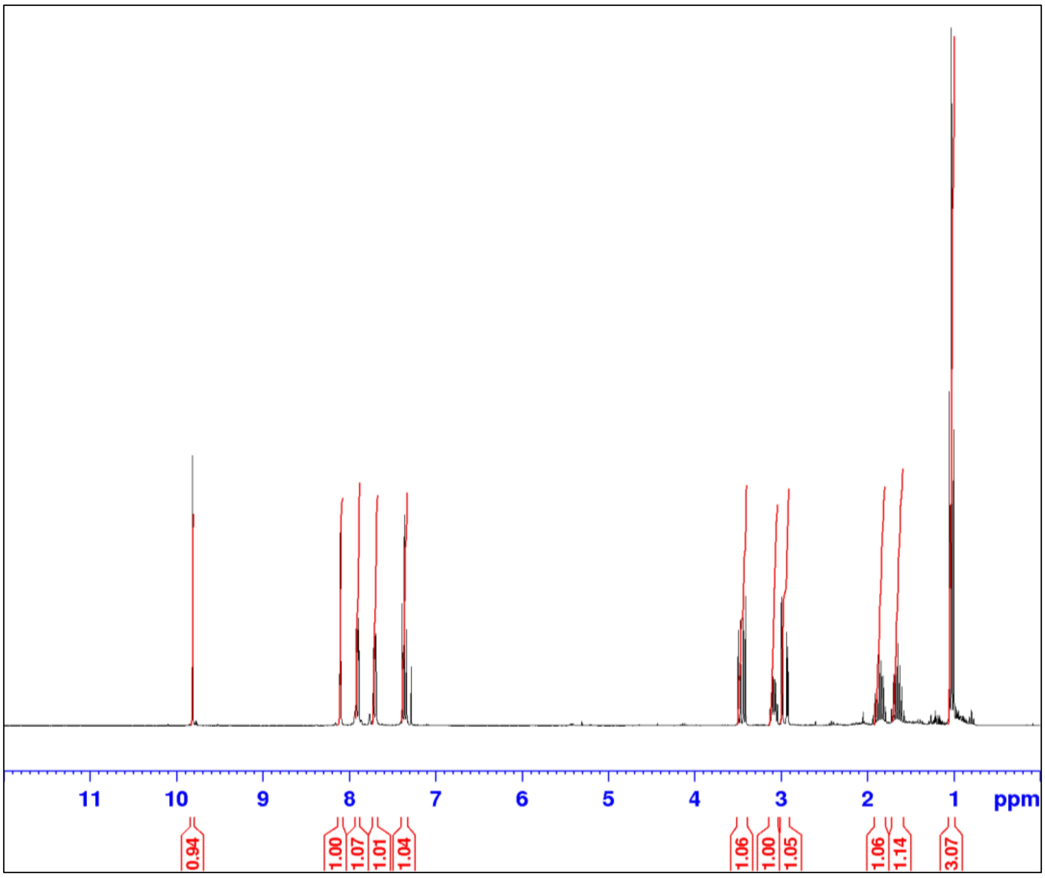

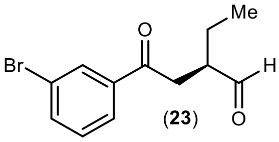

(**23**)

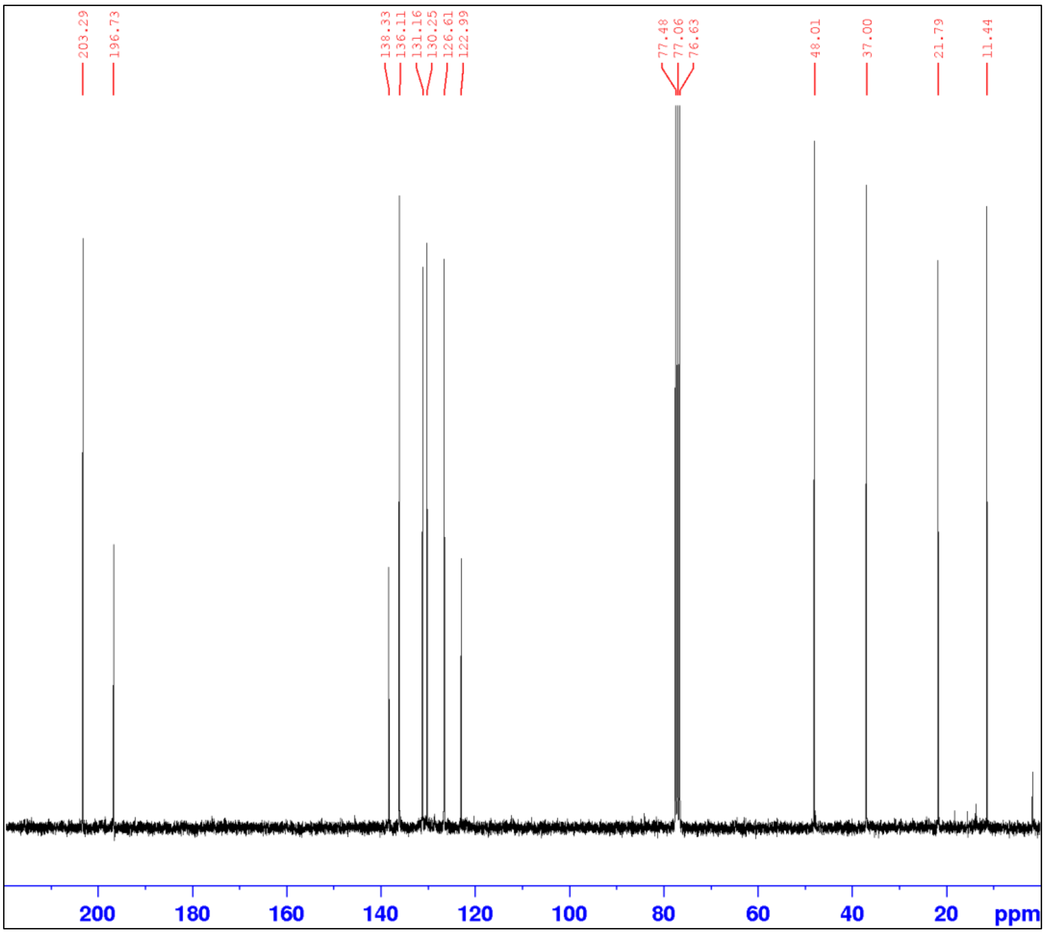

75 MHz, CDCl$_3$

HPLC chromatograms

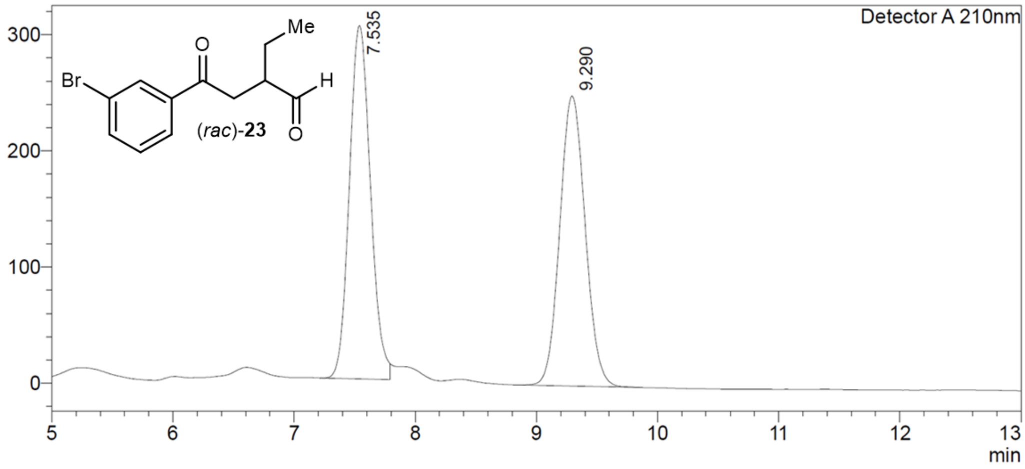

Peak#	Ret. Time	Area	Height	Area%
1	7.535	3680510	304078	50.264
2	9.290	3641874	249730	49.736
Total		7322384	553808	100.000

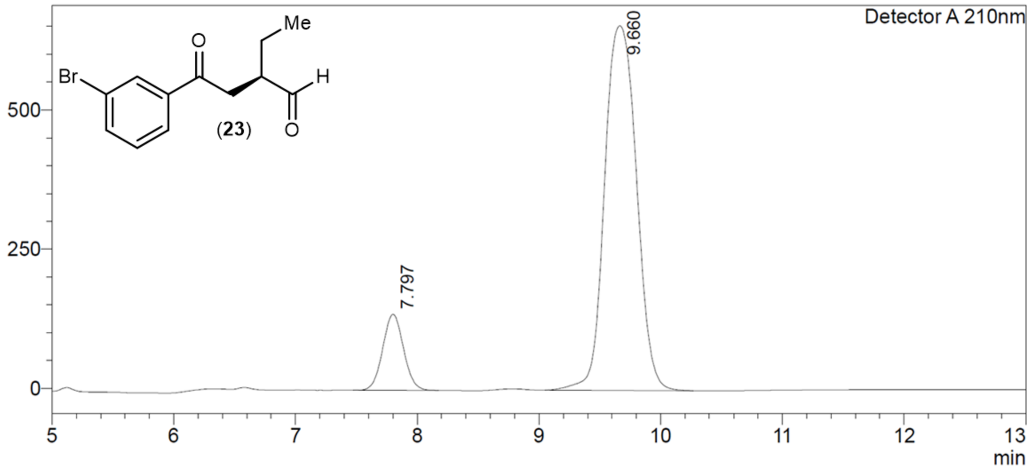

Peak#	Ret. Time	Area	Height	Area%
1	7.797	1488671	131839	11.082
2	9.660	11944061	654656	88.918
Total		13432733	786494	100.000

300 MHz, $CDCl_3$

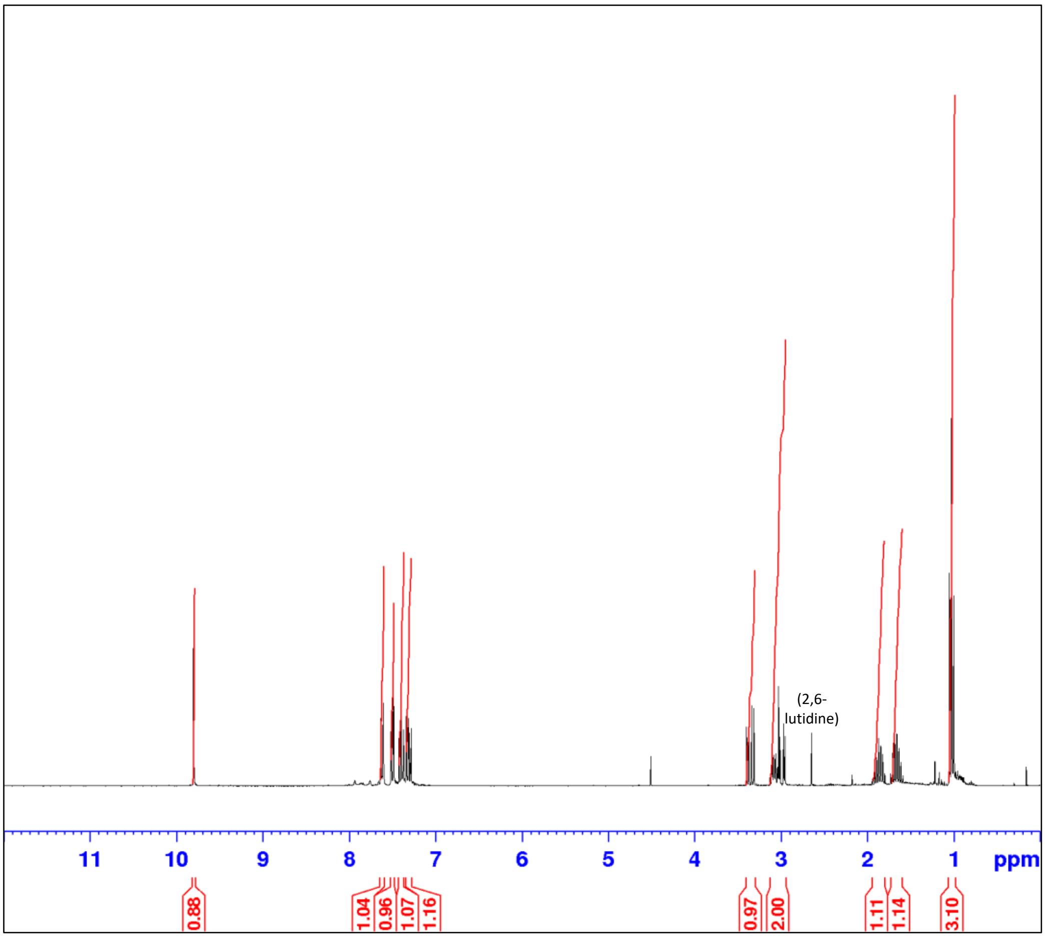

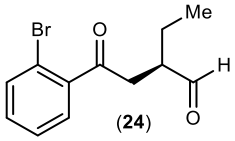

(**24**)

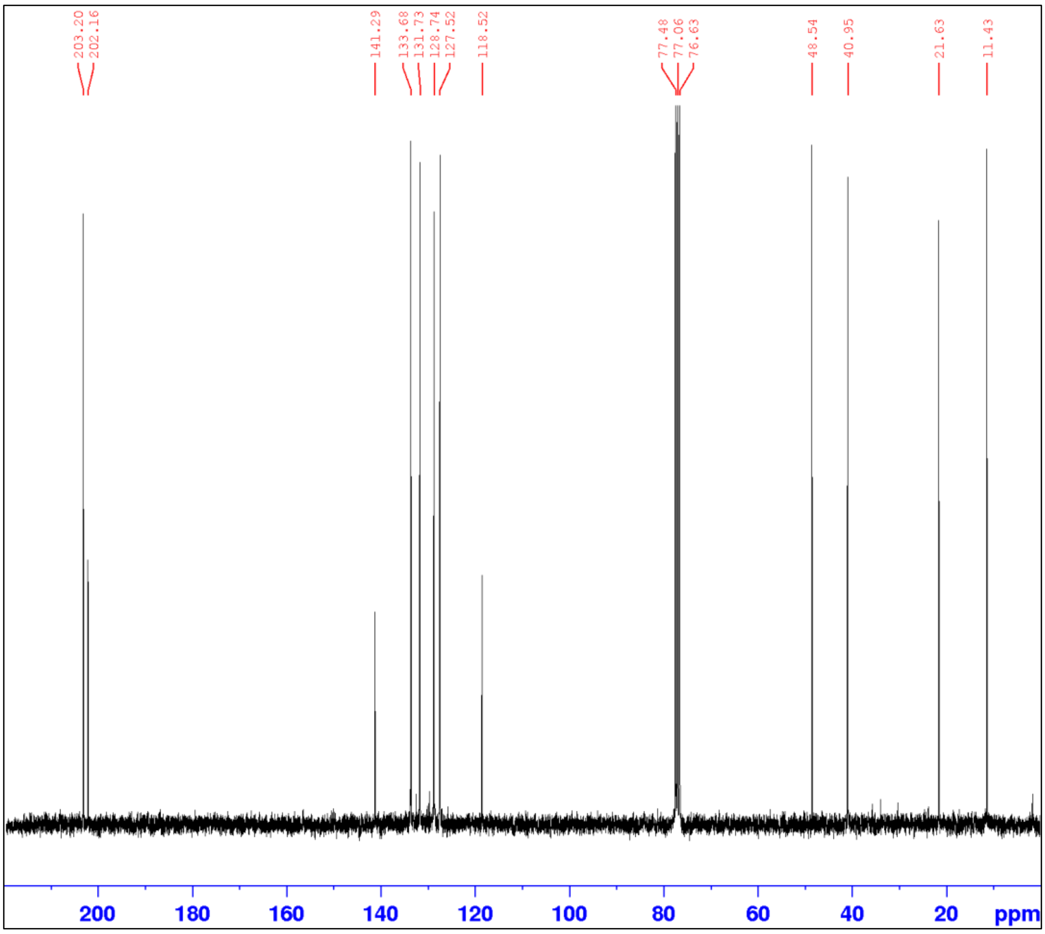

75 MHz, $CDCl_3$

HPLC chromatograms

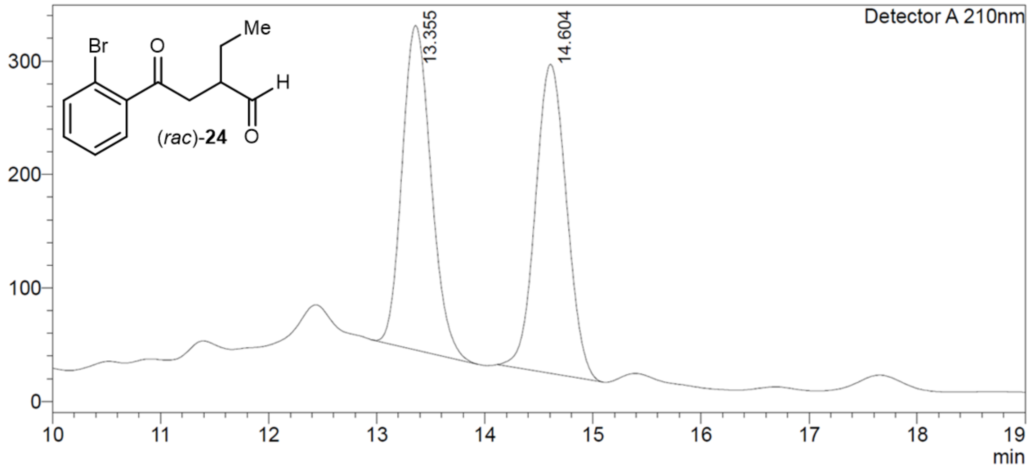

Peak#	Ret. Time	Area	Height	Area%
1	13.355	5335303	285955	49.432
2	14.604	5457827	272413	50.568
Total		10793129	558368	100.000

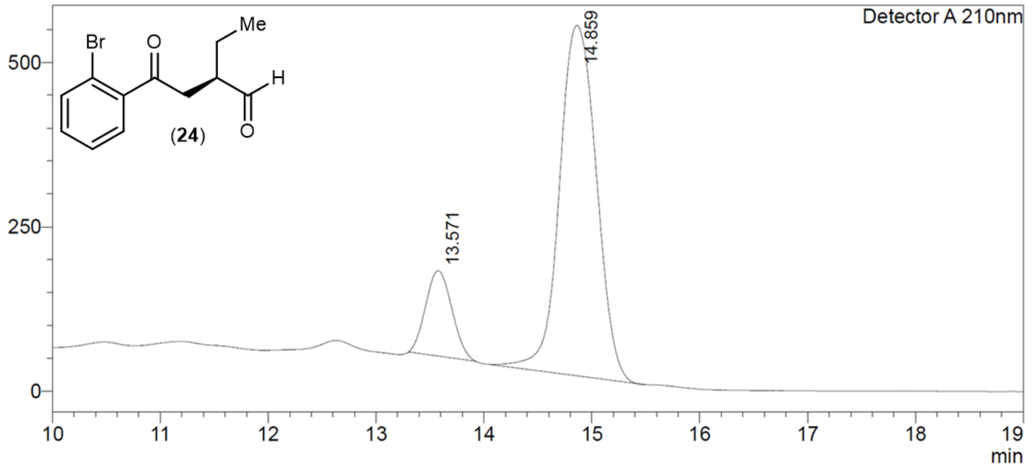

Peak#	Ret. Time	Area	Height	Area%
1	13.571	2200714	130036	14.757
2	14.859	12712543	532370	85.243
Total		14913257	662406	100.000

300 MHz, $CDCl_3$

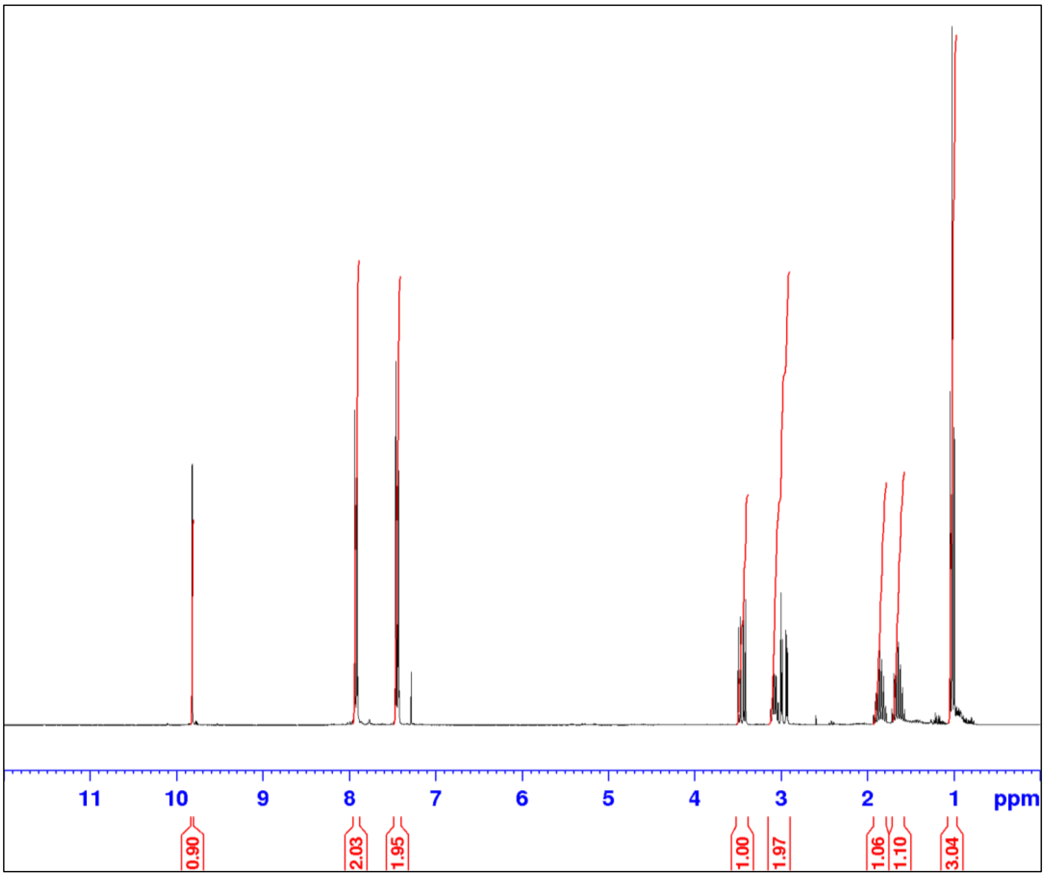

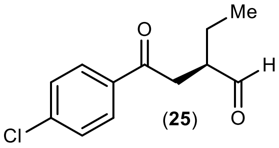

(**25**)

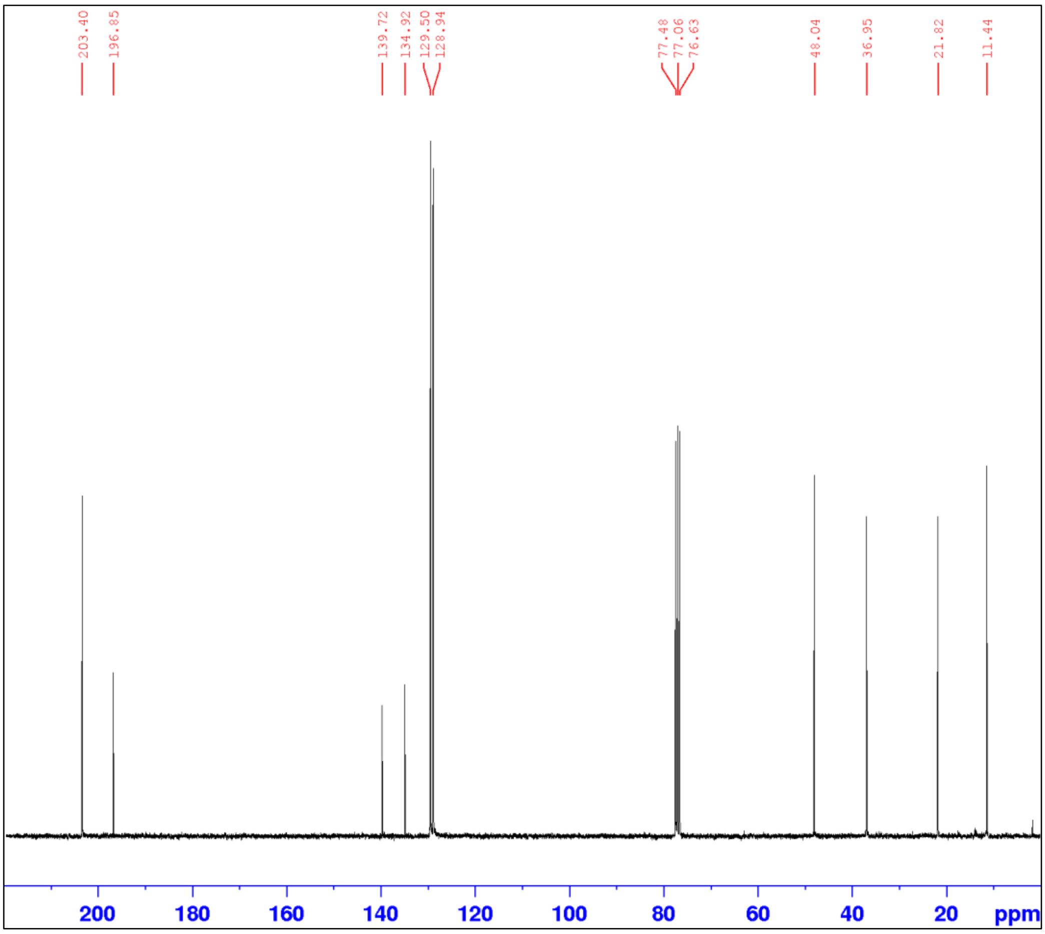

75 MHz, $CDCl_3$

HPLC chromatograms

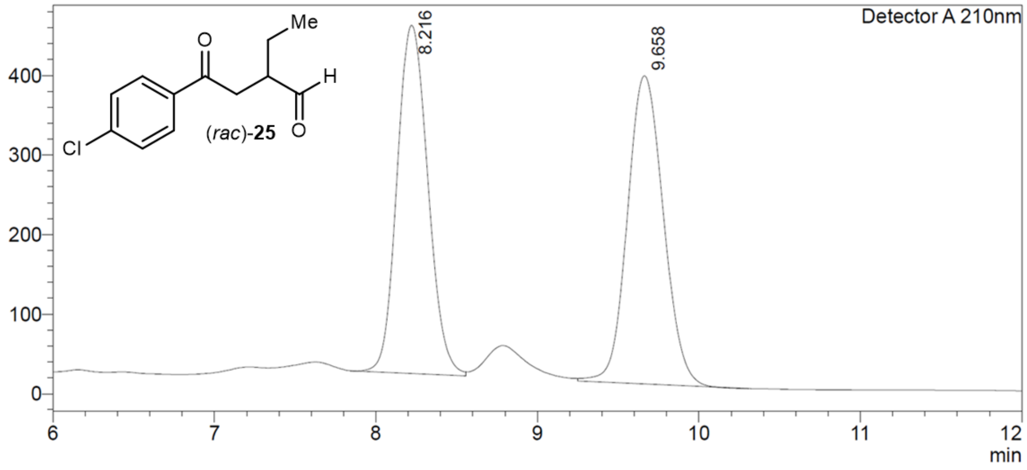

Peak#	Ret. Time	Area	Height	Area%
1	8.216	5825729	437582	49.213
2	9.658	6012017	387299	50.787
Total		11837745	824881	100.000

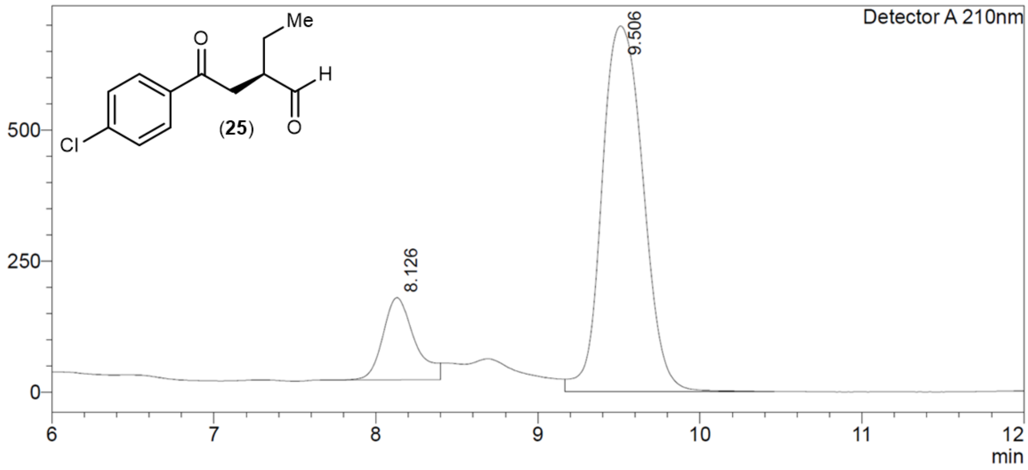

Peak#	Ret. Time	Area	Height	Area%
1	8.126	2010265	150412	14.027
2	9.506	12320822	697384	85.973
Total		14331088	847796	100.000

300 MHz, $CDCl_3$

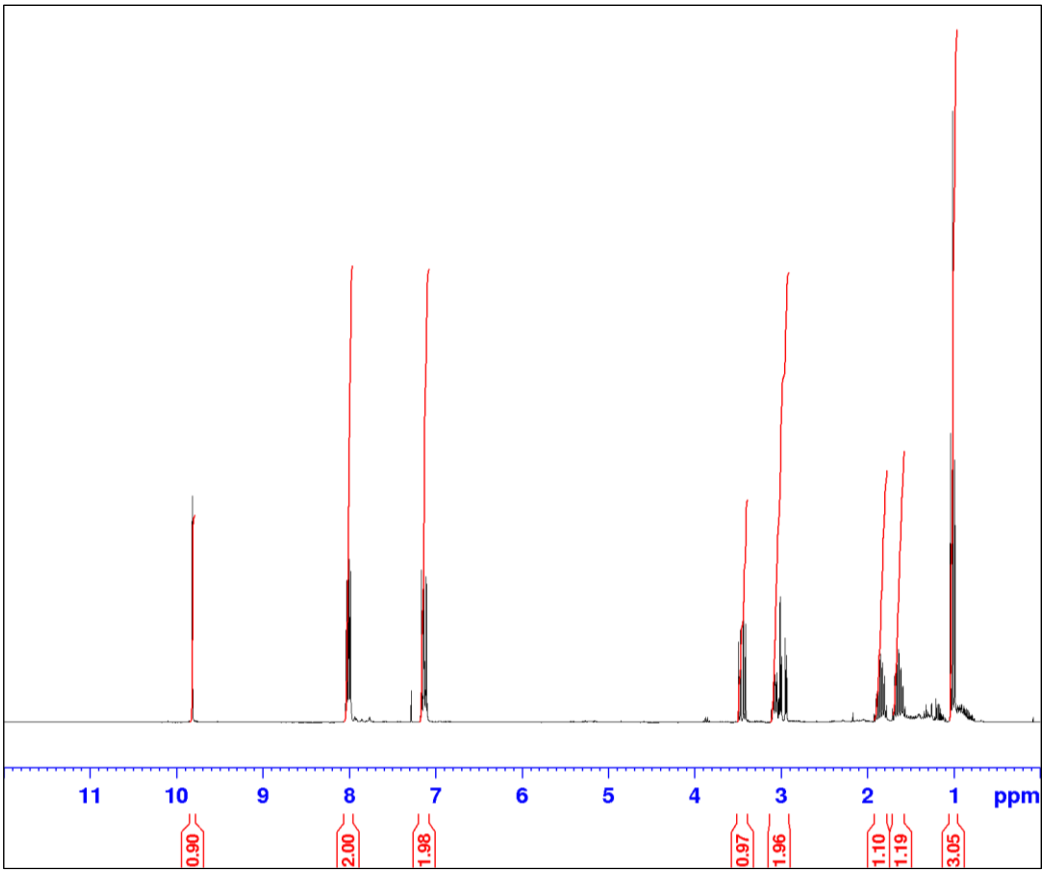

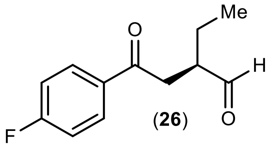

(**26**)

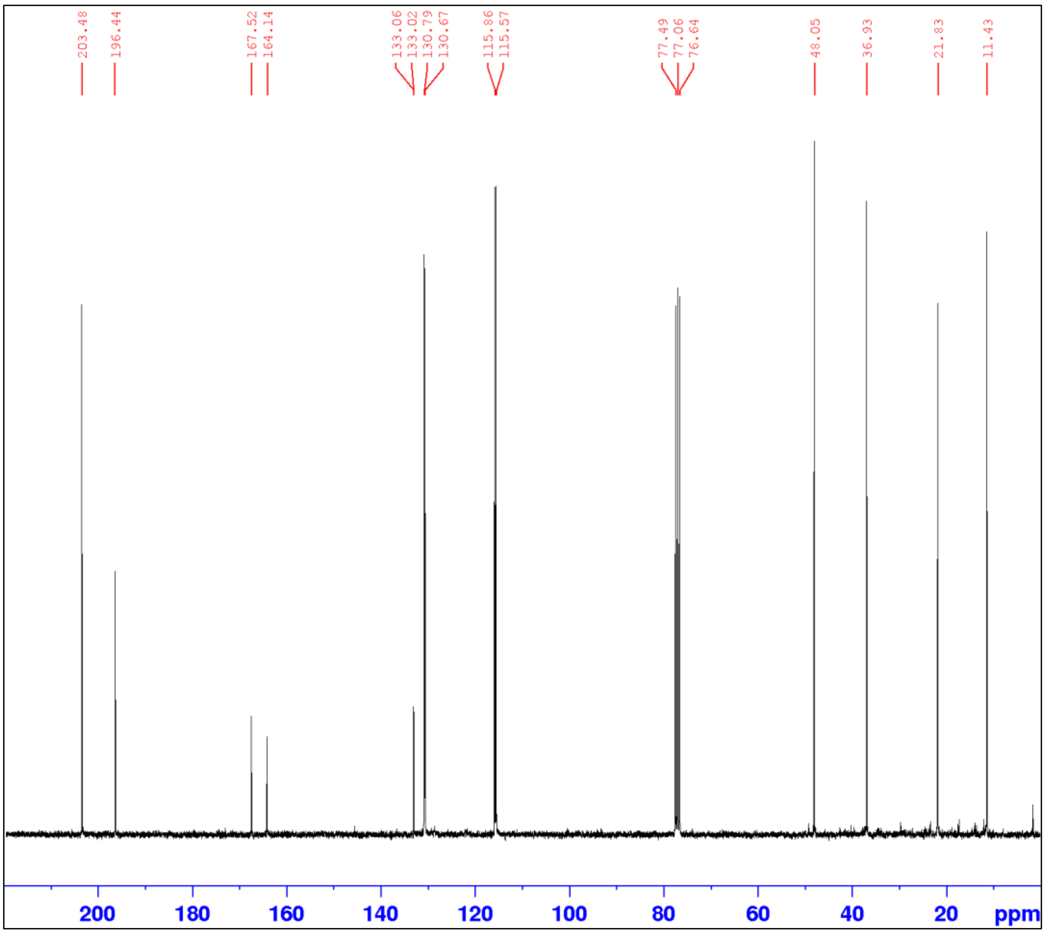

75 MHz, $CDCl_3$

HPLC chromatograms

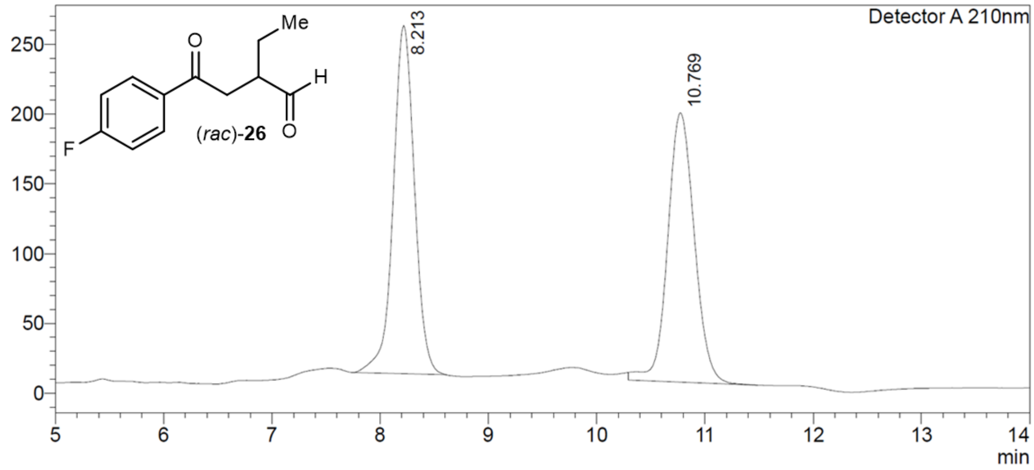

Peak#	Ret. Time	Area	Height	Area%
1	8.213	3414492	249610	50.707
2	10.769	3319273	192984	49.293
Total		6733765	442594	100.000

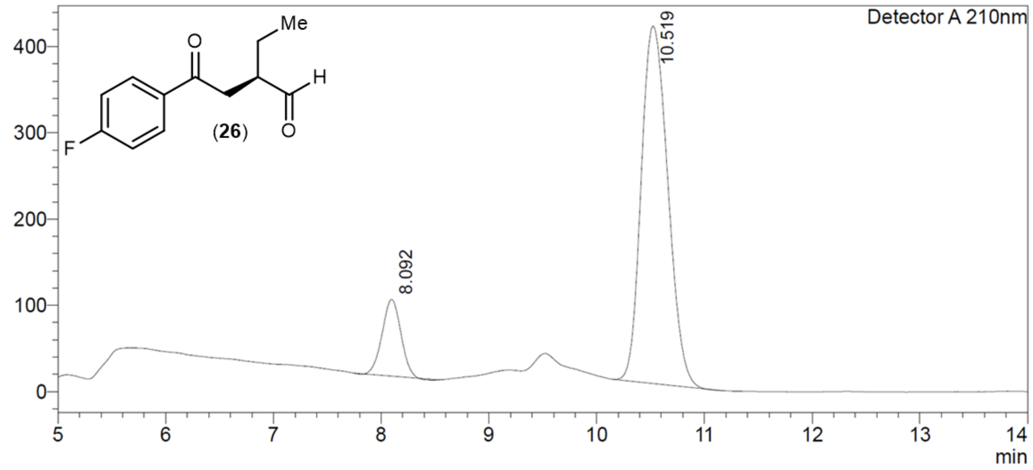

Peak#	Ret. Time	Area	Height	Area%
1	8.092	1070232	88877	13.051
2	10.519	7129997	414450	86.949
Total		8200229	503328	100.000

300 MHz, $CDCl_3$

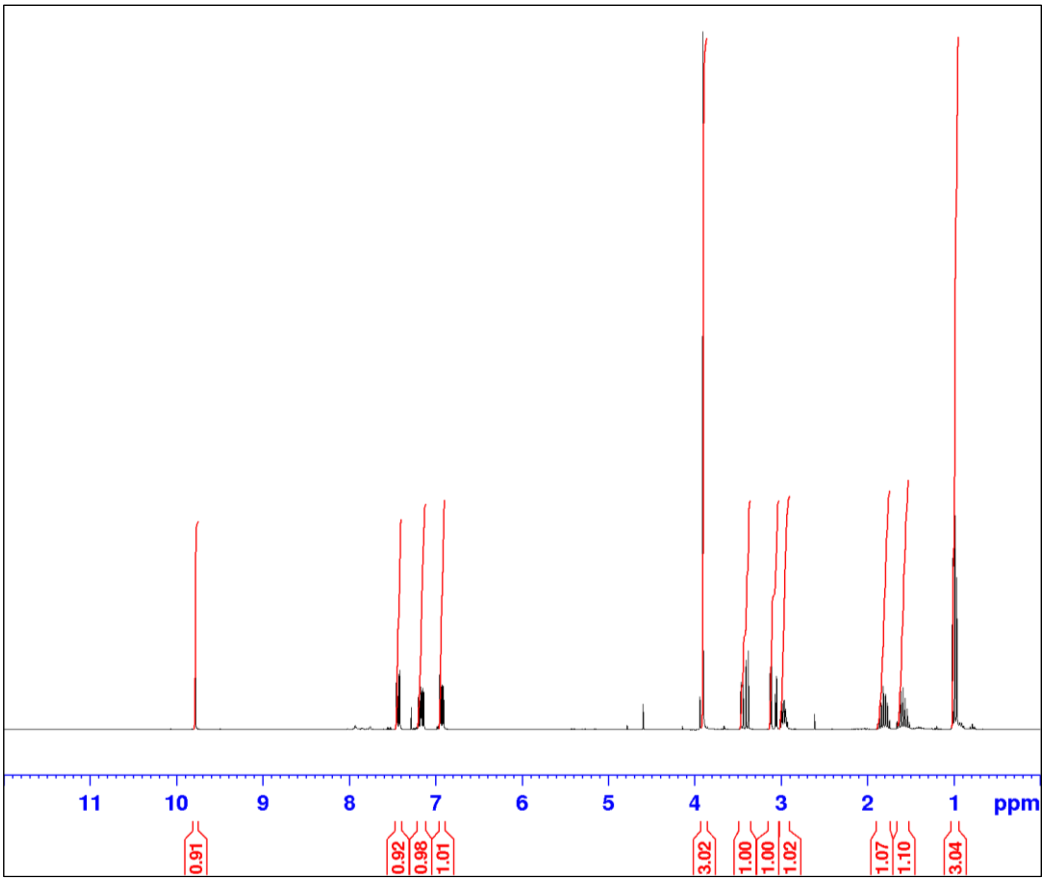

(**27**)

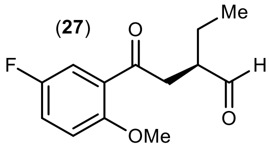

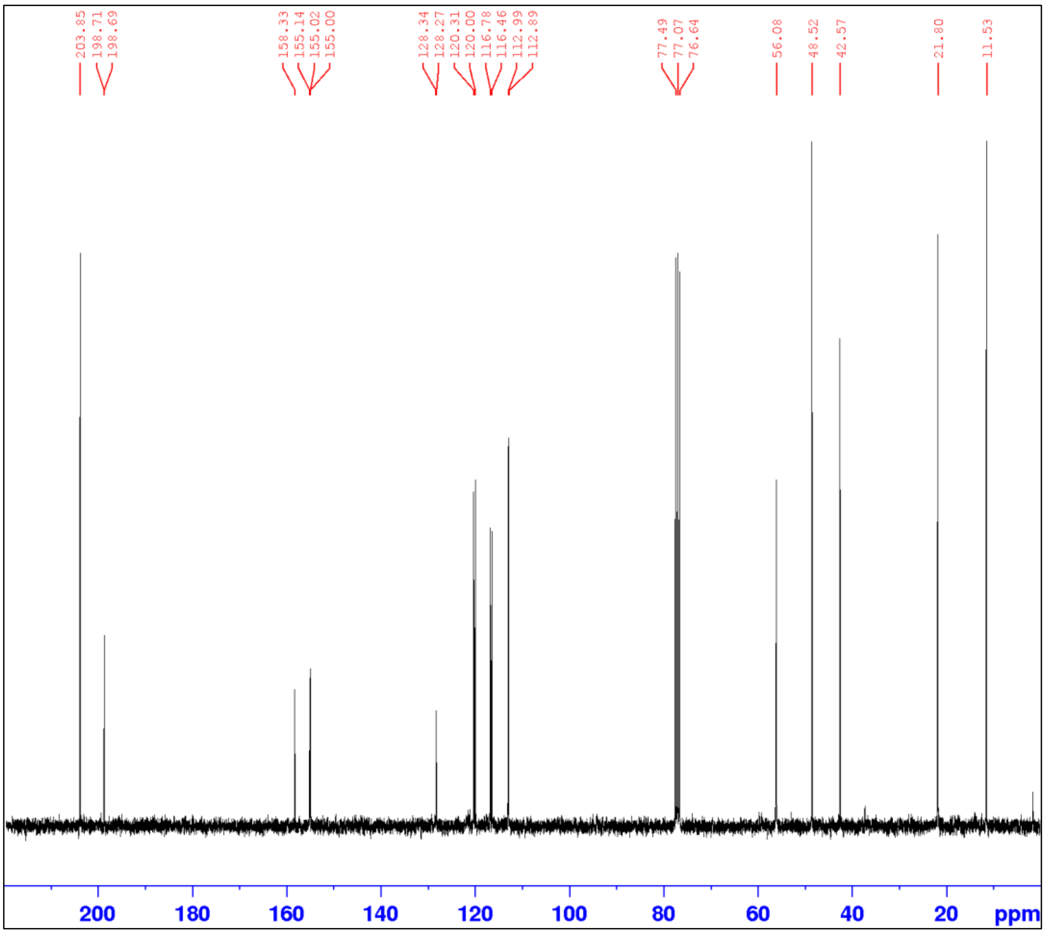

75 MHz, $CDCl_3$

HPLC chromatograms

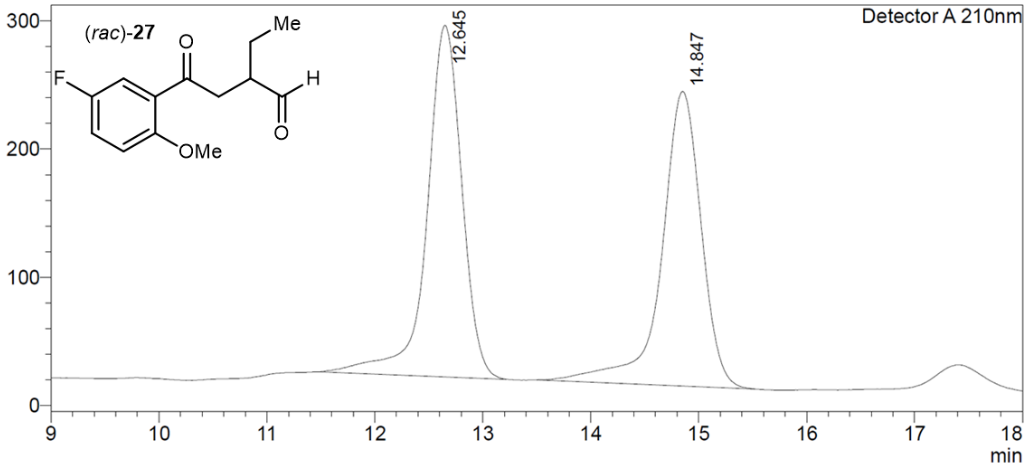

Peak#	Ret. Time	Area	Height	Area%
1	12.645	6187112	273997	51.275
2	14.847	5879341	229520	48.725
Total		12066453	503517	100.000

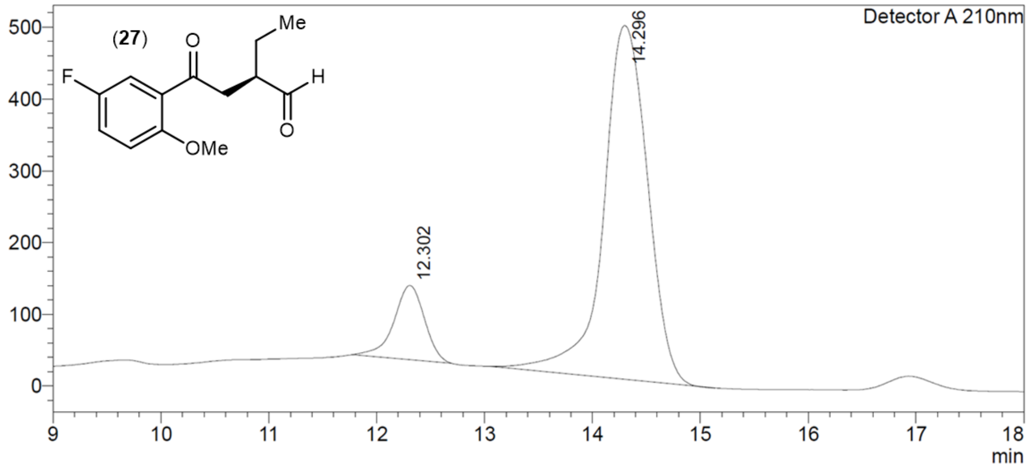

Peak#	Ret. Time	Area	Height	Area%
1	12.302	2042429	103244	12.657
2	14.296	14094717	492558	87.343
Total		16137146	595802	100.000

300 MHz, CDCl$_3$

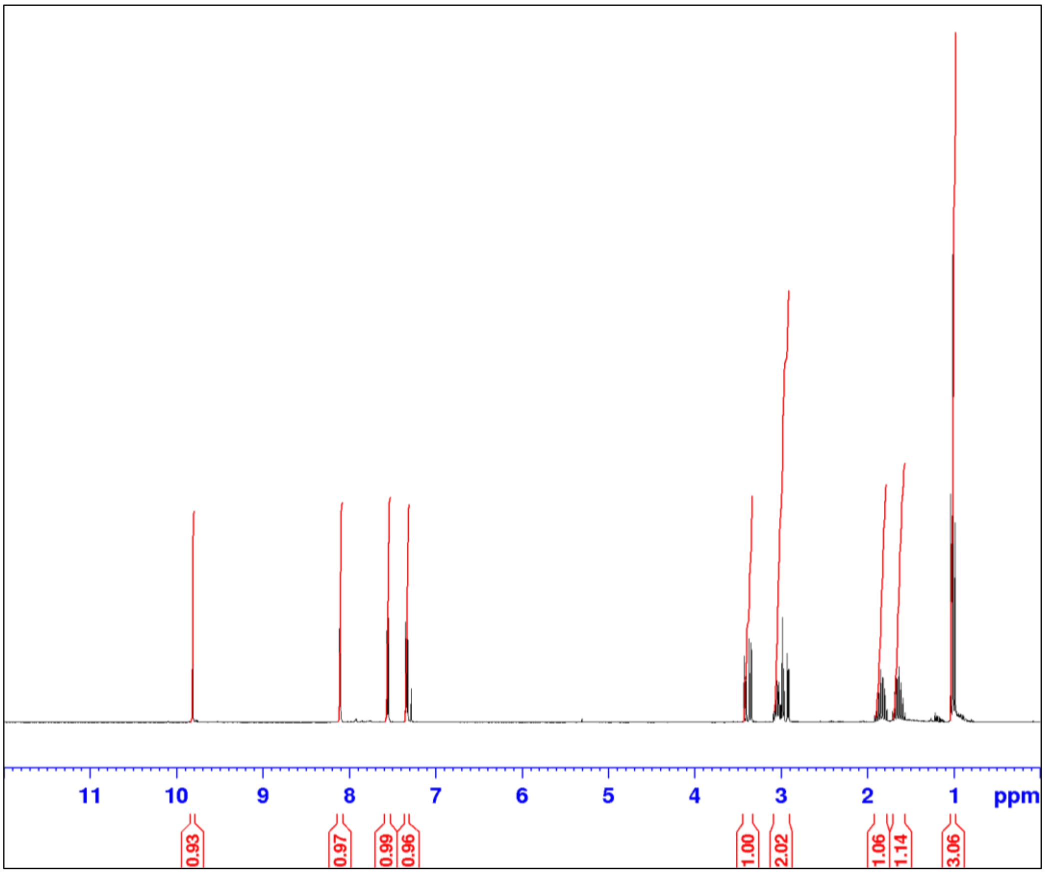

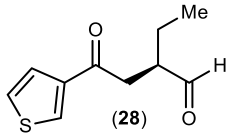

(**28**)

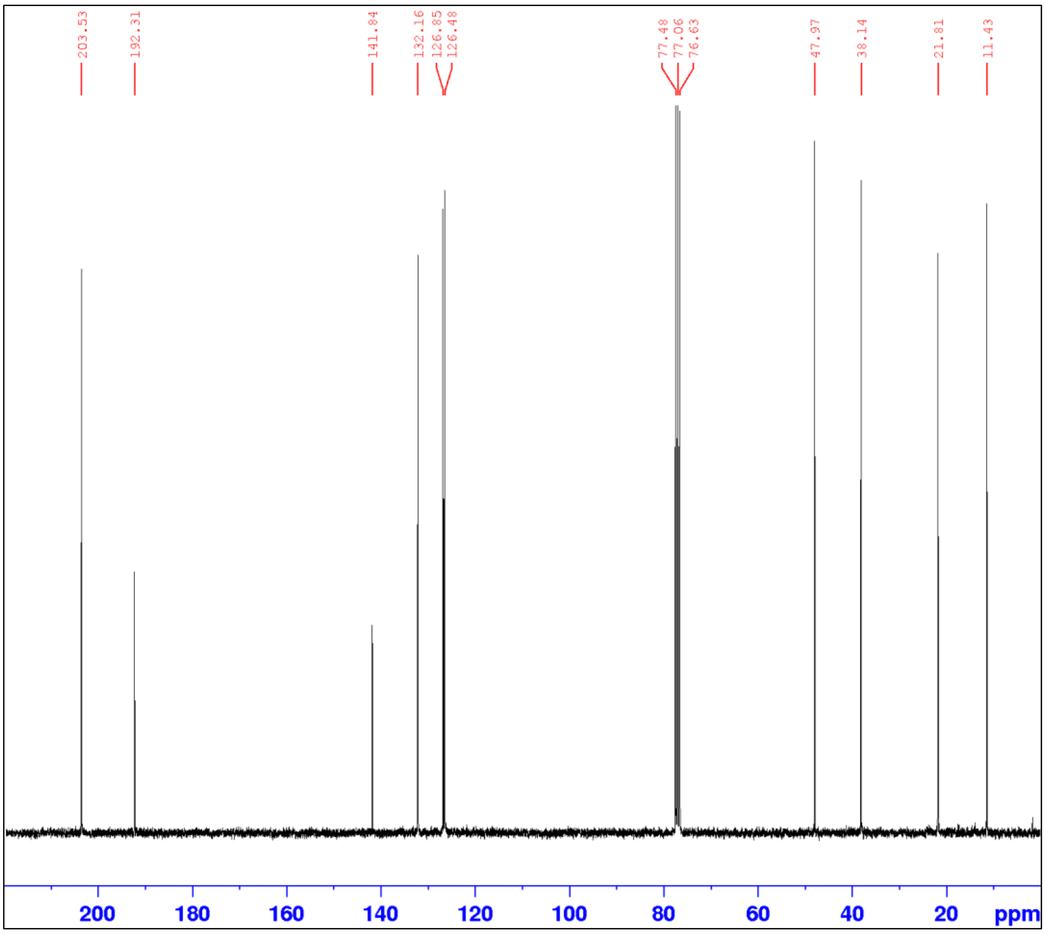

75 MHz, CDCl$_3$

HPLC chromatograms

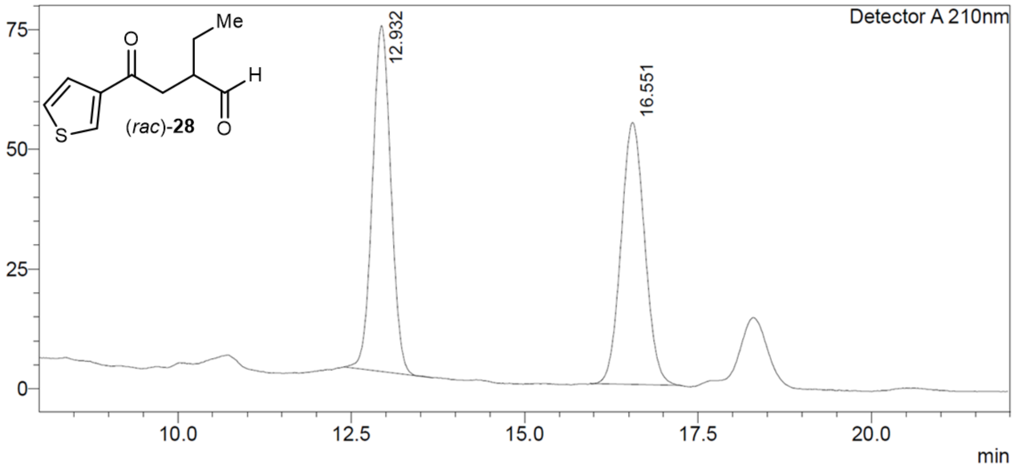

Peak#	Ret. Time	Area	Height	Area%
1	12.932	1335749	72204	50.726
2	16.551	1297504	54662	49.274
Total		2633253	126866	100.000

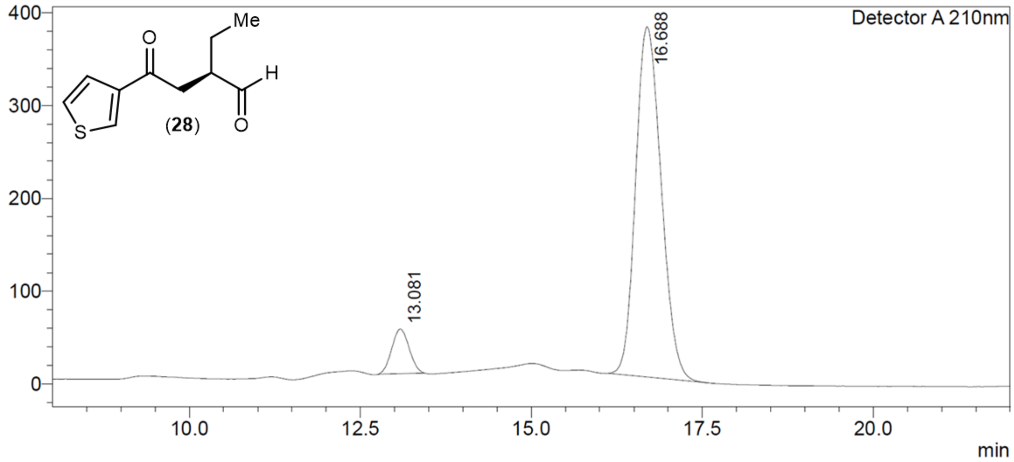

Peak#	Ret. Time	Area	Height	Area%
1	13.081	762159	45608	7.217
2	16.688	9798093	377240	92.783
Total		10560252	422847	100.000

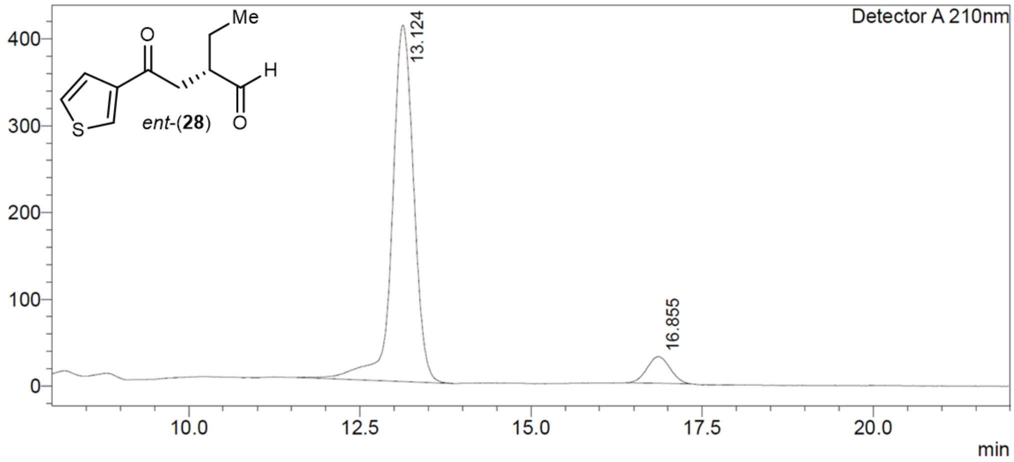

Peak#	Ret. Time	Area	Height	Area%
1	13.124	9031627	410619	92.605
2	16.855	721262	30729	7.395
Total		9752890	441347	100.000

300 MHz, $CDCl_3$

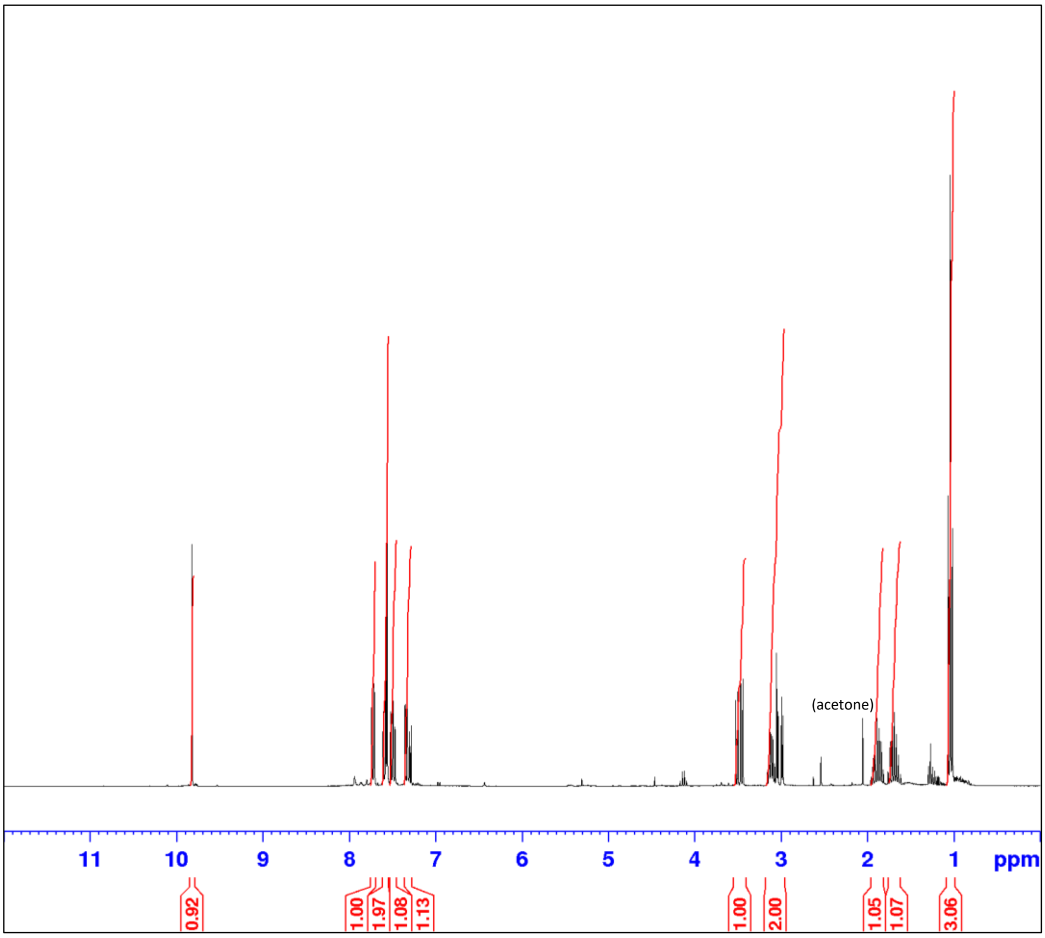

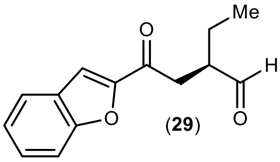

(**29**)

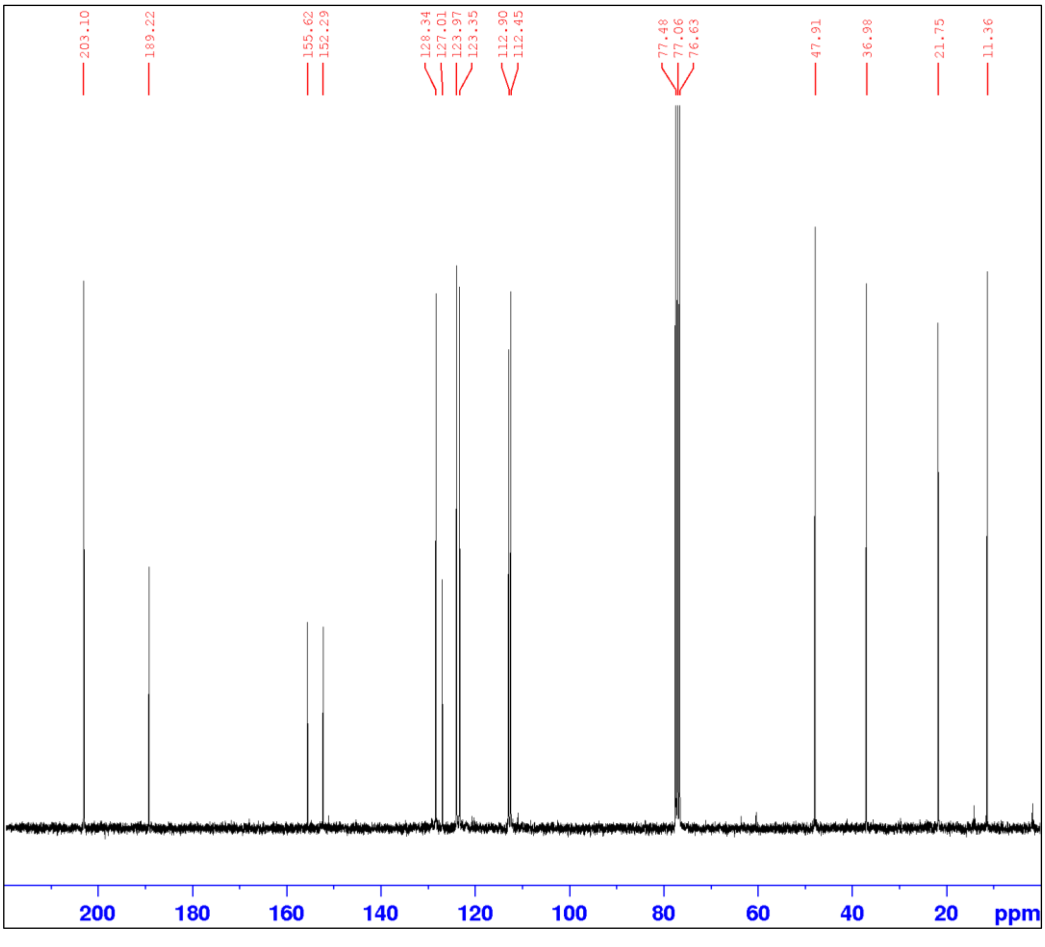

75 MHz, $CDCl_3$

HPLC chromatograms

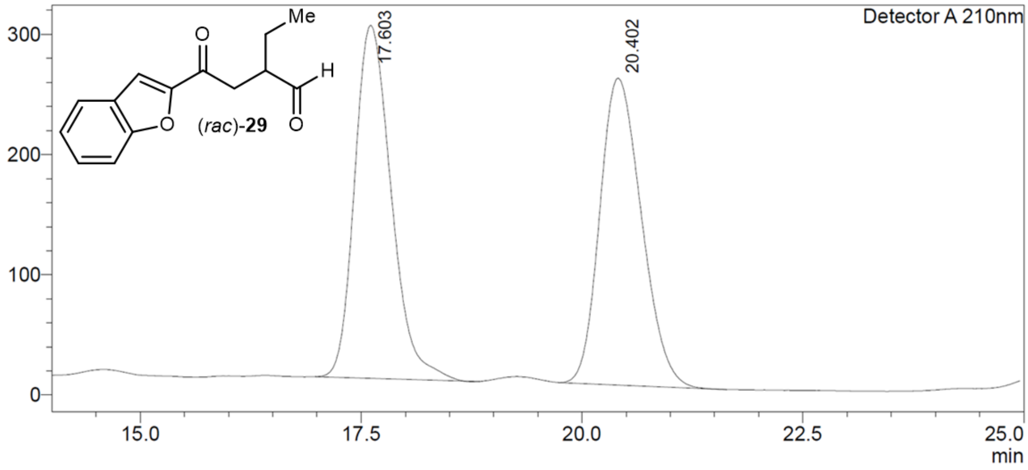

Peak#	Ret. Time	Area	Height	Area%
1	17.603	8473957	293589	50.240
2	20.402	8392905	255223	49.760
Total		16866862	548812	100.000

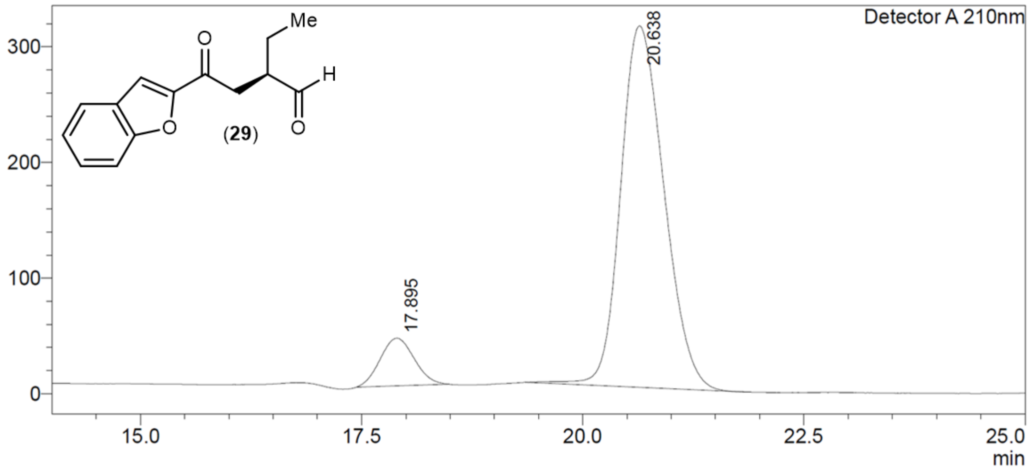

Peak#	Ret. Time	Area	Height	Area%
1	17.895	1107327	41242	9.386
2	20.638	10689822	312528	90.614
Total		11797149	353771	100.000

300 MHz, $CDCl_3$

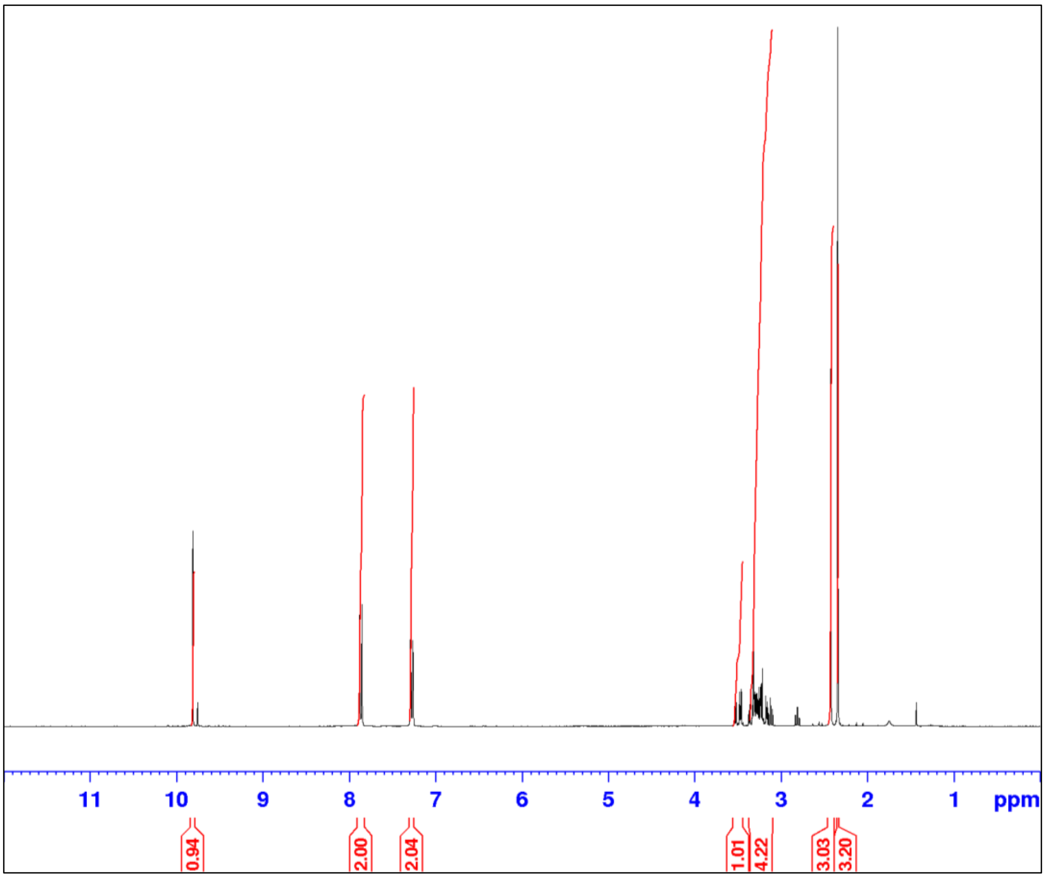

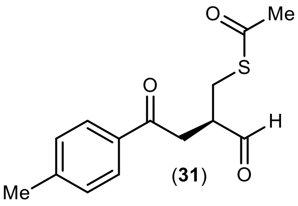

(**31**)

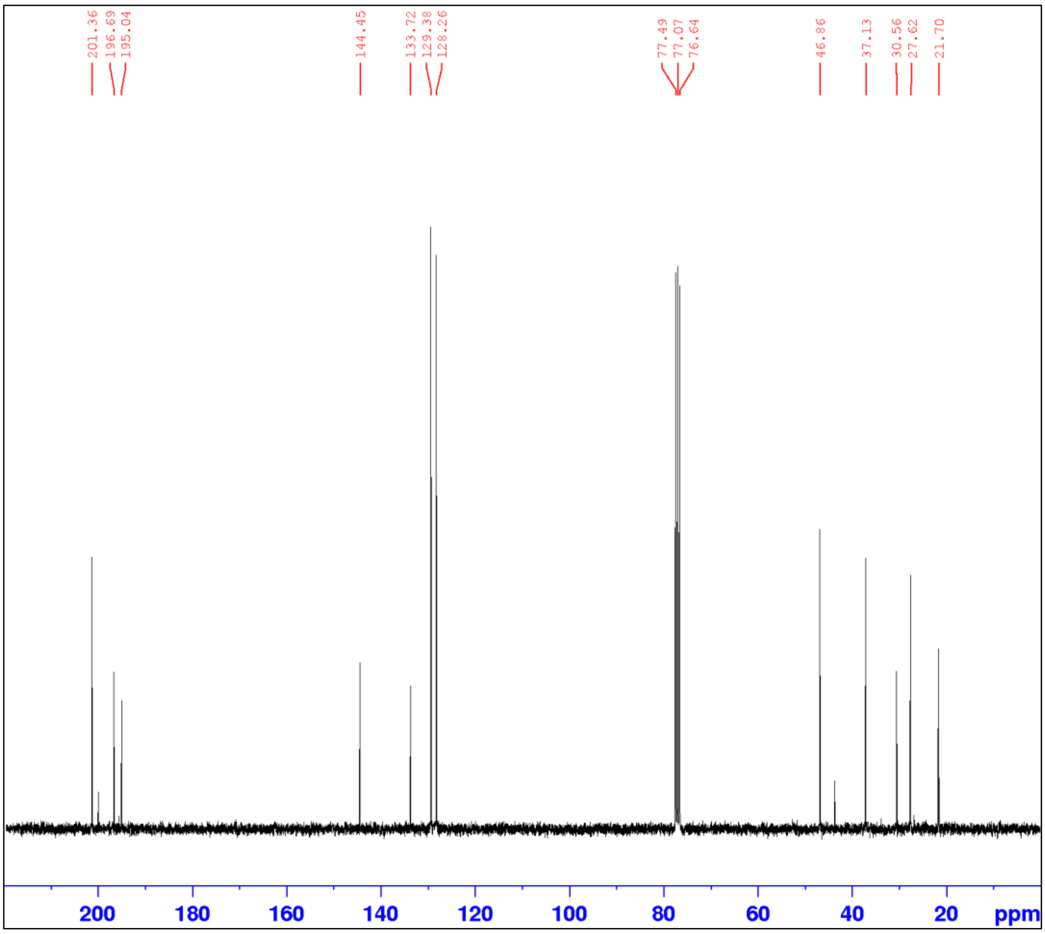

75 MHz, $CDCl_3$

HPLC chromatograms

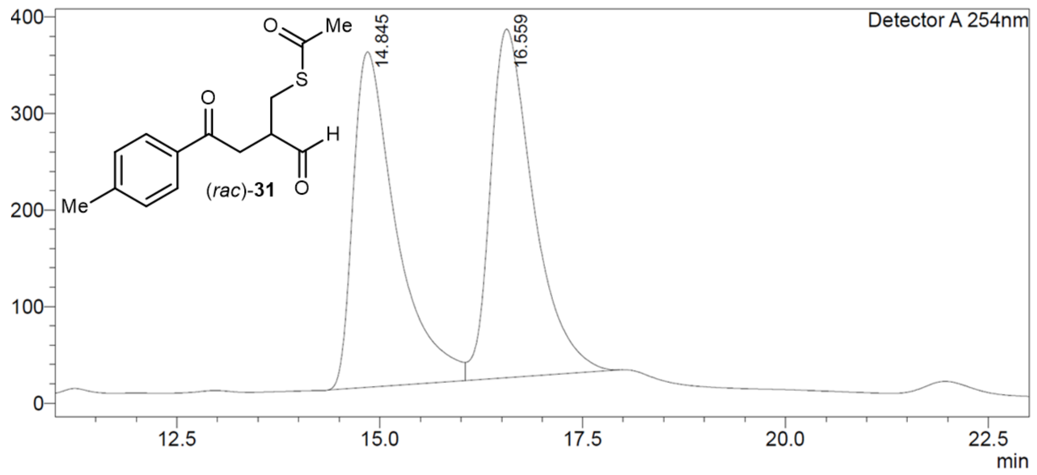

Peak#	Ret. Time	Area	Height	Area%
1	14.845	12848930	347520	48.981
2	16.559	13383451	360849	51.019
Total		26232381	708369	100.000

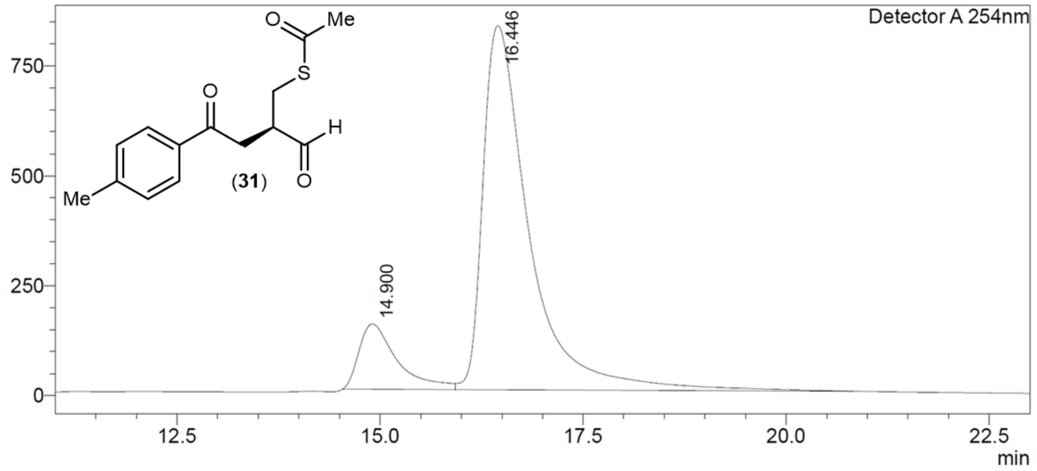

Peak#	Ret. Time	Area	Height	Area%
1	14.900	4282915	146061	11.505
2	16.446	32944351	828038	88.495
Total		37227265	974099	100.000

8. References

(S1) C. Rosso, J. D. Williams, G. Filippini, M. Prato and C. O. Kappe, *Org. Lett.*, **2019**, *21*, 5341-5345.

(S2) A.-H. Li, S. Moro, N. Forsyth, N. Melman, X.-d. Ji and K. A. Jacobson, *J. Med. Chem.*, **1999**, *42*, 706-721.

(S3) E. Arceo, I. D. Jurberg, A. Álvarez-Fernández and P. Melchiorre, *Nat. Chem.*, **2013**, *5*, 750-756.

(S4) H.-Y. Jang, J.-B. Hong and D. W. C. MacMillan, *J. Am. Chem. Soc.*, **2007**, *129*, 7004-7005.

(S5) J. Zhang, C. Xing, B. Tiwari and Y. R. Chi, *J. Am. Chem. Soc.*, **2013**, *135*, 8113-8116.

(S6) Y. Kuang, K. Wang, X. Shi, X. Huang, E. Meggers and J. Wu, *Angew. Chem. Int. Ed.*, **2019**, *58*, 16859-16863.

(S7) Y. Guo, Y. Lou, J. Chen and Y. Zhao, *ChemSusChem*, **2022**, *15*, e202102334.
